# The entry reaction of the plant shikimate pathway is subjected to highly complex metabolite-mediated regulation

**DOI:** 10.1093/plcell/koaa042

**Published:** 2021-01-07

**Authors:** Ryo Yokoyama, Marcos V V de Oliveira, Bailey Kleven, Hiroshi A Maeda

**Affiliations:** Department of Botany, University of Wisconsin–Madison, 430 Lincoln Dr. Madison, WI 53706, USA

## Abstract

The plant shikimate pathway directs bulk carbon flow toward biosynthesis of aromatic amino acids (AAAs, i.e. tyrosine, phenylalanine, and tryptophan) and numerous aromatic phytochemicals. The microbial shikimate pathway is feedback inhibited by AAAs at the first enzyme, 3-deoxy-d-*arabino*-heptulosonate 7-phosphate synthase (DHS). However, AAAs generally do not inhibit DHS activities from plant extracts and how plants regulate the shikimate pathway remains elusive. Here, we characterized recombinant *Arabidopsis thaliana* DHSs (AthDHSs) and found that tyrosine and tryptophan inhibit AthDHS2, but not AthDHS1 or AthDHS3. Mixing AthDHS2 with AthDHS1 or 3 attenuated its inhibition. The AAA and phenylpropanoid pathway intermediates chorismate and caffeate, respectively, strongly inhibited all AthDHSs, while the arogenate intermediate counteracted the AthDHS1 or 3 inhibition by chorismate. AAAs inhibited DHS activity in young seedlings, where *AthDHS2* is highly expressed, but not in mature leaves, where *AthDHS1* is predominantly expressed. Arabidopsis *dhs1* and *dhs3* knockout mutants were hypersensitive to tyrosine and tryptophan, respectively, while *dhs2* was resistant to tyrosine-mediated growth inhibition. *dhs1* and *dhs3* also had reduced anthocyanin accumulation under high light stress. These findings reveal the highly complex regulation of the entry reaction of the plant shikimate pathway and lay the foundation for efforts to control the production of AAAs and diverse aromatic natural products in plants.

## Teaser

Characterization of Arabidopsis 3-deoxy-D-*arabino*-heptulosonate 7-phosphate synthase enzymes and mutants revealed highly complex metabolite-mediated feedback regulation of the plant shikimate pathway.

## Introduction

**Figure:**
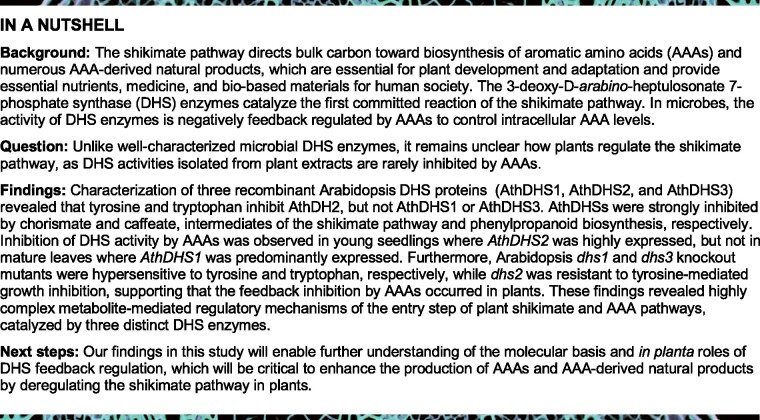


**Figure 1:**
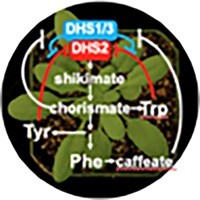
The shikimate pathway and biosynthesis of AAAs in plants. The shikimate pathway leads to biosynthesis of AAAs, which are not only required for protein synthesis but also used as precursors of numerous AAA-derived natural products (green letters) in plants. The gray lines with an arrowhead or a hash indicate known feedback activation or inhibition of the post-chorismate pathways by AAAs, respectively, while double arrows denote the multiple reactions. DHS, 3- deoxy-D-*arabino*-heptulosonate 7-phosphate synthase; EPSPS, 5-*enol*pyruvylshikimate-3-phosphate synthase; AS, anthranilate synthase; CM, chorismate mutase; TyrA, arogenate dehydrogenase; ADT, arogenate dehydratase; E4P, D-erythrose 4-phosphate; PEP, phospho*enol*pyruvate.

The shikimate pathway directs carbon flow from central carbon metabolism to the biosynthesis of aromatic amino acids (AAAs)—l-tyrosine, l-phenylalanine, and l-tryptophan (Tyr, Phe, and Trp, respectively)—and numerous aromatic natural products. Since AAAs are required for protein synthesis in all organisms but animals lack the shikimate pathway, AAAs are essential human nutrients and the shikimate pathway is the target of the most widely used herbicide, glyphosate (Shah et al., 1986; Klee et al., 1987; Pollegioni et al., 2011; Hildebrandt et al., 2015; Figure 1). Plant natural products derived from the shikimate and AAA pathways play critical roles in plant physiology and adaptation and are widely used as nutraceuticals, pharmaceuticals, and biomaterials (Herrmann, 1995; Tzin and Galili, 2010; Maeda and Dudareva, 2012; Figure 1). Shikimate pathway intermediates are used to synthesize hydrolysable tannins and chlorogenic acids, but also to produce the anti-influenza virus agent Tamiflu (Niggeweg et al., 2004; Barbehenn and Peter Constabel, 2011; Ghosh et al., 2012). Trp is a precursor of auxin phytohormones, plant defense compounds (e.g. indole alkaloids and glucosinolates), and cancer drugs such as glucobrassicin and vincristine produced in Brassicaceae and *Catharanthus roseus*, respectively (De Luca and St Pierre, 2000; Kliebenstein, 2011; Saini et al., 2013; Sanchez-Pujante et al., 2017). Phenylpropanoids derived from Phe are the largest class of plant natural products, next to terpenoids, and include flavonoids, anthocyanin pigments, tannins, the principal cell wall component lignin, etc. (Boerjan et al., 2010; Vogt, 2010). As lignin can account for up to 30% of deposited carbon in vascular plants (Razal et al., 1996; Boerjan et al., 2010), plants direct a significant portion of carbon flow through the shikimate pathway. Despite the paramount and broad importance of these aromatic plant natural products in both plants and humans, little is known about how the shikimate pathway is regulated in plants, which represents a major knowledge gap in plant biology and biochemistry.

The shikimate pathway converts phospho*enol*pyruvate (PEP) and d-erythrose 4-phosphate (E4P) from glycolysis and the pentose phosphate pathways, respectively, into chorismate, the final common precursor of all AAAs. The 3-deoxy-d-*arabino*-heptulosonate 7-phosphate synthase enzyme (DHS, also known as DAHP synthase) catalyzes the first committed reaction of the shikimate pathway, which is followed by six additional steps to produce chorismate (Bentley, 1990; Herrmann, 1995; Tzin and Galili, 2010; Maeda and Dudareva, 2012). Chorismate is located at a critical branch point into Phe/Tyr and Trp biosynthesis (Figure 1). For Trp biosynthesis, chorismate is first converted by anthranilate synthase (AS) to anthranilate, which is used to synthesize Trp, indole, and other natural products (Radwanski and Last, 1995; Romero et al., 1995b; Raboni et al., 2009). For biosynthesis of Phe and Tyr, chorismate is first converted by chorismate mutase (CM) into prephenate, from which the Tyr and Phe pathways diverge in most microbes (Bentley, 1990). In plants, prephenate is first transaminated to arogenate (Bonner and Jensen, 1985; Siehl et al., 1986; Graindorge et al., 2010; Dal Cin et al., 2011), and then converted into Tyr and Phe by arogenate dehydrogenases and dehydratases (known as TyrA and ADT), respectively (Romero et al., 1995b; Cho et al., 2007). Thus, plants and microbes use different pathways to produce Tyr and Phe, in which arogenate serves as the last common precursor and is located at a critical branch point of Tyr and Phe biosynthesis (Figure 1).

Like most amino acid biosynthetic pathways (Galili et al., 2016), AAA biosynthesis is subjected to strict feedback regulation (Tzin and Galili, 2010; Maeda and Dudareva, 2012). The committed enzymes of plant individual AAA biosynthesis, TyrA, ADT, and AS, are inhibited by the corresponding final products, Tyr, Phe, and Trp, respectively (Siehl and Conn, 1988; Bohlmann et al., 1996; Rippert and Matringe, 2002a; Cho et al., 2007). In some cases, Tyr activates ADT to redirect carbon flow from Tyr to Phe (Jung et al., 1986; Siehl and Conn, 1988). CM can be inhibited by Tyr and Phe and activated by Trp to redirect flux from Trp to Phe/Tyr biosynthesis (Figure 1; Benesova and Bode, 1992; Romero et al., 1995a). De-regulation of CM, AS, ADT, and TyrA leads to elevated accumulation of respective downstream AAAs in both microbes and plants (Li and Last, 1996; Baez-Viveros et al., 2004; Lutke-Eversloh and Stephanopoulos, 2005; Chavez-Bejar et al., 2008; Yamada et al., 2008; Huang et al., 2010; Shen et al., 2012; de Oliveira et al., 2019). Therefore, the feedback regulation of post-chorismate AAA biosynthetic enzymes is generally conserved among plants and microbes and plays critical roles in controlling the production of individual AAAs.

Unlike the well-characterized regulation within the post-chorismate pathway, the regulation of the flux into and through the upstream shikimate pathway is poorly understood in plants (Herrmann, 1995; Tzin and Galili, 2010; Maeda and Dudareva, 2012; Lynch and Dudareva, 2020). In microbes, the DHS-mediated entry reaction of the shikimate pathway is feedback regulated by AAAs (Bentley, 1990). *Escherichia coli* (*E. coli*) has three DHS enzymes, AroF, AroG, and AroH, which are allosterically inhibited by Tyr, Phe, and Trp, respectively (Schoner and Herrmann, 1976; Shumilin et al., 1999). *Saccharomyces cerevisiae* has two DHS enzymes, Aro3 and Aro4, which are inhibited by Tyr and Phe, respectively (Paravicini et al., 1989; Hartmann et al., 2003).

The AAA sensitivity of DHS activity from various plant tissues has been analyzed extensively, but has yielded surprising and puzzling findings: AAAs do not generally inhibit plant DHS activity, and there is no report that Phe affects plant DHS activity (Huisman and Kosuge, 1974; Pinto et al., 1986; Sharma et al., 1993; Herrmann, 1995; Maeda and Dudareva, 2012). Some exceptions include activation of carrot (*Daucus carota*) DHS activity by Trp (Suzich et al., 1985) and inhibition of DHS activities from maize (*Zea mays*) shoots and pea (*Pisum sativum*) leaves in the presence of 1 mM Trp and Tyr by 50% and 60%, respectively (Graziana and Boudet, 1980; Reinink and Borstlap, 1982). Also, arogenate was reported to inhibit DHS activity from mung bean (*Vigna radiata*) seedlings and spinach (*Spinacia oleracea*) leaves though beyond 0.4 and 1 mM, respectively (Rubin and Jensen, 1985; Doong et al., 1993). However, the intracellular concentration of arogenate is likely lower than one that can significantly affect the DHS activity (Razal et al., 1994; Herrmann, 1995). Nevertheless, expression of a feedback-insensitive bacterial *DHS* mutant enzymes in Arabidopsis and tomato (*Solanum lycopersicum*) led to elevated accumulation of all three AAAs, shikimate, and some phenylpropanoids (Tzin et al., 2012, 2013). Therefore, the production of AAAs and their downstream compounds is also limited at the DHS-catalyzed reaction in plants, though the underlying mechanism and key inhibitor or activator molecule(s) involved remain unknown.

There are two types of DHS enzymes: Type I DHSs are found in most fungi and bacteria including *E. coli*, yeast, and cyanobacteria, whereas type II DHSs are mainly found in plants and some bacteria such as *Mycobacterium tuberculosis* (Gosset et al., 2001; Richards et al., 2006; Tohge et al., 2013). Although their reaction mechanisms are similar (e.g. metal requirements; Walker et al., 1996; Shumilin et al., 1999; Hartmann et al., 2003; Webby et al., 2010), type II DHSs appear to have more complex regulatory mechanisms than type I enzymes. Unlike type I DHSs having a single allosteric effector binding site (Shumilin et al., 1999; Hartmann et al., 2003), *M. tuberculosis* type II DHS has at least two effector binding sites for Phe and Trp, and are inhibited by the combination of Phe and Trp, but not by individual ones (Webby et al., 2010). *Arabidopsis thaliana* has three type II DHS enzymes, AthDHS1, 2, and 3, which are encoded by AT4G39980, AT4G33510, and AT1G22410, respectively (Supplemental Figure S1; Entus et al., 2002; Richards et al., 2006; Tohge et al., 2013). AthDHS1 has been biochemically characterized as recombinant protein and shows strict dependency on Mn^2+^ cofactor and reducing conditions [i.e. the presence of dithiothreitol (DTT)] (Entus et al., 2002). This likely links AthDHS1 activity to photosynthetic electron transport in the plastids, where the shikimate pathway and the upstream pentose phosphate pathways are localized (Mousdale and Coggins, 1985; Dietz and Hell, 2015). The biochemical properties of AthDHS2 and AthDHS3 and the metabolite-mediated feedback regulation of Arabidopsis DHS enzymes remain to be explored.

To understand the regulatory mechanisms of the committed step of the shikimate pathway, here we generated recombinant enzymes of all three DHS isoforms of *A. thaliana* and conducted their detailed biochemical characterization. All three AthDHSs had similar kinetic parameters; however, Tyr and Trp specifically inhibited AthDHS2, but not AthDHS1 and AthDHS3. We further identified several other pathway intermediates including chorismate and caffeate, that strongly inhibit all AthDHS isoforms. DHS activity of Arabidopsis and spinach leaf extracts were not inhibited by AAAs, consistent with prior reports (Huisman and Kosuge, 1974; Pinto et al., 1986; Sharma et al., 1993), but this was due to lower expression of *AthDHS2* and the loss of the AthDHS2 AAA sensitivity in the presence of AAA-insensitive AthDHS1. Analyses of Arabidopsis *dhs* knockout mutants further showed some distinct roles of AthDHS isoforms *in planta*. Together, these biochemical and genetic data revealed the highly complex metabolite-mediated regulatory mechanisms of the entry step of plant shikimate and AAA pathways, catalyzed by three distinct DHS enzymes.

## Results

### Biochemical characterization of three Arabidopsis DHS enzymes

**Figure 2:**
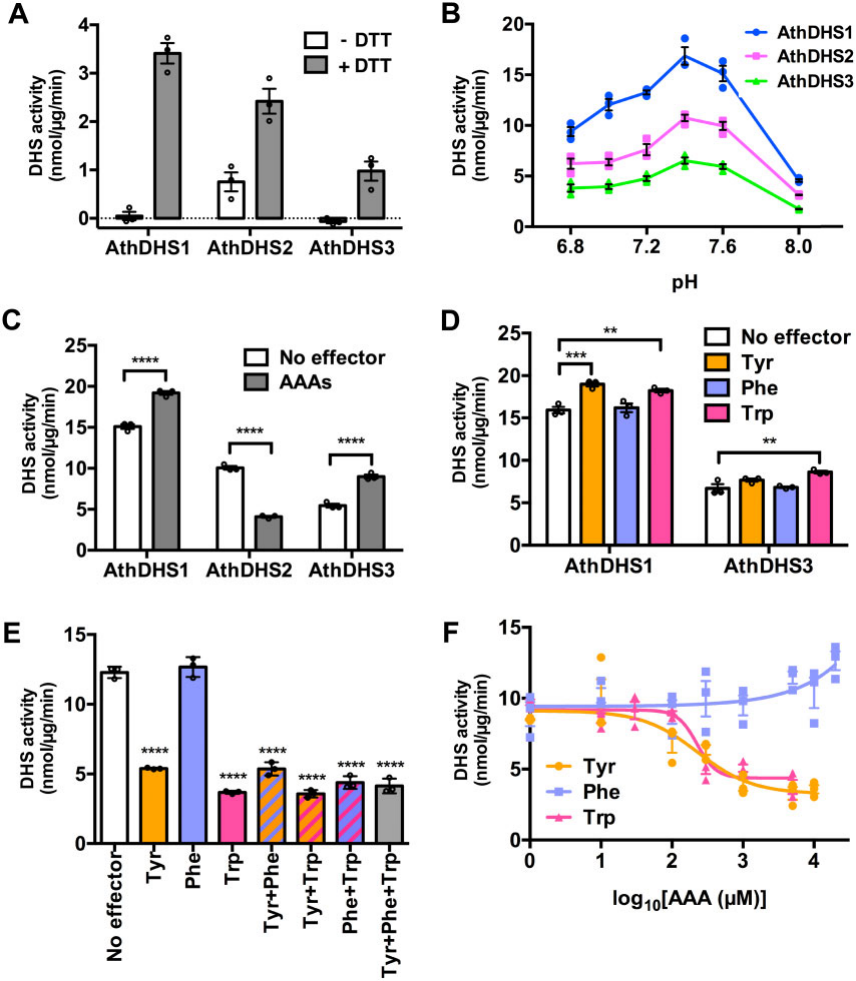
Tyr and Trp exert inhibitory effects only on AthDHS2 activity. (A) Requirement of DTT for different AthDHS isoforms. DHS activity was monitored with or without DTT in the presence of 1 mM Mn^2+^ at pH 7.0. (B) Optimal pH of the recombinant AthDHS enzymes. Enzymatic assays of AthDHS1, AthDHS2, and AthDHS3 were conducted using HEPES buffer at various pH. (C) DHS activity assay of AthDHS1, 2, and 3 in the presence of the AAA mixture at 1 mM each. (D) AthDHS1 and AthDHS3 activity assays in the presence of individual AAAs at 1 mM. E, AthDHS2 activity in the presence of individual or combination of different AAAs. F, IC_50_ curves of AthDHS2 with varied concentrations of Tyr, Phe, or Trp. **P ≤ 0.01 and ****P ≤ 0.0001 denote significant differences by Student’s *t* test (C) or one-way ANOVA (D, E) against the “No effector” control of respective isoforms. Data are means ± SEM (*n* = 3 replicated reactions). All the individual data points are shown as dots.

To biochemically characterize plant DHSs, all three Arabidopsis DHS enzymes, AthDHS1, AthDHS2, and AthDHS3, were expressed as hexa-histidine tagged recombinant proteins in *E*. *coli* and purified using affinity chromatography. To test metal and reducing agent requirements, DHS assays were conducted in the presence and absence of DTT and different metal ions at pH 7. All three AthDHSs showed the highest activity with Mn^2+^ and DTT (Figure 2, A and Supplemental Figure S2), consistent with the prior report for AthDHS1 (Entus et al., 2002). No DHS activity was detectable in AthDHS1 and AthDHS3 without DTT, whereas some residual DTT-independent activity was detected in AthDHS2 (Figure 2, A). Similar results were obtained at pH 8 (Supplemental Figure S2). Other divalent cations, Co^2+^, Cd^2+^, Cu^2+^, and Zn^2+^, used by *M. tuberculosis* DHS (Webby et al., 2005), were also tested, but only Cd^2+^ partially supported DHS activity in all three AthDHSs at 20%–50% of corresponding Mn^2+^-dependent activity (Supplemental Figure S2). Co^2+^-dependent DHS activity, previously detected from plant tissue extracts (Rubin and Jensen, 1985; Ganson et al., 1986; Morris et al., 1989; Doong et al., 1992), was not observed in any of the AthDHS recombinant enzymes (Supplemental Figure S2). DHS assays conducted at different pH from 6.8 to 8.0 showed the maximum activity at pH 7.4 for all three AthDHSs (Figure 2, B), consistent with the previous report on DHS activity from maize, potato (*Solanum tuberosum*), and spinach tissue extracts (Graziana and Boudet, 1980; Pinto et al., 1986; Doong et al., 1993). Thus, all three AthDHSs require a reducing agent, prefer Mn^2+^ as a metal cofactor, and have an optimal pH of 7.4.

To check potential contamination of *E*. *coli* DHS enzymes in our enzyme preparation, DHS assays were conducted using *E*. *coli* cells carrying the empty or AthDHS1 vector in the presence of Mn^2+^ or Fe^2+^, as Fe^2+^ supports the activity of *E. coli* DHS (Stephens and Bauerle, 1991) but not AthDHS1 (Entus et al., 2002). Unlike in their crude supernatant, no Fe^2+^-dependent DHS activity was detected in either sample after purification, whereas only the purified AthDHS1 sample showed Mn^2+^-dependent activity (Supplemental Figure S3), confirming that *E. coli* DHS activity was effectively removed to undetectable levels.

Steady-state enzyme kinetic analyses were then conducted using various concentrations of E4P and PEP substrates (Table 1 and Supplemental Figure S4). All reactions followed Michaelis–Menten kinetics, with the exception of AthDHS2, which was inhibited at high E4P concentrations beyond 4 mM (Supplemental Figure S4, C). All three AthDHSs showed much higher *K*_m_ toward E4P (1.6–2.8 mM) than PEP (250–706 µM, Table 1), consistent with previous reports in spinach and carrot (Doong et al., 1992; Suzuki et al., 1996). Overall catalytic efficiency (*k*_cat_/*K*_m_) was the highest for AthDHS1 followed by AthDHS2 and then AthDHS3 (62, 25, and 12 /s/mM, respectively, for PEP), which reflect their turnover rates (*k*_cat_, Table 1). Although AthDHS1 is the most efficient enzyme, all three AthDHSs overall showed similar ranges of *K*_m_ and *k*_cat_.

**Table 1:** Kinetic parameters of *A. thaliana* DHS1, DHS2, and DHS3 enzymes

	Substrate	*V*_max_	*K*_m_	*k*_cat_	*k*_cat_/*K*_m_
		(nmol/µM/min)	(µM)	(/s)	(/s/mM)
AthDHS1	E4P	17.8 ± 0.59	2842 ± 910	16.4 ± 0.54	7.06 ± 2.1
	PEP	15.3 ± 0.62	250 ± 53	14 ± 0.57	61.9 ± 14
AthDHS2	E4P	12.9 ± 1.8	1755 ± 400	11.8 ± 1.6	7.02 ± 0.72
	PEP	9.81 ± 0.48	360 ± 34	8.95 ± 0.44	25.2 ± 2.4
AthDHS3	E4P	9.92 ± 0.31	1550 ± 276	9.13 ± 0.28	6.35 ± 0.16
	PEP	9.15 ± 0.27	706 ± 124	8.42 ± 0.25	12.4 ± 0.39

Kinetic parameters were obtained from the Michaelis–Menten curves obtained by DHS activity measured using various concentrations of the E4P or PEP substrate (Supplemental Figure S4). Since AthDHS2 exhibited substrate inhibition by E4P, the Michaelis–Menten kinetics curves were generated by assuming that those curves represent the activity that would be found if no substrate inhibition occurred (Bernstein et al., 1978). To estimate the *K*_m_ and *V*_max_ values, data points at high substrate concentrations were plotted according to the Lineweaver–Burk plots. Data are means ± SEM (*n *=* *3 replicated reactions).

### Tyrosine and tryptophan inhibit Arabidopsis DHS2, but not DHS1 and 3

To test if plant DHS enzymes are regulated by AAAs, like microbial DHSs (Bentley, 1990), recombinant AthDHS activity was monitored in the presence of AAAs. Since *M. tuberculosis* DHS, a close microbial homolog of plant DHSs, requires both Trp and Phe for its inhibition, we first used the mixture of three AAAs (Tyr, Phe, and Trp) at 1 mM. The activity of AthDHS1 and AthDHS3 was slightly activated by the AAA mixture up to 120%, whereas AthDHS2 activity was significantly inhibited (*P *<* *0.001, Figure 2, C). When individual AAAs were tested, AthDHS1 was significantly activated by Tyr and Trp, whereas only Trp activated AthDHS3 (Figure 2, D). In contrast, 60%–70% of AthDHS2 activity was inhibited by Tyr or Trp, but not by Phe (Figure 2, E). The combination of Tyr and Trp had no additive inhibitory effect on AthDHS2 activity. The addition of Phe had no major effect on the AthDHS2 inhibition by Tyr or Trp (Figure 2, E). AthDHS2 assays with varying concentrations of individual AAAs ranging from 1 µM to at least 5 mM further revealed that Tyr and Trp, but not Phe, inhibit AthDHS2 with the IC_50_ values of 230.4 and 225.1 µM, respectively (Figure 2, F). Phe slightly activated the AthDHS2 activity at a very high concentration but only beyond 5 mM (Figure 2, F). The other 17 proteinogenic amino acids did not significantly alter activities of any AthDHS isoforms (Supplemental Figure S5). Taken together, these observations show that Tyr and Trp act as effective inhibitors of AthDHS2 and slightly activate AthDHS1 and AthDHS3.

### Chorismate, the last common precursor of all AAAs, strongly inhibits all three AthDHS enzymes

**Figure 3:**
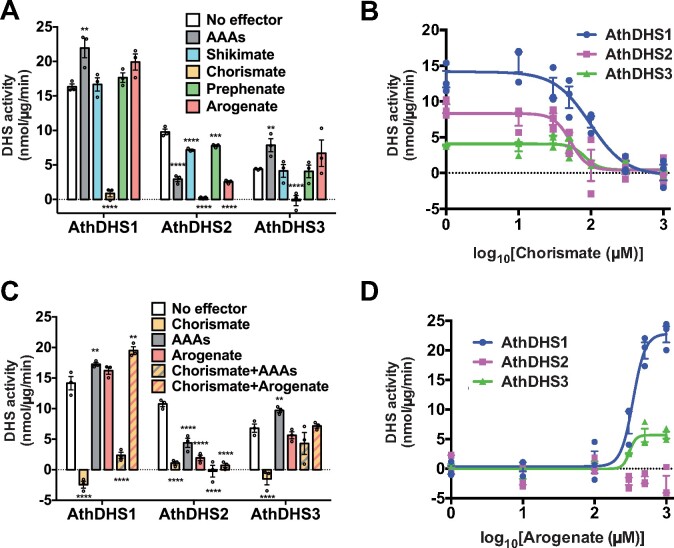
Chorismate-dependent inhibition of AthDHS was offset by arogenate. (A) DHS activity assays of AthDHS1, 2, and 3 in the presence of 1 mM shikimate, chorismate, prephenate, and arogenate. (B) IC_50_ curves of AthDHS1, 2, and 3 with varied concentrations of chorismate. (C) DHS assays of AthDHS1, 2, and 3 in the presence of AAAs or arogenate with or without chorismate, all at 1 mM. (D) IC_50_ curves of AthDHS1, 2, and 3 with varied concentrations of arogenate in the presence of 1 mM chorismate. **P ≤ 0.01, ***P ≤ 0.001, and ****P ≤ 0.0001 denote significant differences by one-way ANOVA against corresponding “No effector” control samples. Data are means ± SEM (*n* = 3 replicated reactions). All the individual data points are shown as dots.

To identify additional effector molecules, besides AAAs, that may affect DHS activity, we tested key intermediate compounds within the shikimate and AAA biosynthetic pathways: shikimate, chorismate, prephenate, and arogenate (Figure 1). Shikimate, prephenate, and arogenate at 1 mM did not significantly affect the activity of AthDHS1 and AthDHS3, while AAAs again slightly activated them (Figure 3, A). On the other hand, the activity of AthDHS2 was inhibited by shikimate, prephenate, and arogenate by approximately 30%, 25%, and 75%, respectively (Figure 3, A). Notably, all three AthDHSs were completely inhibited by chorismate at 1 mM (Figure 3, A). DHS assays at varied concentrations of chorismate ranging from 1 µM to 1 mM showed that AthDHS1, AthDHS2, and AthDHS3 are inhibited by chorismate at the IC_50_ values of 97.3, 52.5, and 83.0 µM, respectively (Figure 3, B).

Since commercially available chorismate reagents include impurities, we further evaluated if chorismate is indeed the inhibitor that reduces DHS activity. Before adding to the DHS assays, the chorismate solution was incubated with the active or boiled CM2 enzyme of Arabidopsis (AthCM2), which specifically converts chorismate into prephenate (Westfall et al., 2014). Untreated chorismate completely inhibited AthDHS1 activity as expected, but chorismate that was incubated with active AthCM2 did not (Supplemental Figure S6, A). Chorismate that was treated with boiled AthCM2 exhibited the same inhibitory effect as one with the untreated chorismate (Supplemental Figure S6, A). These results confirm that chorismate indeed inhibits AthDHS activity.

### Arogenate counteracts the chorismate-mediated inhibition of AthDHS1 and AthDHS3

Next, we examined if other effector molecules potentially exert additive or synergistic effects on the chorismate-mediated inhibition of AthDHSs. When the AAA mixture was combined with chorismate, chorismate-inhibited AthDHS1 and AthDHS3 activity recovered slightly to one-fifth and half, respectively, of their corresponding activity without any effector molecules (Figure 3, C). Notably, when arogenate was provided with chorismate (both at 1 mM), chorismate-inhibited AthDHS1 and AthDHS3 activity fully recovered to the levels equivalent to no-effector controls (Figure 3, C). The chorismate-inhibited AthDHS2 activity was not recovered by the combination of chorismate and arogenate (Figure 3, C and D), likely because arogenate by itself also inhibits AthDHS2 (Figure 3, A). The other shikimate pathway intermediates, shikimate and prephenate, did not attenuate chorismate-dependent inhibition of AthDHS1 (Supplemental Figure S7). An assay using various concentrations of arogenate in the presence of 1 mM chorismate revealed that arogenate offsets the chorismate-mediated inhibition of AthDHS1 and AthDHS3 activity with the IC_50_ values of 343.9 and 305.4 µM, respectively (Figure 3, D).

Since arogenate is not commercially available, we prepared the arogenate reagent through transamination of prephenate with aspartate (see the “Materials and methods” section; Maeda et al., 2010; Schenck et al., 2015), which may still be present in the arogenate preparation and contribute to the above observed effect. However, unlike arogenate, 1 mM prephenate or aspartate did not affect the DHS activity regardless of the presence of chorismate (Supplemental Figure S7, A). Also, before adding it to the assays, we incubated arogenate with hydrochloric acid (HCl), which converts arogenate into Phe (Gilchrist and Connelly, 1987). The AthDHS1 reaction containing the HCl-treated arogenate and chorismate did not offset chorismate-mediated DHS inhibition and still showed no enzymatic activity, like that with only chorismate (Supplemental Figure S6, B). These results together revealed that arogenate counteracts the inhibition of AthDHS by chorismate.

### Caffeate and its derivatives in phenylpropanoid biosynthesis inhibit all three AthDHS enzymes

**Figure 4:**
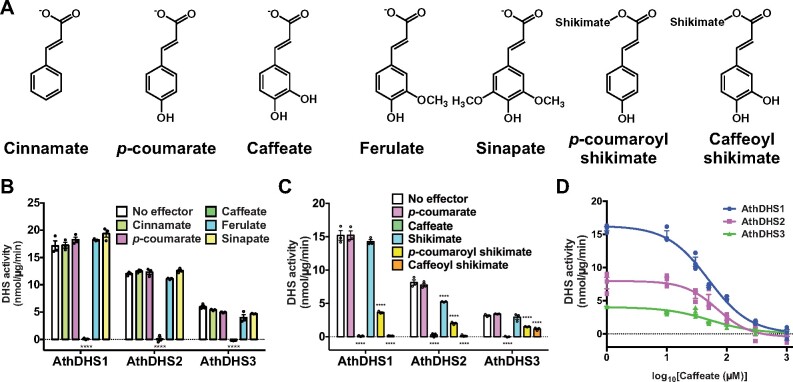
Caffeate and its derivative inhibit AthDHS activity. (A) Chemical structures of cinnamate, *p*-coumarate, caffeate, ferulate, sinapate, *p*-coumaroyl shikimate, and caffeoyl shikimate. (B) DHS activity assays of AthDHS1, 2, and 3 in the presence of 1 mM cinnamate, *p*-coumarate, caffeate, ferulate, or sinapate. (C) DHS assays of AthDHS1, 2, and 3 in the presence of 1 mM *p*-coumarate, caffeate, shikimate, *p*-coumaroyl shikimate, and caffeoyl shikimate. (D) IC_50_ curves of AthDHS1, 2, and 3 with varied concentrations of caffeate. ****P ≤ 0.0001 denotes significant differences by one-way ANOVA against corresponding “No effector” control samples. Data are means ± SEM (*n* = 3 replicated reactions). All the individual data points are shown as dots.

Since Phe itself did not significantly affect activity of all AthDHS isoforms (Figure 2, D and E), the question still remains: how do DHS enzymes monitor the pathway activity of the Phe branch of AAA biosynthesis? To address this question, we tested five intermediate compounds in the downstream phenylpropanoid pathway for their effects on AthDHS activity (Figure 4, A). Although cinnamate, *p-*coumarate, ferulate, and sinapate had no effects on all AthDHSs at 1 mM, caffeate completely inhibited activities of all three AthDHSs (Figure 4, B). Caffeoyl shikimate, one of the major derivatives of caffeate in plants (Boerjan et al., 2010; Vogt, 2010), also fully inhibited AthDHS1 and 2 and reduced AthDHS3 activity by 70% (Figure 4, C). Although *p-*coumarate by itself had no effect, *p-*coumaroyl shikimate partially reduced the activity of all AthDHSs by 66%–75% (Figure 4, C). DHS assays of individual DHS isoforms with varying concentrations of caffeate further showed that AthDHS1, 2, and 3 are inhibited by caffeate with IC_50_ values of 49.7, 69.2, and 53.4 µM for AthDHS1, 2, and 3, respectively (Figure 4, D). Thus, the phenylpropanoid intermediates, caffeate and its derivative, effectively inhibit all three DHS enzymes of Arabidopsis.

### Expression pattern and ratio of AAA-inhibited versus non-inhibited DHS isoforms determine AAA sensitivity of DHS activity detected from plant tissues

**Figure 5:**
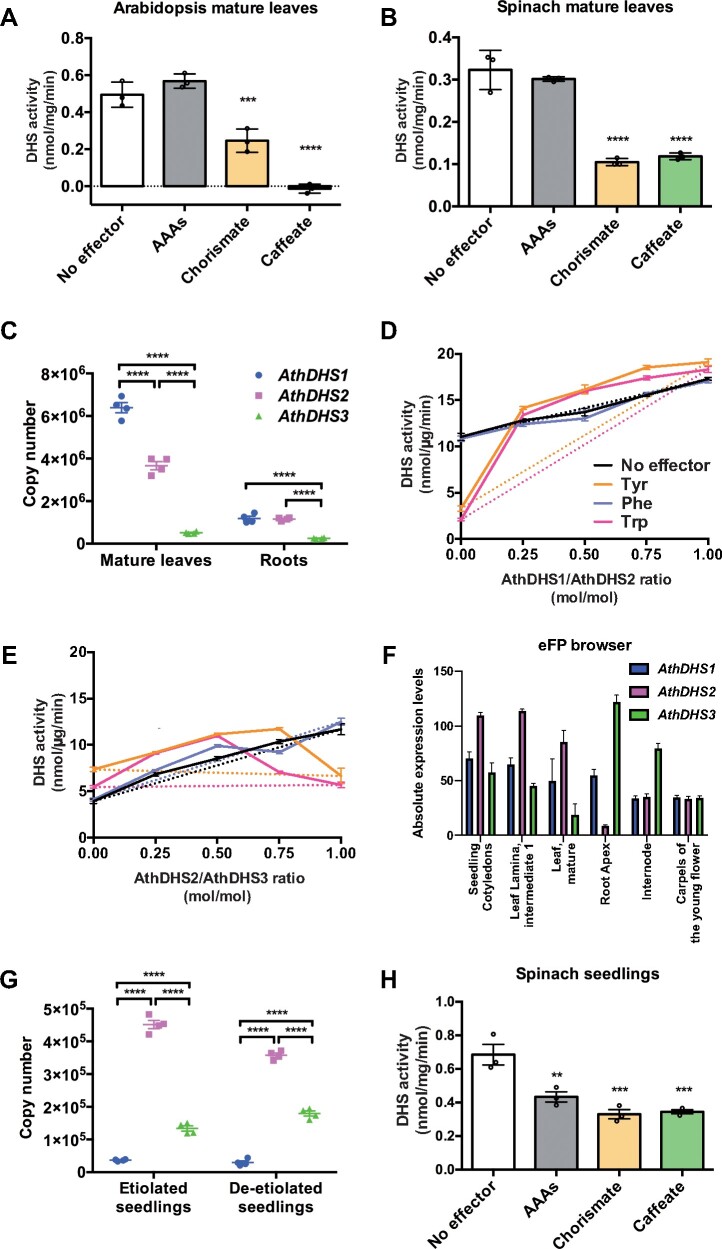
AAA inhibition of DHS activity is tissue type dependent. (A and B) DHS activity assay of crude extracts from Arabidopsis (A) and spinach (B) mature leaves in the presence of 1 mM AAAs, chorismate, or caffeate. (C) Absolute copy numbers of *AthDHS1*, *AthDHS2*, and *AthDHS3* genes expressed in Arabidopsis Col-0 mature leaves and roots. (D and E) DHS assays using the mixtures of the AthDHS1 and AthDHS2 recombinant enzymes (D) and the AthDHS2 and AthDHS3 recombinant enzymes (E) with indicated molar ratios at *x*-axis. Dotted lines represent theoretical DHS activity of the mixtures that were calculated using DHS activity and effector sensitivity measured from individual AthDHS enzymes. F, Absolute expression levels of *AthDHS1*, *AthDHS2*, and *AthDHS3* genes in six Arabidopsis tissues from eFP browser. Full expression maps are available in Supplemental Figure S9, C. G, Absolute copy numbers of *AthDHS1*, *AthDHS2*, and *AthDHS3* expressed in 3- and 4-day-old Arabidopsis Col-0 etiolated and de-etiolated seedlings, respectively. H, DHS activity assays of crude extracts isolated from de-etiolated young spinach seedlings in the presence of 1 mM AAAs, chorismate, and caffeate. **P ≤ 0.01, ***P ≤ 0.001, and ****P ≤ 0.0001 denote significant differences by one-way ANOVA against the corresponding “No effector” samples for (A), (B), and (H), or the “AthDHS1” or “AthDHS2” sample for (C) and (G). Data are means ± SEM (*n* = 3 or 4 replicated reactions). All the individual data points are shown as dots, except for (D), (E), and (F).

To evaluate if the results of the recombinant DHS enzymes can also be observed in plant tissue-derived DHS activity, total protein extracts were prepared from fully expanded mature leaves of Arabidopsis and spinach and subjected to DHS assays using different inhibitors. Chorismate and caffeate at 1 mM reduced the total DHS activity in both Arabidopsis and spinach leaf extracts by more than half (Figure 5, A and B), consistent with their inhibitory effects on all three AthDHSs (Figures 3, 4). The AAA mixture at 1 mM, by contrast, had no significant effects on the DHS activity of both Arabidopsis and spinach extracts (Figure 5, A and B). This observation is consistent with previous reports (Huisman and Kosuge, 1974; Pinto et al., 1986; Sharma et al., 1993) but contradicts the results of the recombinant AthDHS2 that was inhibited by the same AAA treatment (Figure 2, C).

Initially, we hypothesized that the *AthDHS2* gene expression may be very low compared with that of *AthDHS1* and *AthDHS3*, which encode DHS enzymes that are not inhibited by AAAs (Figure 2, C). To test this possibility, the copy numbers of different *AthDHSs* were compared by reverse transcription quantitative PCR (RT-qPCR) in mature leaves of Arabidopsis that were harvested at the same stage as for the above DHS activity assays. However, the level of the *AthDHS2* transcripts was still >50% of *AthDHS1* and roughly 7-fold more abundant than *AthDHS3* (Figure 5, C). In roots, *AthDHS1* and *AthDHS2* were expressed at similar levels, which are much higher than those of *AthDHS3* (Figure 5, C). These results indicate that the AAA-inhibited AthDHS2 is still expressed at substantial levels in mature tissues, even though overall DHS activity does not exhibit any inhibition by AAAs (Figure 5, A and B). Thus, an additional factor must be contributing to the lack of the observed AAA inhibition of DHS activity (Figure 5, A and B).

Next, we thought that the presence of non-inhibited DHS isoforms (e.g. AthDHS1) may affect the AAA sensitivity of AthDHS2, given that AthDHSs are known to function as tetrameric or dimeric forms (Suzich et al., 1985; Pinto et al., 1986; Webby et al., 2005). To test this, the recombinant enzymes of AAA non-inhibited AthDHS1 and AAA-inhibited AthDHS2 were mixed with different ratios (0, 0.25, 0.5, 0.75, and 1) and DHS activity assays were conducted with different inhibitors. Based on the kinetic parameters and the regulatory behaviors of individual AthDHSs, the expected level of DHS activity was first calculated and plotted (dotted lines in Figure 5, D, see the “Materials and methods” section). Without any inhibitors or with Phe, the observed DHS activity in the various AthDHS1 and AthDHS2 mixtures matched with the theoretical plot (black and purple lines, respectively, in Figure 5, D), consistent with the absence of inhibitory effects of Phe on both AthDHS1 and AthDHS2 (Figure 2, D and E). Notably, in the presence of 1 mM Tyr and Trp, however, observed DHS activity was significantly higher than theoretically calculated activities in any of the AthDHS1 and AthDHS2 mixtures (orange and magenta lines, respectively, in Figure 5, D). Similar results were obtained for the AthDHS2 and AthDHS3 mixture (Figure 5, E). These results suggest that the presence of AthDHS1 and AthDHS3 reduces the sensitivity of AthDHS2 to AAAs, which likely contributes to the observed lack of AAA-mediated inhibition of DHS activity detected from the leaf extracts (Figure 5, A and B).

Gene Ontology (GO) analyses of publicly available co-expression data suggest that *AthDHS2* is co-expressed with genes involved in plastid development and photosynthesis, whereas *AthDHS1* and *AthDHS3* are associated with other shikimate, AAA, and phenylpropanoid genes (Supplemental Figure S8 and Supplemental Table S1; Peltier et al., 2004; Ytterberg et al., 2006; Obayashi et al., 2018). Unlike *AthDHS1* and *AthDHS3*, which are strongly expressed in response to pathogens and elicitors, *AthDHS2* is often induced upon changes in light conditions, based on expression databases (Supplemental Figure S9; Hruz et al., 2008; Klepikova et al., 2016). Transcriptome data from eFP browser suggest that *AthDHS2* tends to be expressed predominantly in early developmental stages (Figure 5, F and Supplemental Figure S9, C). Also, DTT-independent DHS activity was detected only in AthDHS2 but not in AthDHS1 or AthDHS3 (Figure 2, A). Thus, we thought that *AthDHS2* may be highly expressed in young developing seedlings with low photosynthetic activity (and thus limited reducing energy; Winter et al., 2007). Our RT-qPCR analysis showed up to 10-fold higher expression of *AthDHS2* than *AthDHS1* or *AthDHS3* in 3–4-day-old etiolated and de-etiolated seedlings of Arabidopsis (Figure 5, G).

With the predominant expression of *AthDHS2* in photosynthetically less-active tissues, such as etiolated and de-etiolated seedlings, we then rationalized that AAA-mediated DHS inhibition may be detectable. Since Arabidopsis seedlings are too small to obtain enough enzyme extract for the DHS assay, we tested this hypothesis using de-etiolated whole seedlings of spinach, whose genome also contains an AthDHS2-like ortholog (Supplemental Figure S1). DHS activity from the spinach seedling extracts was significantly reduced in the presence of AAAs as well as chorismate and caffeate (Figure 5, H). Thus, plant tissue-derived DHS activity can be also inhibited by AAAs when AAA-sensitive AthDHS2 is predominantly expressed such as in young seedlings; however, when AthDHS2 expression is not predominant, such as in mature leaves, the AAA-sensitivity of the AthDHS2 activity is not observable not only due to its low expression (Figure 5, C) but also because the presence of AthDHS1 and AthDHS3 makes AthDHS2 insensitive to AAA (Figure 5, D and E).

### Arabidopsis *dhs1* and *dhs3* mutants are hypersensitive to Tyr and Trp, respectively

**Figure 6:**
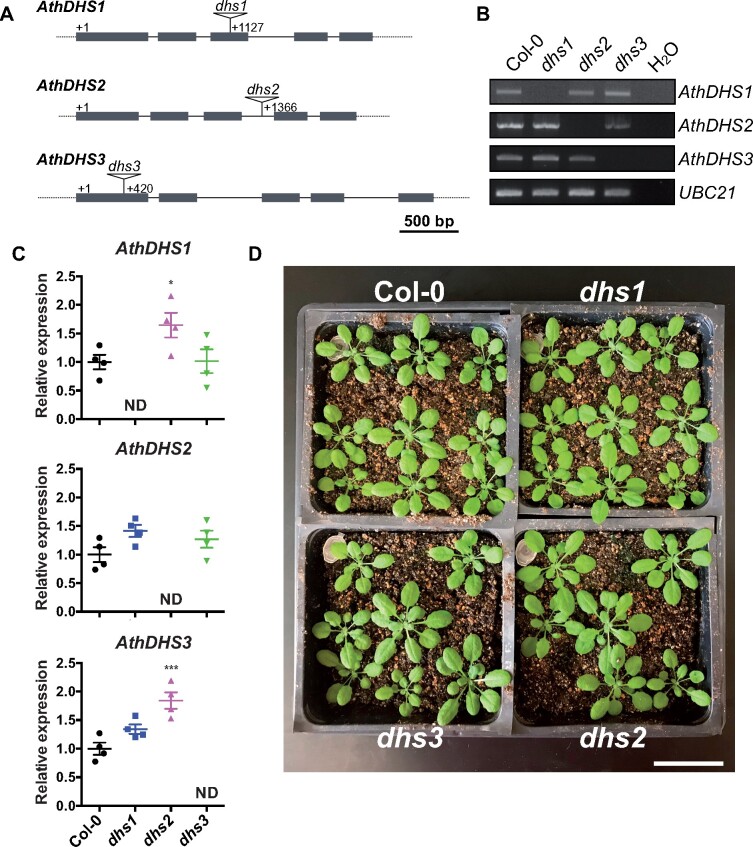
Knock-out mutants of *AthDHS* genes did not exhibited drastic phenotypes under standard growth condition. (A) Schematic structural models of *AthDHS1* (AT4G39980), *AthDHS2* (AT4G33510), and *AthDHS3* (AT1G22410) genes, with exons and introns shown in gray boxes and solid lines, respectively. The positions of the T-DNA insertion are indicated by triangles for *dhs1* (SALK_055360), *dhs2* (SALK_033389), and *dhs3* (SK2559). (B) RT-PCR analysis of *AthDHS1*, *AthDHS2*, and *AthDHS3* transcripts in Col-0 and the *dhs* mutants with UBC21 (AT5G25760) as a control. (C) RT-qPCR analysis of *AthDHS1*, *AthDHS2*, and *AthDHS3* gene expression in Col-0 and the *dhs* mutants. *P ≤ 0.05 and ***P ≤ 0.001 denote significant differences by one-way ANOVA against the corresponding Col-0 samples. Data are means ± SEM (*n* = 4 replicated samples). All the individual data points are shown as dots. ND: not detectable. (D) Growth picture of 4-week-old Col-0, *dhs1*, *dhs2*, and *dhs3* plants. Bar = 3 cm.

To further investigate *in planta* functions of the AthDHS isoforms, we obtained and characterized T-DNA insertional mutants of *AthDHS1*, *AthDHS2*, and *AthDHS3* (*dhs1*, *dhs2*, and *dhs3*, respectively) from *A. thaliana*. A T-DNA fragment was present in the third and first exons of the *dhs1* and *dhs3* mutants, respectively, and in the third intron of *dhs2* (Figure 6, A). Their corresponding transcripts were not detectable by RT-PCR and RT-qPCR analyses in these mutants (Figure 6, B and C). *AthDHS1* and *AthDHS3* gene expression was upregulated 1.5–2-fold in the *dhs2* mutant, but no significant change of *AthDHS* transcript levels were detected in *dhs1* or *dhs3* (Figure 6, C). No visual growth phenotype was observed for any of the *dhs* mutants under standard conditions used in this study (Figure 6, D).

**Figure 7:**
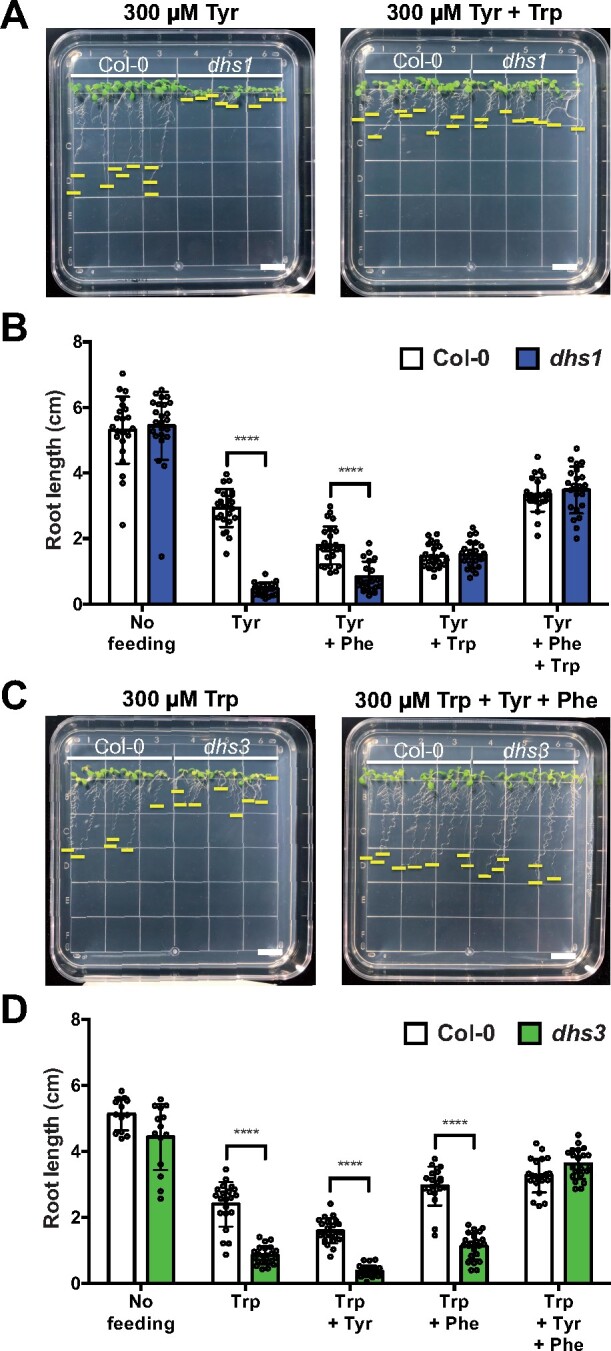
Root growth inhibition of *dhs1* and *dhs3* was attenuated by supplying additional AAAs. (A and B) Root growth pictures (A) and length measurement (B) of 10-day-old Col-0 and *dhs1* on media containing Tyr alone or combination of Tyr and Trp at 300 µM. (C and D) Root growth pictures (C) and length measurement (D) of 10-day-old Col-0 and *dhs3* on the media containing Trp alone or combination of all AAAs at 300 µM. Positions of their root tips are indicated by yellow lines. White scale bars = 1 cm. ****P ≤ 0.0001 denote significant differences by one-way ANOVA against the corresponding Col-0 samples. Data are means ± sd (*n* > 10 replicated sample). All the individual data points are shown as dots.

Given the observed isoform-specific regulation of the AthDHS2 enzyme by AAAs (Figure 2), the effects of AAA treatment on these *dhs* mutants were examined by growing them on growth media containing different concentrations of individual AAA. All of the *dhs* mutants grown without any AAAs were again indistinguishable from Col-0 wild type and had similar root lengths (Supplemental Figure S10). High concentrations of Phe beyond 300 µM suppressed the root growth of all *dhs* mutants but similarly to Col-0, with the IC_50_ values of ∼550 µM (Supplemental Figure S10, B). Notably, Tyr and Trp treatment at and beyond 100 µM specifically impaired the root growth of *dhs1* and *dhs3*, with IC_50_ values of 79.0 and 139.6 µM, respectively, whereas other genotypes showed 50% growth inhibition beyond ∼300 µM (Figure 7 and Supplemental Figure S10). It is important to note that the *dhs2* mutant showed similar responses to Col-0 under all of the AAA treatments (pink bars in Supplemental Figure S10, B). Also, none of the *dhs* mutants showed sensitivity to up to 1000 µM of shikimate (Supplemental Figure S10, B).

To test if the observed Tyr and Trp sensitivity of the *dhs1* and *dhs3* mutant, respectively, are indeed due to the loss of *AthDHS1* and *AthDHS3*, their wild-type CDS genes were introduced to the *dhs1* and *dhs3* mutant backgrounds using their native promoters (see the “Materials and methods” section). The *dhs1* and *dhs3* mutants carrying the respective *AthDHS1* and *AthDHS3* rescue construct (*dhs1 AthDHS1* and *dhs3 AthDHS3*) recovered the root growth phenotype under high Tyr and Trp conditions, respectively (Supplemental Figure S11). The *dhs1 AthDHS1* and *dhs3 AthDHS3* lines behave very similarly to Col-0. The introduction of the empty vector into the *dhs1* and *dhs3* mutants (*dhs1 Empty* and *dhs3 Empty*) did not result in growth recovery under high AAA conditions (Supplemental Figure S11). These results further support that the lack of *AthDHS1* and *AthDHS3*, but not an unknown secondary mutation(s), resulted in the hypersensitivity to Tyr and Trp in *dhs1* and *dhs3*, respectively.

To better understand the inhibitory effects of Tyr and Trp, we additionally supplied other AAAs and/or shikimate to the media containing Tyr or Trp at 300 µM. The addition of shikimate at 500 µM had no effect on the Tyr- and Trp-mediated growth inhibition of *dhs1* and *dhs3*, respectively (Supplemental Figure S12). Addition of 300 µM Trp with Tyr, however, resulted in similar root length between *dhs1* and Col-0 (Figure 7, A and B). Further addition of Phe, together with Trp, drastically recovered the stunted root growth of *dhs1* in the presence of Tyr, although Phe alone was not sufficient to exert such effect (Figure 7, A and B). The Trp sensitivity of *dhs3* was not recovered by the addition of Tyr or Phe individually; however, when both Tyr and Phe were added together, *dhs3* showed drastic recovery in root growth and exhibited the root length similar to Col-0 (Figure 7, C and D). The observed recovery of Tyr and Trp sensitivity of *dhs1* and *dhs3* by Phe together with Trp and Tyr, respectively, suggests that the depletion of other AAAs resulted in the inhibition of their root growth.

### Tyr sensitivity of Arabidopsis plants is prevented by lack of the Tyr-inhibited AthDHS2 enzyme

**Figure 8:**
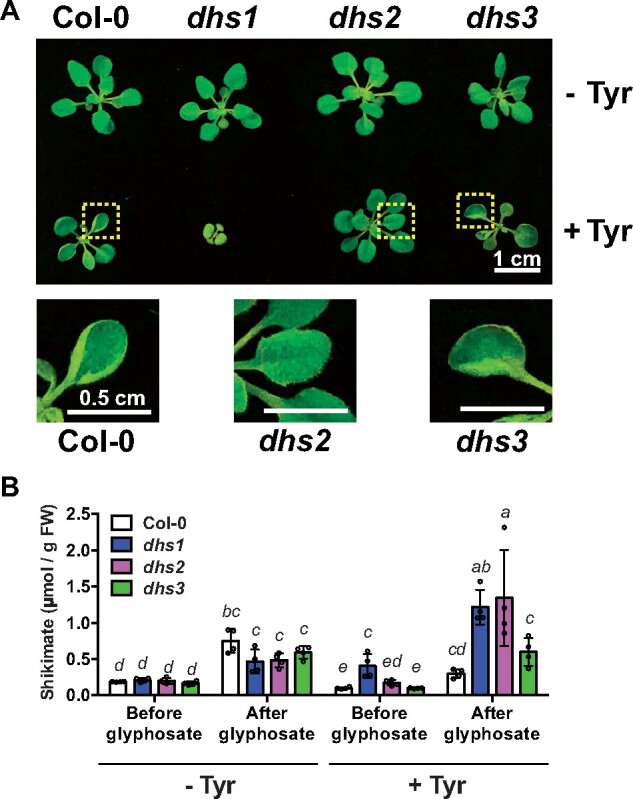
Shikimate accumulated to higher levels after glyphosate application in *dhs2* plants grown under high Tyr condition. (A) Plant growth picture of 14-day-old Col-0, *dhs1*, *dhs2*, and *dhs3* grown on media with and without Tyr at 300 µM. Magnified images of the leaves are shown below. White scale bars = 1 (above) or 0.5 cm (below). (B) Shikimate contents of Col-0, *dhs1*, *dhs2*, and *dhs3* grown on media with and without Tyr at 300 µM, before and 2 days after treatment of 250 µM glyphosate. Different letters indicate statistically significant differences between samples (two-way ANOVA, *P* < 0.05). Data are means ± SEM (*n* = 4 replicated samples). All the individual data points are shown as dots.

Unlike *dhs1* and *dhs3*, the *dhs2* mutant did not show a root phenotype under any of the AAA treatments (Supplemental Figure S10). Fourteen days after germination on 300 µM Tyr, newly growing leaves of Col-0 and *dhs3* plants exhibited a cup-like shape, which was not observed without the Tyr treatment (Figure 8, A and Supplemental Figure S13). Consistent with the root phenotype, the shoots of *dhs1* were very small and pale under this condition. Interestingly, the cup-shaped leaf phenotype did not appear in the *dhs2* mutant when grown side-by-side with other genotypes on the Tyr-containing media (Figure 8, A and Supplemental Figure S13). Introduction of the *AthDHS2* gene in the *dhs2* mutant background restored the phenotype of cup-shaped leaves (Supplemental Figure S14, A). Thus, the lack of AthDHS2, the only DHS isoform inhibited by Tyr (Figure 2), prevents the Tyr sensitivity of *A. thaliana* plants.

We then hypothesized that the Tyr treatment restricts carbon flow through the shikimate pathway in Arabidopsis, but not in the *dhs2* mutant that lacks Tyr-inhibited AthDHS2. To test this hypothesis, Col-0 and *dhs* mutants were treated with and without Tyr (at the same 300 µM as above) and/or glyphosate—an herbicide that inhibits 5-*enol*pyruvylshikimate-3-phosphate synthase (EPSPS) enzymes *in planta* (Figure 1) and hence promotes shikimate accumulation, which likely reflects the difference in carbon flow through the shikimate pathway (Hollander and Amrhein, 1980; Klee et al., 1987; Pollegioni et al., 2011). The glyphosate treatment without Tyr indeed increased shikimate levels as expected, but similarly between the genotypes (Figure 8, B, left). Feeding Tyr at 300 µM by itself (without glyphosate treatment, Figure 8, B, right) did not alter the shikimate level in all genotypes, except for the elevated shikimate content of *dhs1*, expressed in µmol/g FW, due to its extremely small shoot size (Figure 8, A). After the treatment with glyphosate, the *dhs2* plants grown with the exogenous Tyr showed five-fold higher accumulation of shikimate, while other genotypes exhibited only two–three-fold increase (Figure 8, B, right). Introducing the wild-type *AthDHS2* gene in the *dhs2* mutant almost completely eliminated the glyphosate-induced elevation of shikimate under high Tyr condition (Supplemental Figure S14, B). These results suggest that the Tyr treatment restricts shikimate production through the Tyr-mediated negative feedback inhibition of AthDHS2 enzymes in Arabidopsis plants.

To further test if the absence of AthDHS1 or AthDHS3 in *dhs1* or *dhs3* leads to AAA-mediated inhibition of DHS activity, which was not observed in Col-0 (Figure 5, A), DHS activity was analyzed from the crude extracts of 4-week-old leaves of Col-0 and *dhs* mutants in the presence of individual AAAs. However, DHS activity was not inhibited by any AAAs at 1 mM in any of the *dhs* mutants (Supplemental Figure S15). This is likely because the remaining AthDHS3 and AthDHS1 in *dhs1* and *dhs3*, respectively, is sufficient to mask the AthDHS2-mediated feedback inhibition of DHS activity by AAAs in the crude extract.

### High light-induced phenylpropanoid production is attenuated in *dhs* mutants

To further characterize *in vivo* functions of *AthDHSs*, the *dhs* mutants and Col-0 plants were subjected to metabolite analyses using gas chromatography–mass spectrometry and liquid chromatography–mass spectrometry (GC–MS and LC–MS, respectively), first using leaf tissues of 4-week-old plants grown under standard growth conditions. Compared with Col-0 the *dhs1* mutant accumulated less Phe, Asp, and Glu, while the *dhs3* mutant exhibited less Phe and Ala (Supplemental Table S2, A). The levels of the other amino acids including Tyr and Trp were not significantly different among all of the *dhs* mutants and Col-0 (Supplemental Table S2, A). The levels of AAA-derived specialized metabolites such as glucosinolates, flavonols, and tocopherols were comparable between Col-0 and the *dhs* mutants (Supplemental Table S2, A). These results suggest that the lack of individual *AthDHS* genes has minor impacts on overall plant phenotypes (Figure 6, D) and metabolite levels (Supplemental Table S2, A), with the exception of *dhs1* and *dhs3*-specific alteration in some amino acid levels.

Given that various stress conditions induce the production of AAA-derived natural products (Bohinc et al., 2012; Landi et al., 2015; Liu and Lu, 2016) and the expression of some *DHS* genes (Pinto et al., 1988; Dyer et al., 1989; Keith et al., 1991; Devoto et al., 2005; Yan et al., 2007), these *dhs* mutants were subjected to stress treatments and evaluated their phenotypic and metabolic responses. Prior studies showed that methyl-jasmonate (MeJA) induces *AthDHS1* expression (Winter et al., 2007) and promotes biosynthesis of Trp- and Trp-derived defense compounds such as glucosinolates (Kliebenstein, 2011; Bohinc et al., 2012; Sanchez-Pujante et al., 2017). However, no significant and consistent differences were observed between Col-0 and *dhs1* in the accumulation of these metabolites (Supplemental Figure S16).

**Figure 9:**
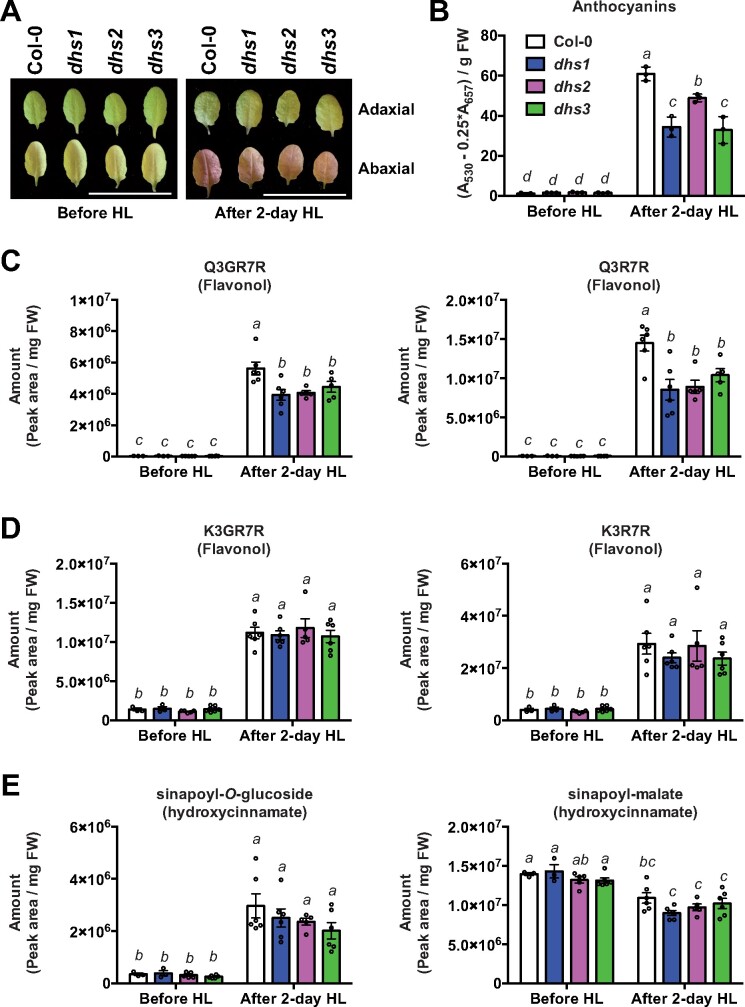
High light-induced phenylpropanoid production is attenuated in *dhs* mutants. (A) Leaf pictures of Col-0, *dhs1*, *dhs2*, and *dhs3* before and 2 days after treatment of continuous highlight (HL) treatment. Light intensity was changed from 100 to 650 µE. White scale bars = 3 cm. (B–E) Accumulation of anthocyanins (B), Q3GR7R and Q3R7R (C), K3GR7R and K3R7R (D) and sinapoyl-*O*-glucoside and sinapoyl-malate (E) in Col-0, *dhs1*, *dhs2*, and *dhs3* before and 2 days after treatment of continuous HL. The complete metabolite profiles before and 2 days after HL treatment are available in Supplemental Table S2. Different letters indicate statistically significant differences between samples (two-way ANOVA, *P* < 0.05). Data are means ± SEM (*n* = 4–6 replicated samples). All the individual data points are shown as dots. Q3GR7R, quercetin-3-*O*-(2″-*O*-rhamnosyl)glucoside-7-*O*-rhamnoside; Q3R7R, quercetin-3-*O*-rhamoside-7-*O*-rhamnoside; K3GR7R, kaempferol-3-*O*-(2″-*O*-rhamnosyl)glucoside-7-*O*-rhamnoside; K3R7R, keampferol-3-*O*-rhamnoside-7-*O*-rhamnoside.

High intensity light (HL) stress induces biosynthesis of Tyr-derived tocopherols and Phe-derived phenylpropanoid compounds, such as flavonols and anthocyanin pigments (Das et al., 2011; Landi et al., 2015). To investigate the roles of different AthDHS isoforms in elevated production of Tyr- and Phe-derived metabolites, Col-0 and the *dhs* mutants were grown at 100 µE for 4 weeks and then subjected to 650 µE of HL treatment (Figure 9, A). Overall, the levels of AAAs and many of their derivatives were elevated after 2 days of the HL treatment (Supplemental Table S2, B), with the exception of reduced Phe levels likely due to its rapid utilization for phenylpropanoid biosynthesis (Tohge et al., 2013; Chen et al., 2016). AAA levels were overall similar among genotypes after the 2-day HL treatment, except for slightly higher Tyr in *dhs1* than other genotypes (Supplemental Table S2, B).

The HL treatment rapidly induced Phe-derived anthocyanin pigments, which became visible after 2 days in the abaxial surfaces of leaves (Figure 9, A). However, this was less pronounced in *dhs* mutants, with *dhs1* and *dhs3* having significantly less anthocyanin levels than Col-0 after 2 days (Figure 9, B). This metabolic phenotype was repeatedly observed at 2 and 5 days of the HL treatment (Supplemental Figure S17) and was rescued by introducing the *AthDHS* gene into each corresponding *dhs* mutant (Supplemental Figure S18). Flavonol quercetin glycosides, such as quercetin-3-*O*-rhamnoside-7-*O*-rhamnoside (Q3R7R), were also induced strongly after the HL treatment, but accumulated significantly less in all three *dhs* mutants than in Col-0 (Figure 9, C and Supplemental Table S2, B). The levels of other flavonols, kaempferol glycosides, and a hydroxycinnamate, sinapoyl-*O*-glucoside, were also elevated but less pronounced than quercetin derivatives or anthocyanins, and were not significantly different among genotypes (Figure 9, D and E and Supplemental Table S2, B). The levels of another hydroxycinnamate, sinapoyl-malate, were slightly but significantly decreased after 2-day HL treatment, and showed no significant differences between genotypes (Figure 9, E, right and Supplemental Table S2, B). The levels of glucosinolates were overall similar among genotypes, except one aliphatic glucosinolate, 7-methylsulphinylheptyl-glucosinolate (7MTH), which was higher in *dhs1* and *dhs3* than Col-0 (Supplemental Table S2, B). The HL treatment increased the levels of Tyr-derived lipophilic antioxidants, alpha- and gamma-tocopherols, but similarly among all genotypes (Supplemental Table S2, B). These metabolic phenotypes of the *dhs* mutants demonstrate that AthDHSs play important roles in the elevated production of phenylpropanoid compounds, such as quercetin derivatives and anthocyanin pigments, under HL stress.

## Discussion

### Plant DHS enzymes exhibit high *K*_m_ toward E4P and have varied redox dependency

Unlike in microbes, limited information is available on the biochemical properties of plant DHS enzymes, which catalyze the entry step for biosynthesis of AAAs and numerous plant natural products (Figure 1). Kinetic analyses of the recombinant AthDHS enzymes showed approximately 10-fold higher *K*_m_ values for E4P than those for PEP (Table 1), consistent with activity data for partially purified DHS from spinach leaves (Doong et al., 1992). The *K*_m_ values of AthDHSs for PEP were similar to those of microbial DHSs that have equivalent *K*_m_ for E4P and PEP (Table 1; Liu et al., 2008; Cross et al., 2013; Cross and Parker, 2013; Reichau et al., 2016). On the other hand, the *K*_m_ of AthDHSs for E4P is 10-fold higher than those of microbial DHSs. Although detection of intracellular levels of E4P has not been successful from plant tissues (Arrivault et al., 2009), the *in vivo* E4P concentration is likely much higher in plant cells than in non-photosynthetic organisms due to the presence of the Calvin–Benson cycle (the reductive pentose phosphate pathway), which provides an additional source of E4P besides the oxidative pentose phosphate pathway. Thus, the availability of E4P likely has a significant impact on overall activity of DHS and hence the shikimate pathway *in planta*. A prior study showed that the suppression of transketolase, which is responsible for the E4P production in the pentose phosphate pathways, led to a major reduction in AAAs and phenylpropanoid compounds in tobacco (*Nicotiana tabacum*) plants (Henkes et al., 2001). Thus, an enhanced supply of E4P, such as by upregulating the pentose phosphate pathways, likely plays an important role in maintaining high carbon flux through the shikimate pathway in plants.

**Figure 10:**
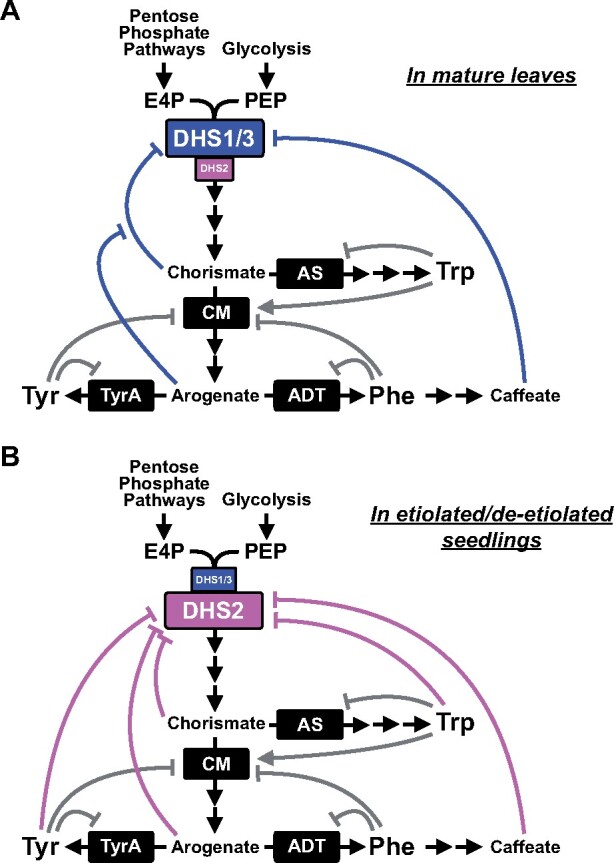
Working models for highly complex effector-mediated feedback systems of the shikimate and AAA pathways in different tissues of Arabidopsis. Different isoforms of plant DHS enzymes are subjected to highly complex effector-mediated feedback regulation in a tissue-specific manner. (A) In mature leave tissues, total DHS activity is mainly governed by AthDHS1 and AthDHS3 enzymes, which are not directly inhibited by AAAs. Chorismate strongly inhibits AthDHS1 and AthDHS3, which is offset by arogenate. (B) In young etiolated/de-etiolated seedlings, the AAA-inhibited AthDHS2 isoform is dominantly expressed and directly inhibited by Tyr and Trp. Chorismate and arogenate also inhibit AthDHS2. In both tissues, Phe has no regulatory effects on plant DHSs but caffeate and its derivatives act as strong inhibitors for all AthDHS isoforms, likely coordinating the shikimate and Phe phenylpropanoid biosynthesis. Blue and pink lines represent feedback inhibition of AthDHS1/3 and AthDHS2 enzymes, respectively, by the indicated effectors. Gray lines with an arrowhead and hash indicate known feedback activation and inhibition, respectively, of AS, CM, TyrA, and ADT enzymes. DHS, 3-deoxy-D-*arabino-* heptulosonate 7-phosphate synthase; AS, anthranilate synthase; CM, chorismate mutase; TyrA, arogenate dehydrogenase; ADT, arogenate dehydratase; E4P, D-erythrose 4-phosphate; PEP, phospho*enol*pyruvate.

All three AthDHSs were found to be redox-dependent (Figure 2, A), which was also observed in a prior study for AthDHS1 (Entus et al., 2002). Although the underlying mechanism remains to be determined, the redox regulation of plant DHS enzymes can contribute to the functional coupling of the pentose phosphate and shikimate pathways (Mousdale and Coggins, 1985; Ganson et al., 1986; Maeda and Dudareva, 2012). Notably, however, residual but substantial levels of redox-independent DHS activity was detected, but only in AthDHS2 (Figure 2, A), which belongs to a distinct phylogenetic clade from AthDHS1 and AthDHS3 (Supplemental Figure S1). Actively growing young tissues, for example, have developing plastids with limited photosynthetic activity, but have a high demand for AAAs, along with other amino acids, to support rapid growth (Pyke, 1999; Jarvis and López-Juez, 2013; Hildebrandt et al., 2015). Under such conditions, the unique redox-independent AthDHS2 activity may allow Arabidopsis plants to maintain the basal levels of the shikimate pathway activity without redox activation (Figure 10, B).

### The DHS2 enzyme is inhibited by Tyr and Trp in Arabidopsis

Microbial DHS enzymes are directly feedback inhibited by AAAs, which tightly controls the entry step of AAA biosynthesis to maintain cellular amino acid and metabolic homeostasis (Bentley, 1990). By contrast, previous studies found that plant DHS activities are not inhibited by AAAs (Huisman and Kosuge, 1974; Pinto et al., 1986; Sharma et al., 1993); this has long puzzled plant biochemists seeking to understand how the shikimate pathway is regulated in plants (Herrmann, 1995; Tzin and Galili, 2010; Maeda and Dudareva, 2012; Lynch and Dudareva, 2020). Our initial assays of mature leaf tissues of Arabidopsis and spinach also failed to detect AAA inhibition (Figure 5, A and B). Surprisingly, however, we found that Tyr and Trp inhibit recombinant AthDHS2, but not AthDHS1 and AthDHS3 (Figure 2), which was observed many times in independent experiments (e.g. Figure 3, A and C).

Further analyses revealed at least two factors that may explain why AAA-mediated inhibition of DHS activity was rarely detected from plant tissue extracts. First, the expression levels of AAA-inhibited AthDHS2 was the same as or lower than that of AAA non-inhibited AthDHS1 in mature leaf and root tissues (Figure 5, C), where the majority of DHS activity has been analyzed (Huisman and Kosuge, 1974; Pinto et al., 1986; Sharma et al., 1993). Second, the AAA sensitivity of AthDHS2 was attenuated when AthDHS2 was mixed with AthDHS1 or AthDHS3 (Figure 5, D and E), which are not inhibited by AAAs (Figure 2, C and D). A similar case of one enzyme affecting a property of another isoform was also previously found in poplar (*Populus trichocarpa*) 4-coumaric acid:CoA ligase 5 (4CL5) in monolignol biosynthesis that alters substrate specificity of another isoform 4CL3 through heterocomplex formation (Chen et al., 2014). Although the molecular mechanism behind this intriguing observation requires further investigation, when AAA non-inhibited AthDHS1 is predominantly expressed, such as in mature leaves, AthDHS1 masks the AAA-mediated inhibition of AthDHS2 (Figure 10, A). By contrast, in young seedlings where AthDHS2 was predominantly expressed, we can detect AAA-mediated inhibition of DHS activity (Figure 10, B).

This study further showed that the *dhs1* and *dhs3* mutants are sensitive to Tyr and Trp, respectively (Figure 7 and Supplemental Figure S10), the former was also noted in a prior thesis study (Crowley, 2006). Further, we were able to fully rescue these phenotypes by the introduction of the corresponding wild-type *AthDHS* genes (Supplemental Figure S11). Although we do not know why *dhs1* and *dhs3* are specifically sensitive to Tyr and Trp, respectively, these findings provide *in vivo* evidence that the lack of AthDHS1 or AthDHS3 increases AthDHS2 sensitivity to Tyr and Trp inhibition, respectively, *in planta*. Conversely, the *dhs2* mutant was resistant to Tyr (Figure 8).

Prior and current studies showed that Arabidopsis is sensitive to AAAs, especially to high Tyr concentration (Voll et al., 2004; de Oliveira et al., 2019), and exhibits a cup-shaped leaf phenotype (Figure 8, A and Supplemental Figure S13). A similar cup-shaped phenotype was observed when the feedback-insensitive prephenate or arogenate dehydratase was expressed in Arabidopsis plants, elevating the levels of Phe (Tzin et al., 2009; Huang et al., 2010) . The *dhs2* mutant, however, did not show the cup-shaped leaves in the presence of Tyr (Figure 8, A and Supplemental Figure S13), suggesting that the presence of the Tyr-inhibited AthDHS2 is involved in the Tyr sensitivity phenotype of Arabidopsis plants. The additional supply of glyphosate, which blocks the shikimate pathway flux by inhibiting EPSPS (Hollander and Amrhein, 1980; Pollegioni et al., 2011), led to significantly elevated shikimate accumulation in Tyr-treated *dhs2* compared with Col-0 (Figure 8, B). These *in vitro* and *in vivo* data together demonstrate that the AthDHS2 enzyme is indeed inhibited by Tyr and Trp in Arabidopsis plants.

### Roles of different AthDHS enzymes in Arabidopsis

Why do AAAs inhibit one DHS isoform (i.e. AthDHS2) but not the others in plants? Similar to microbes, in young seedlings, where *AthDHS2* is predominantly expressed (Figure 5, G), AAAs are produced mainly for the synthesis of proteins required for growth. Therefore, the unique AAA-mediated regulation of AthDHS2 (Figure 2, C and E) likely plays a critical role in controlling how much carbon flows into the shikimate pathway in these growing tissues (Figure 10, B) so that other amino acids and primary metabolites will not be depleted (Bentley, 1990;  Herrmann, 1995; Tzin and Galili, 2010; Maeda and Dudareva, 2012). It is important to note that the inhibition of AthDHS2 by Tyr and Trp occurred at the IC_50_ values of low 200 µM ranges (Figure 2, F), which were actually similar to the IC_50_ of the growth inhibition of Arabidopsis seedlings by Tyr and Trp (Supplemental Figure S10, B). By contrast, the IC_50_ (or *K*_i_) values of downstream TyrA and AS enzymes are ∼4–10-fold lower than those of AthDHS2 (Li and Last, 1996; Rippert and Matringe, 2002b; Kanno et al., 2004; Schenck et al., 2015; Schenck et al., 2017; Lopez-Nieves et al., 2018). This suggests that when cellular concentrations of Tyr and Trp are increased *in planta*, TyrA and AS activities will be initially inhibited before AthDHS2. The feedback regulation of AthDHS2 by Tyr and Trp may then act as a second layer of regulation to ensure that excess carbon will not flow into the shikimate pathway. High levels of free amino acids can accumulate transiently during developmental transitions, though difficult to detect, and are observed during senescence, when proteins are actively degraded (Soudry et al., 2005; Hirota et al., 2018) and *AthDHS2* is highly expressed (Supplemental Figure S9). Thus, the regulation of AthDHS2 likely allows tight control of overall AAA levels and metabolic homeostasis under certain developmental conditions such as early seedling growth and senescence.

In contrast to AthDHS2, AthDHS1 and AthDHS3 were not inhibited and were even slightly activated by AAAs (Figure 2). *AthDHS1* was the major *DHS* gene to be expressed in roots and mature leaves of Arabidopsis (Figure 5, C). *AthDHS1* and *AthDHS3* are co-expressed with genes involved in AAA biosynthesis as well as AAA-derived compounds, such as Trp and Phe-derived specialized metabolites, respectively (Supplemental Figure S8 and Supplemental Table S1, Obayashi et al., 2018). Also, *AthDHS1* and *AthDHS3* are strongly induced upon various biotic and abiotic stresses based on Arabidopsis expression databases (Supplemental Figure S9), which is consistent with orthologs of *AthDHS1* and *AthDHS3*, but not of *AthDHS2*, from different plants that are also responsive to various stresses (Pinto et al., 1988; Dyer et al., 1989; Keith et al., 1991; Devoto et al., 2005; Yan et al., 2007). While the induction of Trp- and Phe-derived compounds were largely unaltered in *dhs1* upon MeJA treatment at different concentrations (Supplemental Figure S16), HL-induced accumulation of Phe-derived anthocyanin pigments was substantially reduced in *dhs1* and *dhs3* and to a lesser extent in *dhs2* (Figure 9, B and Supplemental Figure S17). HL-induced production of flavonol quercetin derivatives was also significantly lower in all *dhs* mutants than Col-0 (Figure 9, C). Other phenylpropanoids, such as kaempferol and sinapoyl derivatives, were not significantly different between genotypes, likely due to their limited induction after the HL stress (Figure 9, D and E). Therefore, in mature leaves, especially under stresses, AthDHS1 and AthDHS3 likely allow rapid induction of total DHS activity without being inhibited by AAAs and by masking the AthDHS2 sensitivity to AAAs, together leading to efficient induction of phenylpropanoid compounds derived from Phe (Figure 10, A). Taken together, these data show that the three DHS isoforms have some distinct roles based on their mutant phenotypes, expression profiles, and distinct biochemical properties; however, these DHS enzymes also have overlapping roles and their combinatorial effects likely fine tune the regulation of the key entry step of this shikimate and AAA pathways under different conditions.

### The shikimate/AAA pathways are regulated by multiple metabolite-mediated feedback regulatory mechanisms in Arabidopsis

Amino acid biosynthetic pathways are typically feedback inhibited by the pathway end-products (i.e. amino acids, Galili et al., 2016). This is the case in the post-chorismate AAA pathways in both plants and microbes (Figure 1; Bentley, 1990; Tzin and Galili, 2010; Maeda and Dudareva, 2012) and in the upstream shikimate pathway in microbes (Bentley, 1990; Shumilin et al., 1999; Hartmann et al., 2003; Webby et al., 2010). This study, however, revealed that pathway intermediates are also potentially involved in regulating the plant shikimate and AAA pathways, at least in Arabidopsis (Figure 10): Chorismate is a strong inhibitor of all three AthDHSs (with IC_50_ of ∼50–100 µM, Figure 3, A and B), which is counteracted by arogenate (with IC_50_ of ∼300–350 µM,) in AthDHS1 and AthDHS3 (Figure 3, C and D). In bacteria, chorismate inhibits DHSs of *Pseudomonas aeruginosa* but much more weakly (with *K*_i_ of ∼1–2 mM) than Tyr or Trp (*K*_i_ of ∼5–40 µM; Whitaker et al., 1982). Although there is no report on *in planta* concentration of chorismate, the range of *K*_m_ values of Arabidopsis CM enzymes (150 µM to 1 mM; Westfall et al., 2014) is much higher than the IC_50_ values of AthDHSs for chorismate (∼50–100 µM, Figure 3, B). Thus, the observed inhibition of AthDHSs by chorismate likely plays a critical role *in planta*.

Importantly, chorismate and arogenate are located at the branch points into each AAA biosynthesis pathway (Figure 1). Since two of the chorismate-utilizing enzymes, AS and CM, are feedback inhibited by Trp and Phe or Tyr, respectively, in both plants (Romero et al., 1995a, 1995b) and microbes (Bentley, 1990), elevated levels of all AAAs will lead to chorismate accumulation and thus inhibition of all DHS enzymes. This may not be the case when individual AAAs (e.g. only Trp) accumulate, because the carbon flow can be redirected to the other AAA biosynthesis without accumulating chorismate. Therefore, the “sequential inhibitory” mechanism, as previously proposed (Doong et al., 1993), may allow plants to accumulate substantial levels of individual AAAs without completely inhibiting the initial step of the shikimate pathway (Figure 10). Such complex regulation of DHSs, together with their transcriptional regulation (Pinto et al., 1988; Dyer et al., 1989; Keith et al., 1991; Devoto et al., 2005; Yan et al., 2007), is likely important for fine tuning the stoichiometry of AAAs and efficiently producing certain AAA-derived compounds in specific tissues and conditions in plants.

The effect of arogenate on different AthDHS isoforms is much more difficult to decipher. In plants, Phe and Tyr inhibit ADT and TyrA enzymes, respectively, much more efficiently (IC_50_ of ∼10–60 µM; Connelly and Conn, 1986; Siehl and Conn, 1988; Rippert and Matringe, 2002a; Yamada et al., 2008) than the upstream CM enzymes (IC_50_ of ∼300 µM to 1 mM; Kuroki and Conn, 1988; Benesova and Bode, 1992). Therefore, theoretically speaking, arogenate accumulates only when both Phe and Tyr accumulate at the range of ∼60–300 µM that inhibit ADT and TyrA but not CM; beyond 300 µM, CM is inhibited and arogenate will not accumulate. In tissues predominantly expressing AthDHS1 and AthDHS3 (e.g. mature leaves, Figure 5, B), the accumulated arogenate offsets chorismate-mediated inhibition of AthDHS1 and AthDHS3 (Figure 3, C and D), likely allowing high chorismate accumulation and hence the production of chorismate-derived compounds such as folate, salicylic acid, indole alkaloids, and glucosinolates (Radwanski and Last, 1995; Kliebenstein, 2011; Saini et al., 2013; Sanchez-Pujante et al., 2017; Rekhter et al., 2019). However, when Tyr and Phe accumulate further (beyond ∼300 µM) and start to inhibit CM, the arogenate accumulation will be attenuated and hence chorismate will again inhibit DHSs. Unfortunately, the cost and instability of chorismate and arogenate did not allow direct testing of their effects on DHSs and the shikimate pathway *in planta*, and further genetic studies are needed to decipher their regulatory functions.

Although Phe is an important inhibitor of many microbial DHS enzymes (Bentley, 1990; Hartmann et al., 2003; Webby et al., 2010), Phe had no effect on any of AthDHS activity (Figure 2, D and E) consistent with prior studies (Huisman and Kosuge, 1974; Pinto et al., 1986; Sharma et al., 1993). Since plants synthesize high levels of Phe-derived phenylpropanoids, such as tannins and lignin (Boerjan et al., 2010; Vogt, 2010; Barbehenn and Peter Constabel, 2011), plants may sometimes need to accumulate high levels of Phe, and DHS activity should not be strictly inhibited by Phe. This study discovered that caffeate and its derivative, caffeoyl-shikimate, key intermediates of phenylpropanoid and monolignol biosynthesis (Boerjan et al., 2010; Bonawitz and Chapple, 2010; Vogt, 2010; Ragauskas et al., 2014), have very strong inhibitory effects on all AthDHSs with IC_50_ values of ∼50–70 µM (Figure 3, D). Since *p*-coumarate and ferulate lack the hydroxyl group and have *O*-methyl group, respectively, at the *meta*-position of their aromatic ring (Figure 4, A), the *meta*-hydroxyl group of caffeate is likely important for DHS inhibition, though the *p*-coumaroyl shikimate result suggests more complex interactions (Figure 4, C). Although further *in vivo* analyses are needed, the current results generate an interesting hypothesis that plant DHSs may monitor the levels of downstream intermediates, such as caffeate, rather than Phe, so as to directly coordinate the regulation of the shikimate and phenylpropanoid pathways (Figure 10).

### Conclusions and future perspectives

This study revealed that plant DHS activity and hence the shikimate pathway are subjected to highly complex regulation that is mediated by multiple pathway products and intermediates (Figure 10). This is in contrast to the more straightforward regulation of microbial DHSs, which are feedback regulated by AAAs (Bentley, 1990). This radical difference between plants and microbes is likely linked to their distinct demand and usage of AAAs: in most microbes AAAs are the pathway “end products” to be mainly utilized for protein synthesis (Bentley, 1990; Shumilin et al., 1999; Hartmann et al., 2003; Webby et al., 2010), whereas plants additionally produce numerous natural products derived from the shikimate and AAA pathways (Herrmann, 1995; Maeda and Dudareva, 2012). Diverse and often abundant phenylpropanoid compounds arguably played pivotal roles during the plant evolution, such as UV-absorbing phenolic compounds during plant colonization of land (Kay et al., 2017) and the principal cell wall component lignin in vascular plants (Boerjan et al., 2010; Bonawitz and Chapple, 2010; Ragauskas et al., 2014). To support the unique capability of synthesizing these diverse AAA-derived natural products, plant DHS enzymes likely function as critical gatekeepers to integrate various metabolic signals and control the carbon allocation for the production of these diverse aromatic compounds in plants.

While this study focused on the model plant *A. thaliana*, the regulatory mechanisms of DHS enzymes may vary in different plants, considering the tremendous diversity of downstream natural products that are synthesized in various plants. Since orthologs of AthDHS2, but not of AthDHS1, are absent in some plants (e.g. Solanaceae and Salicaceae, Supplemental Figure S1), it will be interesting to test if and how AAAs regulate any of the DHS isoforms in these plants without DHS2 orthologs. The current study laid the foundation for further molecular, genetic, and evolutionary analyses of the regulation of the shikimate pathway in different plants, tissues, developmental stages, and environmental conditions. Untangling the complex regulatory mechanisms of the shikimate pathway will eventually allow us to control the carbon flux into and through the shikimate pathway for plant-based production of various aromatic compounds critical to both plants and humans.

## Materials and methods

### Plant materials

Wild-type *A. thaliana* (Col-0) was grown under a 12/12-h 100 µE light/dark cycle with 85% air humidity in soil supplied with Hoagland solution or on the agarose-containing 0.5-strength Murashige and Skoog (MS) medium with 1% sucrose unless stated otherwise. T-DNA insertional mutants of *AthDHS1*, *AthDHS2*, and *AthDHS3* (SALK_055360, SALK_033389, and SK2559, respectively) were obtained from the Arabidopsis Biological Resource Center (ABRC). Their homozygous T-DNA insertions were confirmed by PCR using primers listed in Supplemental Table S3.

### Preparation of AthDHS protein expression vectors

For expression of AthDHS1, AthDHS2, and AthDHS3 enzymes in *E. coli*, the CDS fragments without sequences corresponding to their transit peptides (AthDHS1; residues 49–525, AthDHS2; residues 34–507, AthDHS3; residues 52–527) were amplified from cDNA by Phusion DNA polymerase (Thermo Fisher Scientific, Waltham, MA, USA) using primers listed in Supplemental Table S3. The PCR products were purified using the QIAquick Gel Extraction Kit (Qiagen, Hilden, Germany) and inserted into the NdeI–EcoRI sites for AthDHS1 and AthDHS3 or the NdeI–BamHI sites for AthDHS2 of the pET28a vector (Millipore-Sigma) using the In-Fusion HD cloning kit and protocol (Clontech, Mountain View, CA, USA). All of the resulting plasmids were sequenced to confirm that no errors were introduced during PCR and cloning.

### Recombinant protein expression and purification

*E. coli* Rosetta2 (DE3) cells (Millipore-Sigma) transformed with the pET28a vectors carrying individual *AthDHS* genes were first precultured in 10 mL LB media containing 50 µg/mL Kanamycin at 37°C overnight, and transferred to 500 mL LB media with Kanamycin to grow until the OD_600_ reached 0.3, when the temperature of the incubator was changed to 18°C. After adding isopropyl β-d-1-thiogalactopyranoside (IPTG, 0.1 mM final concentration) and ethanol (3% v/v final concentration) to induce expression of recombinant protein and to increase solubility (Chhetri et al., 2015), respectively, cells were grown overnight again. Bacteria were then harvested by centrifugation at 10,000 × *g* for 10 min, washed with 100 mL of 0.9 M NaCl, and resuspended in 15 mL of lysis buffer (20 mM phosphate pH 7.8, 100 mM NaCl, 1 mM DTT, and 10% glycerol). After sonication with 25 mg of lysozyme (Dot Scientific, Burton, USA) for 5 min in cold room, insoluble fraction was pelleted by centrifugation for 30 min at 50,000 × *g* to inject the supernatant into a 1-mL HisTrap FF column for purification of the His-tagged recombinant protein using an ÄKTA FPLC system (GE Healthcare, Chicago, IL, USA). The bound proteins were washed with 20 column volumes of 95% buffer A (0.5 M NaCl, 0.2 M sodium phosphate, and 20 mM imidazole) and 5% buffer B (0.5 M NaCl, 0.2 M sodium phosphate, and 1 M imidazole) and then with 90% buffer A and 10% buffer B, and eluted by 100% buffer B. The resulting fraction containing His-tagged DHS proteins was desalted by Sephadex G50 column size-exclusion chromatography (GE Healthcare) into 1 mL of 50 mM HEPES buffer (pH 8.0) and quantified by the Bradford assay (Bradford, 1976). The purified proteins were also analyzed by SDS–PAGE to evaluate their purity. All the purification steps were performed at 4°C unless stated otherwise.

### Preparation of crude protein extract from spinach and Arabidopsis

Spinach (*S. oleracea*) leaves were purchased at a local grocery store. For de-etiolated spinach seedlings, spinach was germinated on the soil and grown in the dark for 3 days, followed by exposure to normal growth light for 1 day for de-etiolation. More than 20 g of fully expanded mature leaves or whole de-etiolated seedlings were harvested and ground to a fine powder in a mortar and pestle with liquid nitrogen. After dissolving in 100 mL extraction buffer (20 mM HEPES [pH 7.6], 1 mM DTT and 0.1% β-mercaptoethanol) and filtrating with Miracloth, the samples were centrifuged at 10,000 × *g* for 10 min and subsequently at 50,000 × *g* for 30 min. The resulting supernatant was concentrated with Amicon Ultra Centrifugal Filters (Millipore-Sigma) by centrifugation until the solution volume became less than 1 mL. The concentrated solution was desalted twice by Sephadex G50 column size-exclusion chromatography (GE Healthcare) into 1 mL of 50 mM HEPES (pH 7.4) and quantified by the Bradford assay (Bradford, 1976). All the purification steps were performed at 4°C.

### Enzymatic assays

Unless otherwise noted, DHS enzymatic activity was monitored as previously described (Liu et al., 2008), with several optimizations for high-throughput assays with eight-strip PCR tubes and a thermal cycler. The enzyme solution (7.7 µL) containing 50 mM HEPES (pH 7.4) was preincubated with an effector molecule(s) at room temperature for 10 min. For assays using recombinant protein and enzyme fraction isolated from plant leaves, 0.01–0.05 µg and approximately 50 µg of proteins were used, respectively. After adding 0.5 µL of 0.1 M DTT, the samples were further incubated at room temperature for 15 min. During these incubations, the substrate solution containing 50 mM HEPES pH 7.4, 2 mM MnCl_2_, 4 mM E4P, and 4 mM PEP at final concentration was preheated at 37°C. The enzyme reaction was started by adding 6.8 µL of the substrate solution, then incubated at 37°C for 20 min, and terminated by adding 30 µL of 0.6 M trichloroacetic acid. After brief centrifugation, 5 µL of 200 mM NaIO_4_ (sodium meta-periodate) in 9 N H_3_PO_4_ was added to oxidize the enzymatic product and incubate at 25°C for 20 min. To stop the oxidation reaction, 20 µL of 0.75 M NaAsO_2_ (sodium arsenite), which was dissolved in 0.5 M Na_2_SO_4_ and 0.05 M H_2_SO_4_, was immediately mixed. After 5 min of incubation at room temperature, one-third of the sample solution was transferred to a new tube to be mixed with 50 µL of 40 mM thiobarbituric acid and incubated at 99°C for 15 min in a thermal cycler. Developed pink chromophore was extracted by putting the final solutions with 600 µL of cyclohexanone in eight-strip solvent-resistant plastic tubes, mixing vigorously and centrifuging them at 4,500 × *g* for 3 min to separate water- and cyclohexanone-based layers. The absorbance of the pink supernatant was read at 549 nm with the microplate reader (Infinite 200 PRO, TECAN, Männedorf, Switzerland) to calculate DAHP production with the molar extinction coefficient at 549 nm (*ε* = 549 nm) of 4.5 × 10^4^/M/cm. Reaction mixtures with boiled enzyme or without any substrates were run in parallel and used as negative controls to estimate the background signal for recombinant enzymes or enzymatic fraction from plant tissues, respectively.

For determination of kinetic parameters, the data of enzymatic activities measured in the presence of various concentration of substrates were calculated with the Michaelis–Menten equation in Graphpad Prism 6 (GraphPad Software, San Diego, CA, USA). Since AthDHS2 showed substrate inhibition by E4P, theoretical levels of AthDHS2 activity were estimated based on fitting with its Lineweaver–Burk plot and used to determine *V*_max_ and *K*_m_ values of AthDHS2 for E4P, as previously performed (Bernstein et al., 1978; Maeda et al., 2011).

### Gene expression analyses

Isolated total RNA was treated with DNaseI (Thermo Fisher Scientific) and reverse transcribed to synthesize cDNA with M-MuLV reverse transcriptase and random hexamer primers (Promega, Madison, USA). For RT-PCR analysis, 4-week-old soil-grown plants were used. RT-PCR was conducted using EconoTaq DNA Polymerase (Lucigen, Middleton, WI, USA) and primer pairs listed in Supplemental Table S3, following standard procedures. For RT-qPCR analysis of leaves and roots, 4-week-old soil-grown or 10-day-old agarose-plate-grown Arabidopsis Col-0 plants were used, respectively. For analysis of gene expression in etiolated or de-etiolated seedlings, Arabidopsis Col-0 plants were germinated on wet tissues in plastic plates and grown in the dark for 3 days. For de-etiolation, the etiolated plants were exposed to normal growth light (∼100 µE) for an additional 1 day. The 3- and 4-day-old etiolated and de-etiolated plants, respectively, were harvested to isolate RNA. RT-qPCR was conducted by the Stratagene Mx3000P (Agilent Technologies, Santa Clara, CA, USA) using GoTaq qPCR Master Mix (Promega) and target gene-specific primers listed in Supplemental Table S3. Four biological replicates with two technical RT-qPCR replicates were conducted. Expression of *UBC21* gene (AT4G27960) was used to normalize the sample-to-sample variations between different cDNA preparations.

In order to compare expression levels of different AthDHS isoforms, the copy numbers of individual *AthDHS* transcripts were determined by an absolute quantification method (Larionov et al., 2005). Briefly, the *AthDHS* coding regions were PCR amplified using the pET28 vectors carrying *DHS* sequences as the template, purified using the QIAquick Gel Extraction Kit (Qiagen), and quantified using Nanodrop (Thermo Fisher Scientific). The purified PCR fragments of known concentrations were then used to generate standard curves for each gene-specific primers, which was then used to calculate actual copy numbers of each *AthDHS* isoform (Larionov et al., 2005). For comparison of gene expression between Col-0 and the *dhs* mutants, dilution series of the Col-0 cDNA was used to determine the standard curves.

### Metabolite analysis

Approximately 50–80 mg of fully expanded mature leaves were pooled from multiple plants at the same developmental stages and ground in 800 µL of extraction buffer (v/v 2:1 of methanol and chloroform with 2 µg/mL isovitexin [Millipore-Sigma], 100 µM norvaline [Thermo Fisher Scientific], and 1.25 µg/mL Tocol [Matreya LLC, State College, PA, USA] for internal standards for soluble metabolite analysis by LC–MS and GC–MS and tocopherol analysis by GC–MS, respectively), using 1600 MiniG Tissue Homogenizer (SPEX SamplePrep, Metuchen, USA) and 3-mm glass beads. After adding 600 µL of H_2_O and then 250 µL of chloroform, polar phase containing amino acids, and non‐polar phase containing tocopherols were separated by centrifugation, dried in new tubes. To detect Trp and AAA-derived metabolites, LC–MS analysis was carried out as previous described with some modification (Alseekh et al., 2015). The dried metabolites were resuspended in 70 µL of 80% LC–MS-grade methanol. Two microliters of the sample was injected onto a HSS T3 C18 reversed phase column (100 × 2.1 mm i.d., 1.8-μm particle size; Waters, Milford, CT, USA) and eluted using a 20-min gradient comprising 0.1% formic acid in LC–MS-grade water (solvent A) and 0.1% formic acid in LC–MS-grade acetonitrile (solvent B) at a flow rate of 0.4 mL/min and column temperature of 40°C. The binary linear gradient with following ratios of solvent B was used: 0–1 min, 1%; 1–13 min, 1%–35%; 13–14.5 min, 35%–70%; 14.5–15.5 min, 70%–99%, 15.5–17 min, 99%; 17–17.5 min, 99%–10%; 17.5–20 min, 1%. The spectra were recorded using full scan mode of negative ion detection, covering a mass range from *m*/*z* 100 to 1,500. The resolution was set to 25,000 and the maximum scan time was set to 250 ms. The sheath gas was set to a value of 60, while the auxiliary gas was set to 35. The transfer capillary temperature was set to 150°C, while the heater temperature was adjusted to 300°C. The spray voltage was fixed at 3 kV, with a capillary voltage and a skimmer voltage of 25 and 15 V, respectively. Retention times, MS spectra, and associated peak intensities were extracted from the raw files using the Xcalibur software (Thermo Fisher Scientific). For confirmation of the identity of almost all compounds, LC–MS/MS analysis was performed with normalized collision energy (NCE) 20%, observing the fragmentation patterns (Supplemental Table S4). The identity of Trp and I3M peaks was confirmed by comparing their accurate masses and retention times with those of the corresponding authentic standards. Quantification of the other amino acids (e.g. Tyr and Phe) was conducted by GC–MS as previously performed (Wang et al., 2017; de Oliveira et al., 2019). Tocopherol analysis was also carried out by GC–MS as previously described (Wang et al., 2019). For anthocyanin quantification, the polar phase isolated for amino acid analysis was diluted 10 times with water in a new tube. After adding 5 µL of 5 N HCl for acidification, the absorption was measured at 530 and 657 nm with a microplate reader (Infinite 200 PRO, TECAN, Männedorf, Switzerland) to calculate anthocyanin contents with the formula *A*_530_ − 0.25 × *A*_657_ (Mancinelli, 1990). Shikimate level was spectrometrically determined based on previous literatures (Shaner et al., 2005; Sharkhuu et al., 2014). The aerial parts of two to three plants grown on the agarose plates under condition as indicated in Figure 8, B were pooled in a tube with 100 µL of 10 mM ammonium phosphate (pH 4.4). The samples were then immediately frozen in liquid nitrogen and thawed at 60°C for 30 min. After adding 25 µL of 1.25 N HCl, the samples were further incubated at 60°C for 20 min to extract metabolites. Twenty-five microliters of the resulting supernatant was mixed with 100 µL of a solution containing 0.25% (w/v) periodic acid and 0.25% (w/v) *m*-periodate and incubated at room temperature for 90 min. After stopping the reaction by adding 100 µL of a termination solution containing 0.6 N sodium hydroxide and 0.22 M sodium sulfite, the absorption at 380 nm was immediately measured with the microplate reader. Shikimate concentration was determined by comparing with a dilution series of the shikimate standards.

### Chemical feeding experiments

Chemical feeding experiments were carried out as described previously (de Oliveira et al., 2019) with some modifications. Seedlings were grown on agar plates containing 0.5 MS with 1% sucrose, 0.8% agar, and 2.5 mM MES at pH 5.7 in the growth chamber where we grew other plants in soil. AAAs and/or shikimate were added to autoclaved media after cooling down to temperatures below 55°C. Plants were grown on the same plate side by side to minimize environmental effects, and multiple plates were prepared to obtain replications. Root lengths of 10-day-old seedlings were quantified by ImageJ.

### Generation of transgenic Arabidopsis with Golden Gate cloning

AthDHS1 Pro5U (promoter plus 5′-UTR), AthDHS1 CDS, AthDHS1 3′-UTR, and Hygromycin resistance gene sequences were modified to remove TypeIIS restriction enzyme sites and obtained as synthetic DNA (Twist Bioscience, San Francisco, CA, USA) with BsaI restriction sites and overhangs compatible with the MoClo modular cloning system. A modified transformation booster sequence (TBS-1; Meyer et al., 1988; Hily et al., 2009) was obtained as gene module from Wisconsin Crop Innovation Center (WCIC, ST1594-8; Supplemental Data Set S2). TBS insulator was used between Hygromycin resistance and AthDHS transcription units (TUs) to avoid interference of 35S promoter to the native DHS promoters (Supplemental Figure S19). For AthDHS3 Pro5U, AthDHS3 gDNA, and AthDHS3 3′-UTR, PCR fragments were amplified using Phusion High-fidelity DNA polymerase (Thermo Fisher Scientific) accordingly to manufacture instructions (Supplemental Data Set S2). Additional modular parts were obtained from MoClo tool Kit (Kit # 1000000044, Addgene; Weber et al., 2011) and MoClo Plant parts kit (Kit # 1000000044, Addgene; Engler et al., 2014). Prior to cloning, all modules and acceptor vectors (Supplemental Data Set S2) were isolated and concentration adjusted to 100 ng/µL. TUs were assembled in a single reaction into Level 1 acceptors. The reaction mixture for Level 1 assembly consisted of 100 ng of Level 1 acceptor vector, 100 ng of each Level 0 module or PCR fragment, 1× Cut Smart Buffer (New England Biolabs, Ipswich, USA), 1 mM ATP (Thermo Fisher Scientific), 20U BsaIHFv2 (New England Biolabs), 400 U T_4_ DNA ligase (New England Biolabs), and ultrapure H_2_O to 20 µL final volume. The reaction was subjected to 25 cycles of 37°C for 3 min and 16°C for 4 min followed by incubation at 50°C for 5 min and inactivation at 80°C for 5 min, followed by storage at 4°C until transformation into *E. coli* competent cells. Positive colonies were selected on LB agar plate supplemented with 50 mg/L carbenicillin (Carb) and surface coated with 100 µL X-GAL/IPTG 10 mM solution (Dot Scientific, Burton, MI, USA). White colonies were selected and used to isolate plasmids containing the correctly assembled TU confirmed by PCR and restriction digestion pattern.

The assembly of Level 2 binary vectors was performed according to the above procedure using BbsI-HF (New England Biolabs) instead of BsaI and 2,000 U of T_4_ DNA ligase (New England Biolabs). Selection was performed on LB agar plates supplied with 50 mg/L kanamycin. In order to produce an empty vector control, the TU at position 3, containing the AthDHS TUs, was exchanged by the dummy TU (pICH54033, Supplemental Figure S19 and Supplemental Data Set S2). Colonies transformed with undigested Level 2 acceptor vector were distinguished by their red color. White colonies were grown and isolated plasmids were confirmed to contain correctly assembled construct based on restriction enzyme digestion patterns.

One confirmed binary vector for each construct was submitted to whole plasmid sequencing and assembly at the Center for Computational and Integrative Biology (CCIB) DNA Core Facility at the Massachusetts General Hospital (MGH), Cambridge, MA, USA. After sequencing, plasmids were transformed into *Agrobacterium tumefaciens* GV3101 by electroporation. Colonies were confirmed by PCR and then stored until further use in Arabidopsis transformation. Transformation into 5–6-week-old Arabidopsis plants was carried out using a modified floral dip method (Martinez-Trujillo et al., 2004). The vector with the dummy TU was also transformed into each mutant as the negative controls for the complementation tests. Successfully transformed T_1_ seeds were selected based on the red fluorescence under the Zeiss AxioZoom microscopy in the Newcomb Imaging Center at the University of Wisconsin–Madison and used for further experiments.

### Construction of *DHS* cladogram tree

*DHS* orthologs were first identified by BlastP searches utilizing the amino acid sequence of AthDHS1 as query against Phytozome and SpinachBase databases (Goodstein et al., 2012; Xu et al., 2017). All of the obtained sequences were then used to construct a tree of *DHS* genes using MEGA 7 (Kumar et al., 2016) and are available as a FASTA file in Supplemental Data Set S3. The sequences were aligned by the MUSCLE algorithm and then constructed into the tree based on the maximum-likelihood method with 1,000 bootstrap replicates (Supplemental Data Set S4).

### Preparation of chemical compounds used in enzymatic assay

For arogenate production, prephenate was enzymatically converted into arogenate by Arabidopsis prephenate aminotransferase (AT2G22250) recombinant enzyme and purified by an anion-exchange chromatography, as previously described (Maeda et al., 2010; Schenck et al., 2015). For treatment of AthCM2 (AT5G10870) recombinant protein to chorismate, 20 mM chorismate was incubated at 37°C for 1 h with approximately 30 µg of active or boiled AthCM2 enzymes in 100 µL of 50 mM HEPES (pH 7.4) buffer. After termination of the reaction by boiling at 100°C and cooling down, the solutions were used for the inhibitory assay of AthDHS1. For chemical conversion of arogenate into prephenate, 10 µL of 15 mM arogenate solution was incubated with 10 µL of 5 N HCl at 37°C for 20 min. After neutralization by adding 10 µL of 5 N NaOH, the resulting solution was used as 5 mM solution of HCl-treated arogenate. Caffeoyl shikimate and *p*-coumaroyl shikimate were synthesized by Dr. Dharshana Padmakshan in the laboratory of Prof. John Ralph, Department of Biochemistry and the Wisconsin Energy Institute at University of Wisconsin–Madison. The other chemicals including PEP, E4P, shikimate, chorismate, and caffeate were purchased from Millipore-Sigma with catalog numbers P7127, E0377, S5375, C1761, and C0625, respectively.

## Accession numbers

Sequence data from this article can be found in the EMBL/GenBank data libraries under the following accession numbers: *AthDHS1* (AT4G39980), *AthDHS2* (AT4G33510), and *AthDHS3* (AT1G22410).

## Supplemental data

**Supplemental Figure S1.** A cladogram tree of plant *DHS* genes.

**Supplemental Figure S2.** Requirement of Mn^2+^ and DTT for different AthDHS isoforms.

**Supplemental Figure S3.** Elimination of impact of contaminated bacterial DHS enzymes on AthDHS activity.

**Supplemental Figure S4.** Michaelis–Menten plots of AthDHS1, AthDHS2, and AthDHS3.

**Supplemental Figure S5.** Enzymatic assay of AthDHS enzymes in the presence of all the individual amino acids.

**Supplemental Figure S6.** Confirmation of chorismate-dependent AthDHS inhibition and its attenuation by arogenate.

**Supplemental Figure S7.** Chorismate-mediated inhibition of AthDHS1 is offset specifically by arogenate, but not by prephenate, aspartate, or shikimate.

**Supplemental Figure S8.** The enrichment statistical significance of GO-terms and KEGG pathways in *AthDHS* coexpression networks.

**Supplemental Figure S9.** Comprehensive gene expression survey using public transcriptome data.

**Supplemental Figure S10.** Root length measurement of the *dhs* mutants under high AAA conditions.

**Supplemental Figure S11.** Complementation tests of *dhs1* and *dhs3* in the presence of high concentrations of Tyr and Trp, respectively.

**Supplemental Figure S12.** Col-0 and the *dhs* mutants grown with AAA and/or shikimate.

**Supplemental Figure S13.** A growth picture of 14-day-old Col-0 and the *dhs* mutants in the presence of extra Tyr.

**Supplemental Figure S14.** Complementation of *dhs2* rescued the phenotypes of the leaf development and the shikimate accumulation under high Tyr condition.

**Supplemental Figure S15.** Enzymatic assay of crude extracts from Col-0 and the *dhs* mutants in the presence of individual AAA.

**Supplemental Figure S16.** Phenotypes of Col-0 and *dhs1* grown with MeJA.

**Supplemental Figure S17.** Accumulation of anthocyanins before and 2 and 5 days after high light treatment.

**Supplemental Figure S18.** Anthocyanin accumulation of the *dhs* complementation lines before and 2 and 5 days after HL treatment.

**Supplemental Figure S19.** Schematic diagrams of *AthDHS* gene constructs for complementation tests.

**Supplemental Table S1.** A list of top 20 genes co-expressed with *AthDHS1*, *AthDHS2*, and *AthDHS3*.

**Supplemental Table S2.** Levels of amino acids and AAA-derived metabolites under standard growth condition (before HL treatment) and after 2-day HL treatment in Col-0 and the *dhs* mutants.

**Supplemental Table S3.** Primer list used in this study.

**Supplemental Table S4.** Summary of peak information obtained by LC–MS and LC–MS/MS analyses.

**Supplemental Data Set S1.** Lists of gene information in the GO-terms and KEGG pathways enrichment analysis.

**Supplemental Data Set S2.** Plasmids, PCR fragments, and constructs used for GoldenGate cloning.

**Supplemental Data Set S3.** Sequences of plant *DHS* genes used for phylogenetic analysis.

**Supplemental Data Set S4.** A tree file of plant *DHS* genes.

## Supplementary Material

koaa042_Supplementary_Data

## Notes

R.Y. and H.A.M. designed the research. R.Y. performed most of the experiments. M.V.V.d.O. generated the transgenic plants. B.K. conducted the expression database search. R.Y. and H.A.M. analyzed the data, and R.Y., M.V.V.d.O., B.K., and H.A.M. wrote the manuscript.

The author responsible for distribution of materials integral to the findings presented in this article in accordance with the policy described in the Instructions for Authors (https://academic.oup.com/plcell/pages/General-Instructions) is: Hiroshi A. Maeda (maeda2@wisc.edu).
