# Supplementary material for: The entry reaction of the plant shikimate pathway is subjected to highly complex metabolite-mediated regulation
Source: Plant Cell. 2021 Jan 7;33(3):671–96. doi: 10.1093/plcell/koaa042 (PMC8136874; doi:10.1093/plcell/koaa042)
Supplement: koaa042_Supplementary_Data [file koaa042_supplementary_data.zip › tpc.00840.2020-s01.pdf]

THE PLANT CELL

Author Revision Checklist **Corresponding Author** Hiroshi Maeda **Ms #** TPC2020-RA-00840DR1

The following items are required prior to final acceptance. Assessment and acceptance of your submission may be delayed if these elements are not clearly presented or are found to be out of compliance with journal standards. Please complete this form and submit it along with your revised manuscript as supplemental material.

MATERIALS DISTRIBUTION

All manuscripts must include the following statement as an unnumbered footnote: "The author(s) responsible for distribution of materials integral to the findings presented in this article in accordance with the policy described in the Instructions for Authors (www.plantcell.org) is (are): John D. Author (author@college.edu)."

☑ Materials distribution statement is included in the manuscript.

DATA AVAILABILITY

Accession numbers must be provided for all genes reported and major genes discussed. All large-scale data (e.g., genome sequences, annotations, genetic maps, transcript profiles, other sequencing data, proteomic data sets, metabolic profiles) that are integral to the manuscript must be submitted to a permanent public repository with open access prior to submission, and must be made publically available immediately upon publication. Accession codes, unique identifiers, or web links for publicly available data sets must be provided in an Accession Numbers section at the end of the Methods.

☐ No data with mandated deposition ☐ All relevant accession numbers are provided

IMAGE INTEGRITY

Authors must ensure that all panels are accurate, all labels are correct, and no inadvertent duplications or errors occurred during preparation. Unprocessed source data must be provided upon request.

☑ Confirm that all data conform to image integrity guidelines listed in the Instructions for Authors.

NOMENCLATURE

All nomenclature used must conform to accepted community standards for the species studied. Arabidopsis nomenclature rules should not be used for certain other species (including maize and rice) and vice versa. Please see the Instructions for Authors for more details and links.

☑ All nomenclature used in the manuscript follows community guidelines for the relevant species.

All gene and protein symbols used must have priority in the literature. New gene names and symbols must be approved by the editors and should be compliant with the naming conventions of the relevant research community. New Arabidopsis gene names should be registered with TAIR.

☑ The manuscript is introducing one or more new gene or protein names, and an explanation of these new names and rationale for introducing them is provided in the manuscript cover letter.

METHODS REPORTING

☑ Plant growth conditions (soil, amendments, media, cell culture, etc.) are adequately described, including details of light quality - spectrum or bulb type – as well as intensity.

☑ PCR primers (RT-qPCR, cloning, etc.): all PCR primers are provided in the Methods or a supplemental table.

☑ Cloning information: cloning and DNA constructs are fully described (use a supplemental table if necessary).

☑ Antibodies: the source is provided for all commercial antibodies, including catalog/lot #, where applicable, or a complete description of non-commercial antibodies in the Methods (or as Supplemental Methods if appropriate).

REPLICATION AND STATISTICAL ANALYSES

Statistical analyses, the nature of replicates, and error bars must be adequately described in the Methods and figure legends. Confirm that the following items are present in the legends of all figures and tables that used statistical methods (or in the Methods section if appropriate):

n/a | confirmed

☐ | ☐ A complete description of how samples were harvested or collected, the precise nature of replicates, and the sample size (n) for each experimental group/condition. *Note that "biological replicate" alone is insufficient; this must be precisely defined for your experiments.*

☐ | ☐ A description of statistical test(s) used, whether they are one-sided or two-sided, rationale and assumptions, normalizations, and corrections (such as adjustment for multiple comparisons).

☐ | ☐ Test values indicating whether an effect is present. *Provide confidence intervals or give results of significance tests (e.g. p values).*

☐ | ☐ Clearly defined error bars in all relevant figure captions.

☐ | ☐ ANOVA and/or T-test results provided in supplemental tables (i.e. showing variables, parameters, degrees of freedom, and test statistics).

QUANTITATVE PCR

Methods used for qPCR must be described in full, including information on enzymes, kits, machines, normalization and data analysis, and genes/primers used (primers may be listed in a supplemental table). Details must be provided on statistical/analytical methods used to measure gene expression (RT-qPCR) and to determine differences in gene expression. Authors are encouraged to consult Remans et al. (2014). Reliable Gene Expression Analysis by Reverse Transcription-Quantitative PCR: Reporting and Minimizing the Uncertainty in Data Accuracy Plant Cell **26:** 3829-3837 and to follow MIQE guidelines. Note that use of the term "semi-quantitative" PCR is not permitted; assays must be shown to be sufficiently quantitative to support a conclusion of changes in levels.

n/a | confirmed

☐ | ☐ Complete methods and all primers for qPCR, RT-qPCR have been provided.

☐ | ☐ Differential gene expression was assessed using accepted statistical tests, which are fully described.

MACROMOLECULAR STRUCTURES

All relevant structural data must be submitted to an appropriate database (e.g. wwPDB, EMDB) prior to submission and must be made publicly available immediately upon publication.

The manuscript includes structural data (e.g. x-ray, NMR, EM).

☑ No | ☐ Yes:

☐ For all macromolecular structures studied, a validation report from wwPDB is provided.

☐ For any electron microscopy results, density maps and coordinate data have been deposited in EMDB.

SOFTWARE/CUSTOM SCRIPTS

Studies using customized software or custom scripts must describe how readers can access the code, including any access restrictions.

The manuscript includes custom codes or scripts.

☑ No | ☐ Yes:

☐ All custom codes, scripts, and pipelines are uploaded and available on a public repository such as GitHub and the direct link is listed within the manuscript. Downloadable materials include a ReadMe file and sample data file.

DATA PRESENTATION

Data distribution/bar charts and line charts. Authors are encouraged to present data in a format that shows data distribution (dot-plots, box-and-whisker plots, or violin plots), with all relevant elements defined (e.g. center line, median; box limits, upper and lower quartiles; whiskers, 1.5x interquartile range; points, outliers). If bar graphs are used, the corresponding dot plots should be overlaid. Individual data points should be shown in all cases where the number of data points <6.

☑ Confirm that in all cases where the number of data points is <6, individual data points are shown.

Supplemental Data. Data and methods that are integral to the main conclusions of the article must be presented in the main manuscript. Supplemental figures and tables must be prepared to the same standards of quality and visual clarity as regular manuscript figures and tables, with all data and elements of the figures clearly defined and fully explained.

☑ Confirm that each Supplemental Figure provides direct support for a figure in the main manuscript, and includes a supporting statement in the Supplemental Figure legend, e.g. ("Supports Figure 1").

Figures, Tables, and Supplemental Data Presentation

If your manuscript is accepted, figures, tables, and supplemental data will be required to conform to the guidelines below, which will be checked during a final scientific editing step. Careful preparation of your figures following these guidelines prior to resubmission will facilitate editor and reviewer assessment of your revised manuscript. Therefore, you may wish to make these changes prior to resubmission.

-Tables in the main manuscript or supplemental materials should be no more than 2 pages in length; any table longer than 2 pages should be provided in Excel format, labeled as a "Supplemental Data Set."

-Tables in the main manuscript must be included in the manuscript file (Word document) after the Methods section, using the "insert table" feature in Word (do not insert images into the manuscript file).

-Figures should be prepared as professionally as possible and should have a consistent appearance (i.e. as if all figures were made by the same person).

- All text and elements of each figure should be clearly visible at printed size.
- Color schemes should be used consistently across all figures, whenever possible (i.e. use the same colors for the same genotype/treatment across multiple figures); avoid complicated patterns and hatching if possible. Use a color palette that is "color blind safe" if possible, e.g. http://www.somersault1824.com/tips-for-designing-scientific-figures-for-color-blind-readers/
- Make consistent use of the same, sans-serif font (e.g. Arial) in all figures.
- Use the same (or similar) size font for panel letters (A, B, C, etc.) in all figures.
- Avoid the use of Excel default settings for creating graphs, i.e. for colors, patterns, bar-width, axis settings, etc.
- Bars in bar graphs should be the same width (or close) for multiple bar graphs in different panels of a single figure (*this means spaces between bars may be different, but bars should be the same width*).
- Use proper symbols for +/− (instead of a hyphen - for minus).
- Numerical values on all graph axes should have a consistent number of significant digits (e.g. 5.0, 10.0, 15.0 **or** 5, 10, 15 etc.); however, the origin should always be "0", never "0.0".
- Make sure axis values are legible and do not crowd the values along the axes.
- Images made from screen shots should be adjusted to high resolution where necessary, and illegible or overlapping text or other elements omitted or re-drawn using a suitable graphics program to be legible at printed size.
- Figure legends must be clear and adequately describe the data shown. All elements of figures must be defined accurately, e.g. the axes, abbreviations, symbols, how values were obtained, and error bars. Replicates must be defined precisely: the term "biological replicate" should be defined explicitly in the context of each experiment in the legend (or Methods).

- The journal is implementing a pilot project of identifying the author(s) responsible for creating each figure, table, or data set, to give proper attribution to authors for their individual contributions to a manuscript. If you would like to participate, please either include the information in the author contributions section, or add a statement to each legend, such as “J.D., A.S., and B.W. contributed the data and created the figures for panels A, B, and C, respectively”, or “J.D., A.S., and B.W. contributed the data for panels A, B, and C, respectively, and J.D. created the figure.”

- Multiple supplemental figures and tables should be combined and submitted as a single PDF file named “Supplemental Data”, no larger than 50 MB. Files to be merged into the single supplemental data file include supplemental figures, followed by supplemental tables. Supplemental tables in this file are restricted to 1-2 pages in length.

-Any Supplemental Table longer than 2 pages should be labeled as a “Supplemental Data Set” and provided in Excel format.

-Arial font (or similar sans serif font, e.g. Helvetica) should be used on all Supplemental Figures, Supplemental Tables, and Supplemental Data Sets and their corresponding legends. The only exception is for nucleotide sequences (primers, etc.) where a mono-spaced font such as Courier should be used.

-Supplemental Data Sets should include complete legends (on a separate sheet if necessary).

-Numerical values in data sets should be adjusted to a suitable number of significant figures (decimal places); do not simply use default Excel settings.

-Make use of freeze panes and shading or color coding to help the reader navigate large files. If convenient, multiple Supplemental Data Sets may be submitted on separate sheets in a single Excel file.

-File size: each supplemental file ideally should be less than 10 MB and is limited to 50 MB maximum size. *If you have a large number of supplemental files or files that exceed 50 MB, you may be requested to upload supplemental data to datadryad.org.*

Resources

https://betterfigures.org/2015/06/23/picking-a-colour-scale-for-scientific-graphics/

Ten simple rules for better figures (2014) by Rougier et al. in *PLOS Computational Biology*

FUNDING INFORMATION

Funding information provided during the submission process online should match what is in the manuscript file. *Please check this when you resubmit.*

SPECIFIC METHODS MODULES

Certain methods require completion of a specific module in addition to this form. Please complete the section below and the relevant module(s) (found on pages 7-10) for specific methods/data types listed.

n/a | confirmed

☐ | ☐ For newly generated genetic material (e.g. mutant, transgenic lines), confirm completion of genetic materials module on page 7.

☑ | ☐ For protein-protein or protein-nucleic acid interactions, confirm completion of the protein interactions module on page 8.

☑ | ☐ For protein localization studies, confirm completion of the protein localization module on page 9.

☐ | ☐ For phylogenetic analysis, confirm completion of the phylogenetics module on page 10.

THE PLANT CELL

MANUSCRIPT FORMAT

Title and abstract. The title and abstract are critical for engaging readers and enticing them to read the paper. The title should be concise, use active voice, spell out genes names (or otherwise define), and avoid acronyms. The species or organism under study should be in the title or the abstract (i.e. brief description of experimental system(s) used). See: http://genestogenomes.org/how-to-write-titles-that-tempt/. The abstract should be no more than ~200 words. Include only major results and implications and put general background and methods in the main text.

One-sentence summary. Include a one-sentence summary of the work on the title page that provides a non-technical summary of the broad significance of the research findings in plain language (for a general audience) that does not merely repeat what is already in the abstract. The use of complex scientific terms and acronyms should be avoided.

References: Upon final submission, in-text citations should be formatted using an author-date format; ***not numbered***, with references in alphabetical order in the Reference list. Any author-date style is acceptable. References cited only in the Supplemental Data file should be listed at the end of this file and not in the Reference list in the main text

Species names: Organisms should be referred to by their common name at first use (if a standard common name applies), with the Latin name in parentheses. Subsequent references can be either Latin or common names but should be consistent throughout the manuscript.

Title page: Please follow the format shown on the next page. Use single spacing of abstract and other information if necessary to fit title, author info, and abstract on a single page as shown (the abstract may run on to the second page if necessary).

SAMPLE TITLE PAGE

RESEARCH ARTICLE [or e.g. LARGE-SCALE BIOLOGY ARTICLE, BREAKTHROUGH REPORT]

A Dedicated Type II NADPH Dehydrogenase Performs the Penultimate Step in the Biosynthesis of Vitamin K1 in Synechocystis and Arabidopsis

Abdelhak Fatihi[a], Scott Latimer[a], Stefan Schmollinger[b], Anna Block[a], Patrick H. Dussault[c], Wim F.J. Vermaas[d], Sabeeha S. Merchant[e], Gilles J. Basset[a,f]

[a] Department of Agronomy and Horticulture, and Center for Plant Science Innovation, University of Nebraska, Lincoln, Nebraska 68588.

[b] Department of Chemistry and Biochemistry, University of California, Los Angeles, California 90095.

[c] Department of Chemistry, University of Nebraska, Lincoln, Nebraska 68588.

[d] School of Life Sciences and Center for the Study of Early Events in Photosynthesis, Arizona State University, Tempe, Arizona 85287

[e] Department of Chemistry and Biochemistry, University of California, Los Angeles, California 90095Institute for Genomics and Proteomics, University of California, Los Angeles, California 90095

[f]Corresponding Author**:** gbasset2@unl.edu

Short title: Transmethylation in Vitamin K Biosynthesis

One-sentence summary: The biosynthesis of vitamin K1 requires an additional bona fide step that consists of the prerequisite reduction of the demethylnaphthoquinone ring prior to its transmethylation.

The author responsible for distribution of materials integral to the findings presented in this article in accordance with the policy described in the Instructions for Authors (www.plantcell.org) is: Giles Bassett (gbasset2@unl.edu).

ABSTRACT

Mutation of *Arabidopsis thaliana NAD(P)H DEHYDROGENASE C1* (*NDC1*; *At5g08740*) results in the accumulation of demethylphylloquinone, a late biosynthetic intermediate of vitamin K1. Gene coexpression and phylogenomics analyses showed that conserved functional associations occur between vitamin K biosynthesis and NDC1 homologs throughout the prokaryotic and eukaryotic lineages. Deletion of *Synechocystis ndbB*, which encodes for one such homolog, resulted in the same defects as those observed in the cyanobacterial demethylnaphthoquinone methyltransferase knockout. Chemical modeling and assay of purified demethylnaphthoquinone methyltransferase demonstrated that, by virtue of the strong electrophilic nature of S-adenosyl-Lmethionine, the transmethylation of the demethylated precursor of vitamin K is strictly dependent on the reduced form of its naphthoquinone ring. NDC1 was shown to catalyze such a prerequisite reduction by using NADPH and demethylphylloquinone as substrates and flavine adenine dinucleotide as a cofactor. NDC1 displayed Michaelis-Menten kinetics and was markedly inhibited by dicumarol, a competitive inhibitor of naphthoquinone oxidoreductases. These data demonstrate that the reduction of the demethylnaphthoquinone ring represents an authentic step in the biosynthetic pathway of vitamin K, that this reaction is enzymatically driven, and that a selection pressure is operating to retain type II NAD(P)H dehydrogenases in this process.

THE PLANT CELL

GENETIC MATERIALS MODULE

Authors must provide details related to the generation and characterization of new genetic material (e.g., mutant or transgenic lines). For transgenes and mutant lines, the manuscript must include information on the number of independent transformation events, how many lines were isolated and characterized, and in which generation (T1, T2). Evidence that the lines have single or multiple insertion sites, or single or multiple copies of the transgenes, should be provided if available.

If the work is attempting to link a phenotype to a specific gene, evidence of a) multiple alleles/transgenes and/or b) multiple approaches each with single events (such as EMS alleles, transposon insertions, or Cas9-mediated lesions), and validation with sequencing may be important to insure that linked variation and off-target or position effects are not producing non-representative consequences. The use of RNAi, for example, is known to be associated with a high incidence of off-target effects (downregulation of unintended targets), underscoring the need for multiple lines of evidence.

CRISPR/Cas9-mediated mutations: Although it may not be feasible to use two different guide RNA transgenes, different mutations derived from the same guide RNA are not truly independent, as they may be linked to the same off-target effects. At a minimum, the guide RNA transgene should be segregated away from the mutation of interest and/or multiple independent transformation events used.

Gene names: Gene names should conform to species-specific nomenclature guidelines. Any previously published gene names have priority and should be used rather than re-naming genes. Please ensure that you have completed the nomenclature section on the author revisions checklist and provided an explanation and rationale for any new names in your cover letter when resubmitting your manuscript.

Please enter a description of each mutant or transgenic line below; provide a separate sheet if necessary.

Mutant or transgenic line	Description
dhs1 (SALK_055360)	Arabidopsis T-DNA insertional mutant of AthDHS1
dhs2 (SALK_033389)	Arabidopsis T-DNA insertional mutant of AthDHS2
dhs3 (SK2559S)	Arabidopsis T-DNA insertional mutant of AthDHS3
dhs1 Empty	transgenic line having the empty vector in the dhs1 mutant background
dhs1 AthDHS1	transgenic line expressing AthDHS1 with its own promoter in the dhs1 mutant background
dhs2 Empty	transgenic line having the empty vector in the dhs2 mutant background
dhs2 AthDHS2	transgenic line expressing AthDHS2 with its own promoter in the dhs2 mutant background
dhs3 Empty	transgenic line having the empty vector in the dhs3 mutant background
dhs3 AthDHS3	transgenic line expressing AthDHS3 with its own promoter in the dhs3 mutant background

THE PLANT CELL

PROTEIN INTERACTIONS MODULE

Please complete all numbered sections 1-5 below. Of particular importance for all assays is the choice and description of controls and quantitative information on the number of observations made.

1. ☑ Confirm that quantitative information is provided on the number of cells observed or observations made for all assays reported.

   It is desirable to show quantitative data (for example, from 10 randomly chosen regions of interest of infiltrated leaves) and not solely one or two "representative images." The minimum information that should be provided along with representative images is the number of cells/protoplasts or observations that showed the same localization pattern and the total number of cells observed or observations made. Where feasible, any representative microscopy image should show a complete cell, with the nucleus and the center of the cell in the focal plane.

PROTEIN-PROTEIN INTERACTIONS REPORTING

2. The manuscript includes BiFC analysis.

☑ No | ☐ Yes:

☐ Confirm that appropriate negative controls are included as described below.

Expressing unfused YFP fragments alone is not a sufficient control for BiFC experiments (see the Commentary from Bock and Kudla https://doi.org/10.1105/tpc.16.00043). Ideally, negative controls should include a mutated version of one of the interacting proteins carrying a defect in the interaction domain or a related non-interacting protein from the same protein family. If neither a mutated protein version nor a suitable closely related protein are available as negative controls, an unrelated protein (but, ideally, structurally similar and expressed in the same subcellular compartment) can be used.

☐ Confirm that the methods provides orientations of all constructs used.

It is essential that exactly the same orientations are used for negative controls as for the positive interaction.

3. The manuscript includes another type of protein-protein interaction assay (e.g. Y2H or variant, Co-IP, FRET).

☑ No | ☐ Yes. Attach another page if necessary.

Type of assay____

Description of controls:

PROTEIN-NUCLEIC ACID INTERACTIONS REPORTING

4. The manuscript includes ChIP-Seq experiments.

☑ No | ☐ Yes

☐ Confirm that both raw and final processed data have been deposited in a public database such as GEO.

☐ Confirm that access is provided to graph files (e.g. BED files) for called peaks.

☐ Confirm that the Methods section includes details of i) experimental replicates, ii) sequencing depth, iii) a description of all antibodies used, iv) peak calling parameters, and v) software used to collect and analyze ChIP-Seq data.

5. The manuscript includes EMSA experiments.

☑ No | ☐ Yes

☐ Confirm that any competition assay for binding specificity includes one or more controls using mutated or scrambled binding sites (showing no loss of binding); it is not meaningful to show competition with unlabeled wild-type DNA unless the competition with a mutated or scrambled site is included.

THE PLANT CELL

PROTEIN LOCALIZATION MODULE

Please note the following:

- For any immunological work, the source and specific characteristics of the antibody must be provided.
- When using a fluorescent marker (e.g. GFP) fused to a protein of interest, it may be important to show that the fusion protein a) is intact (e.g. via immunoblotting), and b) is co-expressed in the same location as a bona fide marker protein (e.g. for nucleus, vacuole, etc.) fused to a different spectral variant fluorescent marker. Where relevant, it may also be important to show that the fusion protein is functional. Authors should consider if any of this information is relevant and obtainable for their particular protein(s) and system under investigation.
- Information provided here must also be presented in the manuscript.

Describe the nature of protein localization experiments, controls used, and assessment.

Describe the source and specific characteristics of any antibodies used.

☐ Confirm that the manuscript provides quantitative information on the number of cells (protoplasts) observed or observations made.

It is desirable to show quantitative data (for example, from 10 randomly chosen regions of interest of infiltrated leaves) and not merely one or two "representative images", especially if only transient transformation experiments were conducted. The minimum information that should be provided along with representative images is the number of cells/protoplasts that showed the same localization and the total number of cells observed. Any representative image should display a complete cell, with the nucleus and the center of the cell in the focal plane.

THE PLANT CELL

PHYLOGENETICS MODULE

☑ Methods used for sequence analysis are reported in full with citations and software and parameter values (even if only default values were used) in a separate section of the Methods entitled "Phylogenetic Analysis". Please note that CLUSTAL does not produce an acceptable phylogeny; use a true phylogenetic analysis program (e.g., MEGA, RAxML, IQ-TREE, RevBayes, BEAST). Alignments used to produce phylogenies should be produced with an appropriate alignment program (e.g., MAFFT, T-Coffee).

☑ Confirm that for phylogenetic trees depicted or interpreted as rooted, the criterion used for rooting (e.g., midpoint, outgroup) is provided, and if outgroup rooting is used, the basis for the choice of outgroup is explained.

☑ Confirm that tree branch lengths (e.g. time, substitutions per site, coalescence units) are properly described in tree figure captions.

☑ Confirm that statistical support for nodes in any phylogenetic tree figures is reported (i.e., posterior probabilities or bootstrap values with MCMC search sample or replicate numbers reported in text).

☑ Sequence alignments (e.g. FASTA, PHYLIP, Nexus format) have been have been placed in a persistent database (e.g. Dryad, TreeBASE) or provided as Supplemental Files.

☑ Machine-readable tree files (e.g. Newick, Nexus, NeXML format) have been placed in a persistent database (e.g. Dryad, TreeBASE) or provided as Supplemental Data (preferably as a text [.tree] file labeled as a "Supplemental File").

☐ For instances where any of the above criteria are not relevant, please check this box and provide an explanation below:

Notes about terminology:

Similarity-based phenetic analyses – e.g. clustering of genotypes based on SNP data – should be distinguished from phylogenetic inference of gene or species relationships.

Authors are asked to avoid the use of terms such as “early diverging lineage”, “primitive”, “lower and higher plants”, and similar phrases, with reference to some non-flowering plants, e.g. *Physcomitrella*. This terminology is misleading as it serves to reinforce the idea that certain lineages are somehow primitive or ancestral to "higher" plants. The last common ancestor of *Arabidopsis* and *Physcomitrella*, for example, lived ca. 440 MYA and diverged into two lineages, one leading to vascular plants and one to mosses, both of which are alive today with a combination of ancestral and derived traits. It is nonsensical to state that one of these lineages is “earlier diverging” compared to the other. It is likewise misleading to call *Physcomitrella* “primitive”; it is only appropriate to refer to specific traits, such as gametophyte dominance, as being “ancestral”, rather than “primitive”. The term “basal angiosperm” is similarly misleading; more appropriate terminology for, e.g. *Amborella*, is “sister lineage to all other extant angiosperms”, or “ANA grade” (comprising Amborellales, Nymphaeales, and Austrobaileyales that are successively sister to all other extant angiosperms). Instead of the terms "higher" and "lower" plants, which are imprecise and misleading, substitute something more precise, e.g. "land plants,” "vascular plants," "angiosperms," "non-vascular plants," "bryophytes," etc.
