# Supplementary material for: The entry reaction of the plant shikimate pathway is subjected to highly complex metabolite-mediated regulation
Source: Plant Cell. 2021 Jan 7;33(3):671–96. doi: 10.1093/plcell/koaa042 (PMC8136874; doi:10.1093/plcell/koaa042)
Supplement: koaa042_Supplementary_Data [file koaa042_supplementary_data.zip › tpc.00840.2020-s02.pdf]

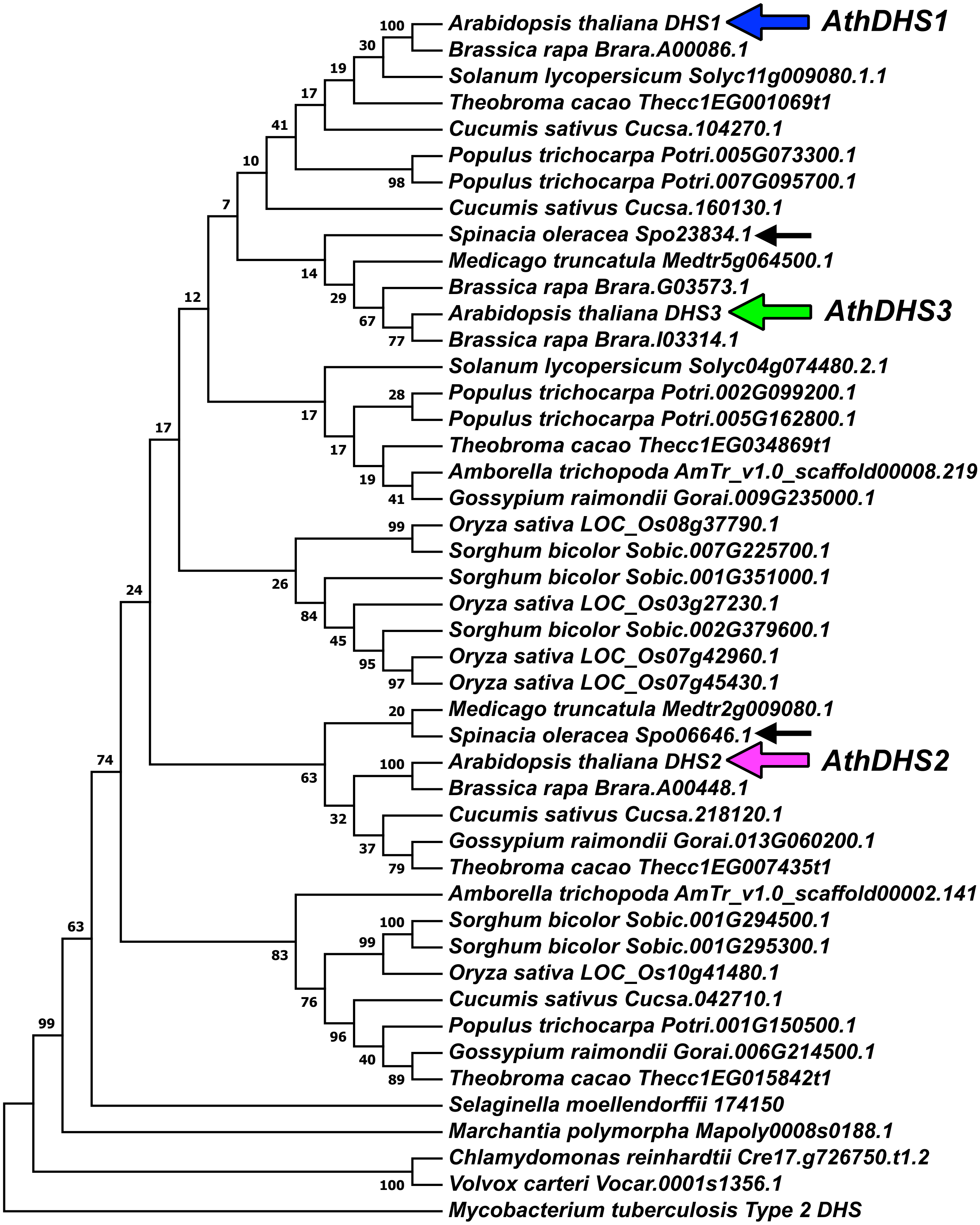

Supplemental Figure 1. (supports Figures 2 to 5) A cladogram tree of plant *DHS* genes. A phylogenetic tree of *DHS* orthologs from the the plant kingdom were constructed using the Maximum likelihood method with 1,000 bootstrap replicates. *Mycobacterium tuberculosis DHS* was used as the outgroup of Type II *DHS* genes. *Arabidopsis thaliana* and *Spinacia oleracea DHS* genes are indicated by colored and black arrows, respectively.

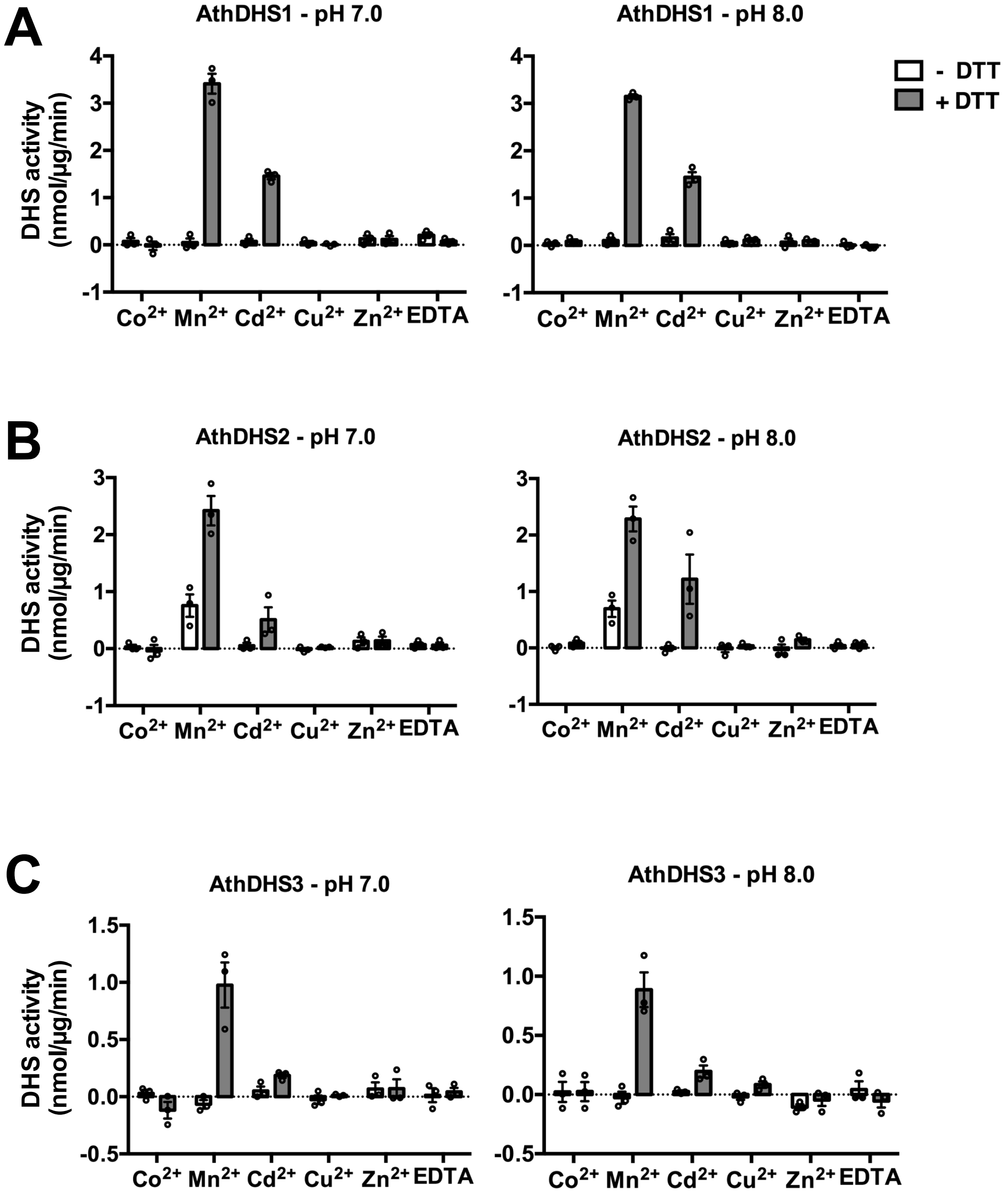

Supplemental Figure 2. (supports Figure 2) Requirement of Mn^{2+} and DTT for different AthDHS isoforms. Enzymatic assays of AthDHS1 (A), AthDHS2 (B) and AthDHS3 (C) using 2 mM Co^{2+}, Mn^{2+}, Cd^{2+}, Cu^{2+} or Zn^{2+}, or 1 mM EDTA in the absence or presence of 5 mM reducing agent DTT with pH 7.0 (left) and 8.0 (right). The results of AthDHS activity with Mn^{2+} at pH 7.0 were summarized in **Figure 2A**. Data are means ± SEM (n = 3 replicated reactions). All the individual data points are shown as dots.

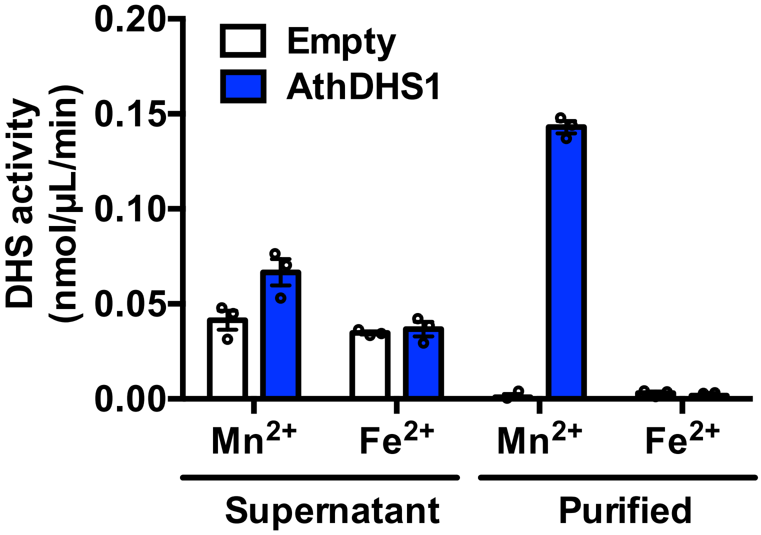

Supplemental Figure 3. (supports Figure 2) Elimination of impact of contaminated bacterial DHS enzymes on AthDHS activity. DHS enzymatic assays were carried out using protein samples isolated from bacteria carrying empty or *AthDHS1*-encoded vectors. Supernatant fractions that correspond to soluble proteins just before applying to affinity purification were used to conduct this assay in the presence of Mn^{2+} and Fe^{2+} at 2 mM. Y-axis represents DAHP production per minute and protein volume added into the reaction. Data are means ± SEM (n = 3 replicated reactions). All the individual data points are shown as dots.

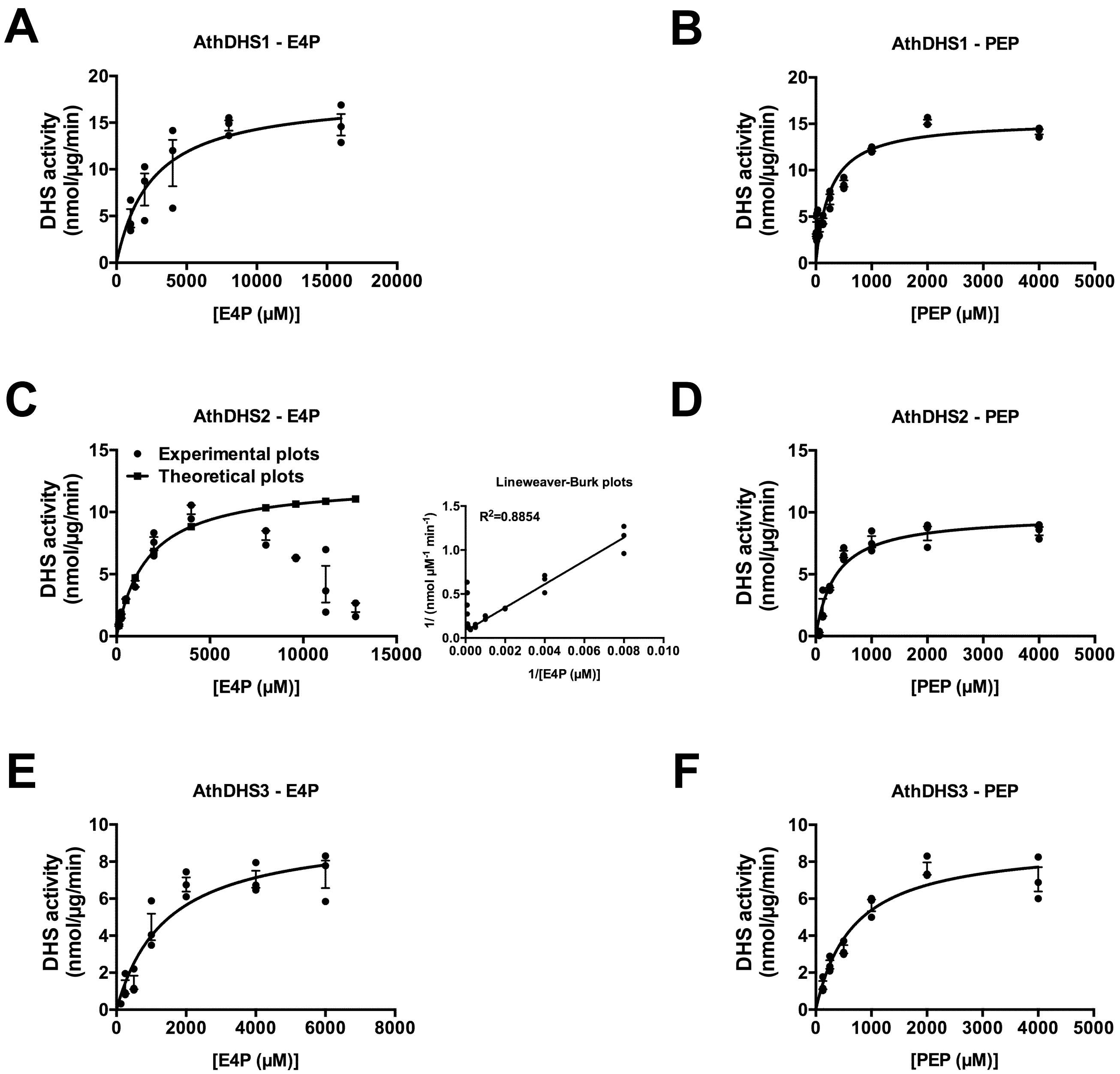

Supplemental Figure 4. (supports Table 1) Michaelis–Menten plots of AthDHS1, AthDHS2 and AthDHS3. AthDHS1, AthDHS2 and AthDHS3 activities with various concentrations of the substrates were measured to determine their kinetic parameters by fitting to the Michaelis-Menten equation. Since AthDHS2 showed inhibition by E4P at the high concentration, predicted curve were generated using the Michaelis-Menten kinetics after truncating the inhibited rates at high E4P concentration, assuming that those curves represent the activity that would be detected if no substrate inhibition occurred (Bernstein et al., 1978). For V_{max} and K_m determination, data points were chosen based on the best fits to the Lineweaver-Burk plots (R^2 = 0.8854). Calculated V_{max}, K_m, k_{cat} and k_{cat}/K_m are summarized in **Table 1**. Data are means ± SEM (n = 3 replicated reactions). All the individual data points are shown as dots.

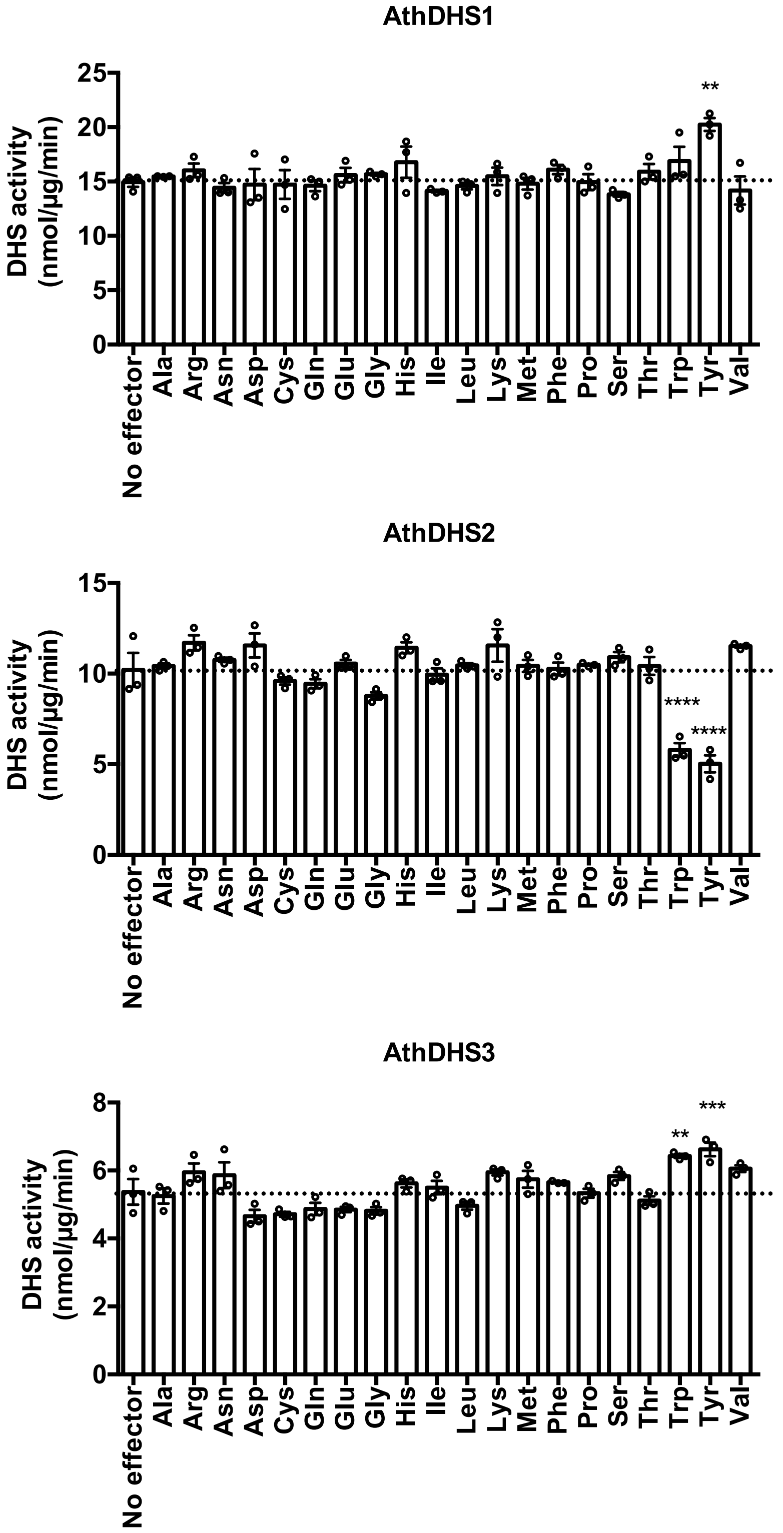

Supplemental Figure 5. (supports Figure 2) Enzymatic assay of AthDHS enzymes in the presence of all the individual amino acids. Activities of AthDHS1 **(A)**, AthDHS2 **(B)** and AthDHS3 **(C)** were measured using all the individual 20 amino acids at 1 mM concentration. **$P \leq 0.01$, ***$P \leq 0.001$ and ****$P \leq 0.0001$ denote significant differences by one-way ANOVA against corresponding “No effector” control samples. Data are means ± SEM (n = 3 replicated reactions). All the individual data points are shown as dots. The level of each “No effector” sample was indicated by a dotted horizontal line.

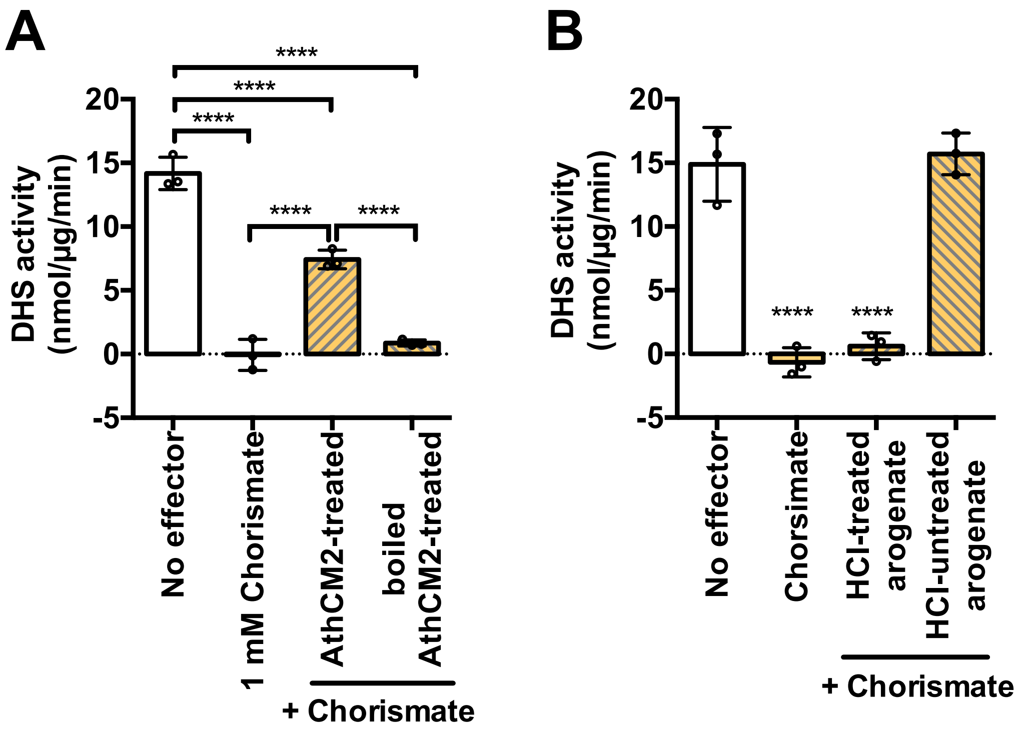

Supplemental Figure 6. (supports Figure 3) Confirmation of chorismate-dependent AthDHS inhibtion and its attenuation by arogenate. **(A)** AthDHS1 activity assay using 1 mM chorismate that was incubated with active or boiled AthCM2 enzymes for one hour. **(B)** AthDHS1 assays with 1 mM chorismate in the presence and absence of 1 mM arogenate that was treated or untreated with 1N HCl. ****$P \leq 0.0001$ denotes significant differences by one-way ANOVA against corresponding “No effector” or “AthCM2-treated 1 mM Chorismate” samples. Data are means ± SEM (n = 3 replicated reactions). All the individual data points are shown as dots.

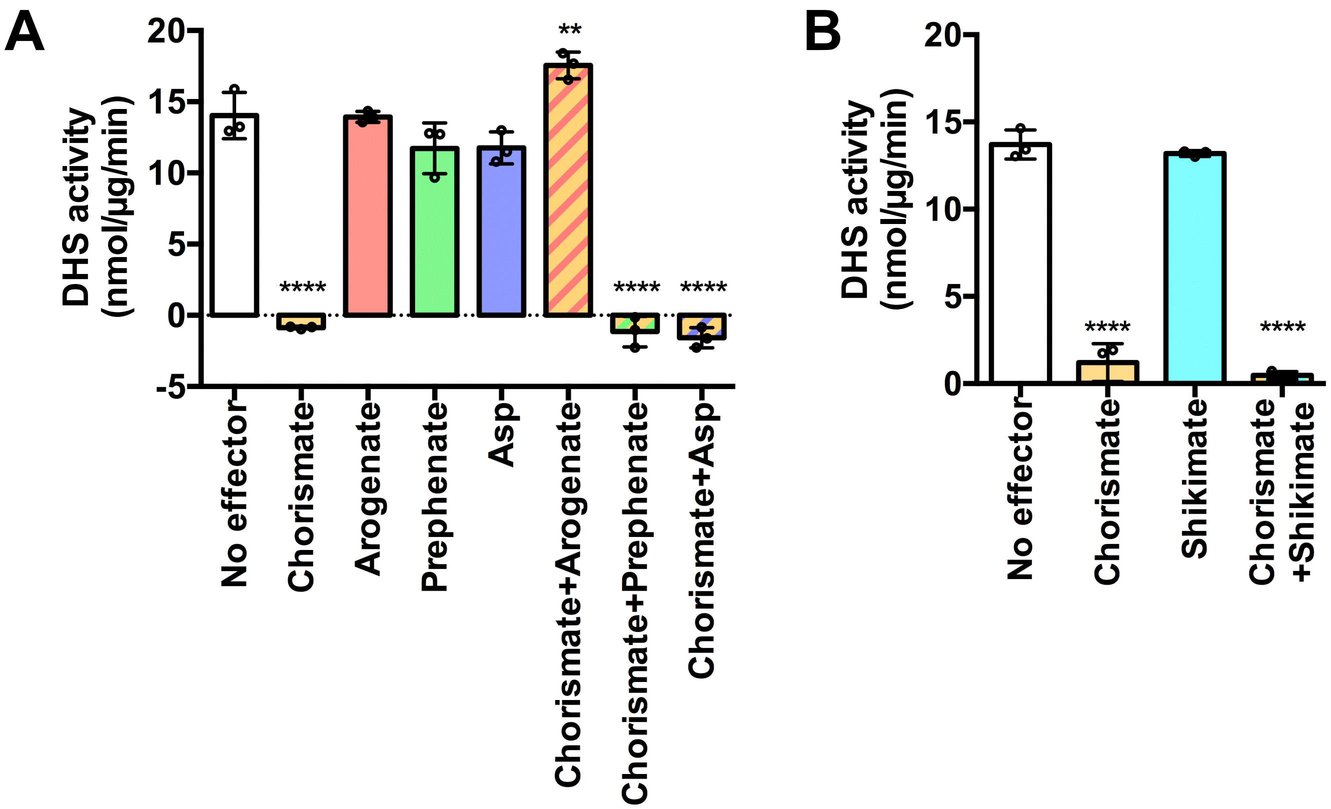

Supplemental Figure 7. (supports Figure 3) Chorismate-mediated inhibition of AthDHS1 is offset specifically by arogenate, but not by prephenate, aspartate, or shikimate. AthDHS1 activity was measured using chorismate, arogenate, prephenate or aspartate (Asp) alone, or their combination at 1 mM concentration (A) or using chorismate or shikimate alone, or their combination at 1 mM concentration (B). $^{**}P \leq 0.01$ and $^{****}P \leq 0.0001$ denote significant differences by one-way ANOVA against corresponding “No effector” control samples. Data are means ± SEM (n = 3 replicated reactions). All the individual data points are shown as dots.

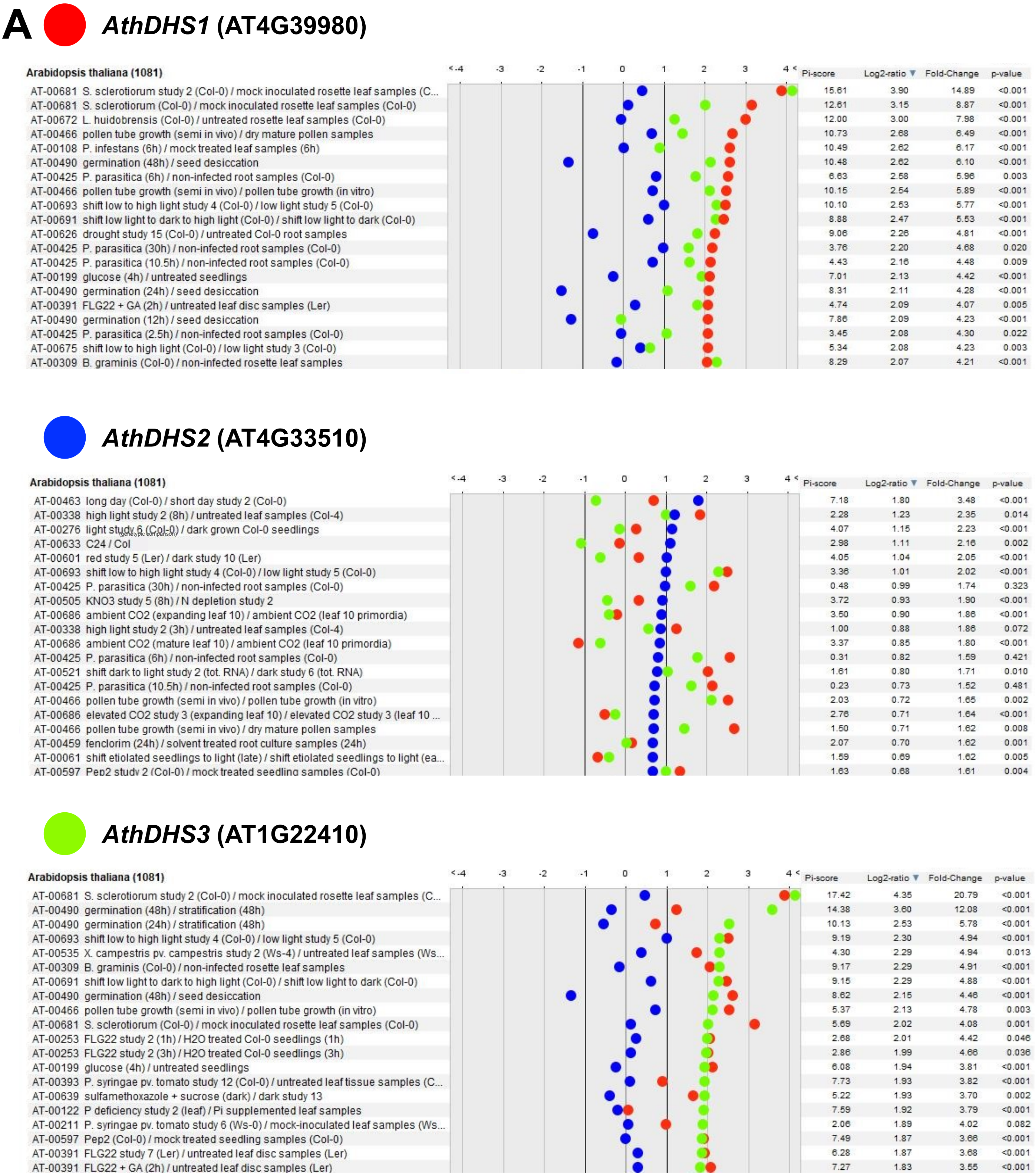

Supplemental Figure 9. (supports Figure 5) Comprehensive gene expression survey using public transcriptome data. **(A)** A list of the twenty most significant perturbations on the upregulation of *Arabidopsis thaliana DHS* genes. Scatterplots of the average of the logarithmic values were created using the Perturbations condition search tool in the Professional edition of Genevestigator (Hruz et al., 2008). Data selection from the Affymetrix Arabidopsis ATH1 Genome Array platform was filtered for only wild-type genetic background and refined by excluding all categories with less than 3 independent samples. All samples from the starting data selection of 5,253 samples were included in the perturbation analysis.

B

● ***AthDHS1* (AT4G39980)**

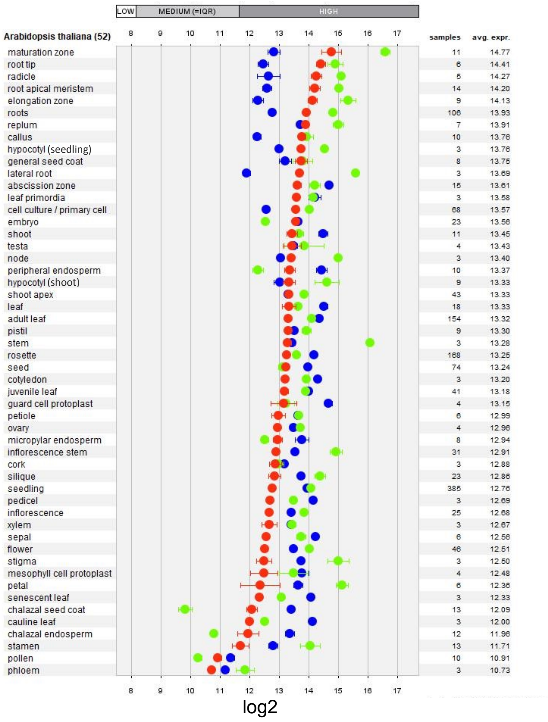

● ***AthDHS2* (AT4G33510)**

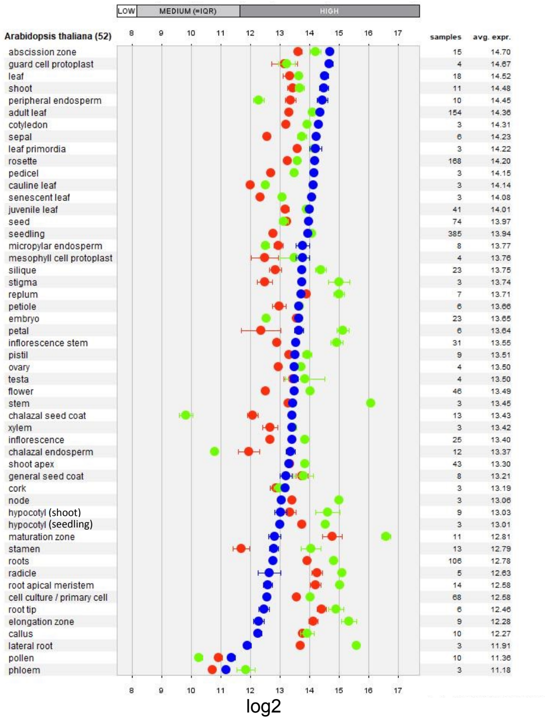

● ***AthDHS3* (AT1G22410)**

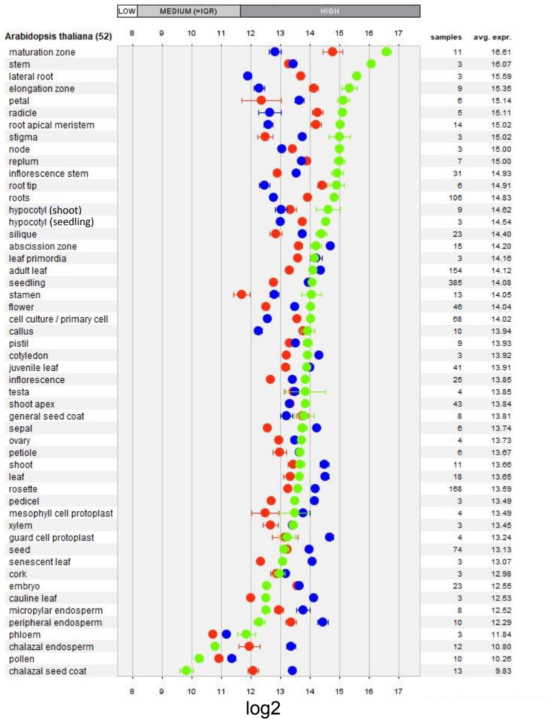

Supplemental Figure 9. (continued) (B) Tissue specific expression patterns of Arabidopsis thaliana *DHS* genes. Scatterplots of the average of the logarithmic values were created using the Anatomy condition search tool in the Professional edition of Genevestigator (Hruz et al., 2008). Data selection from the Affymetrix Arabidopsis ATH1 Genome Array platform was filtered for only wild-type genetic background samples without any stimulus experiments, and further refined by excluding ones with less than three independent samples. The starting data selection of 5,253 samples in 68 anatomical parts was refined to 1,458 samples in 52 anatomical parts.

C

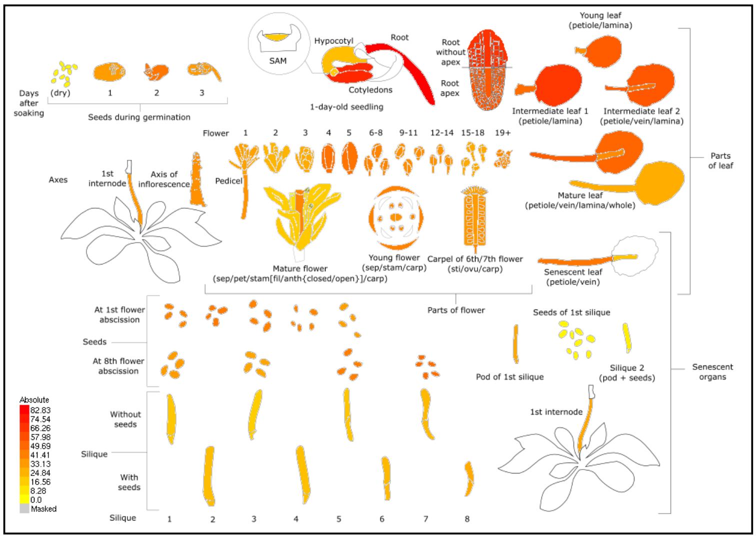

***AthDHS1* (AT4G39980)**

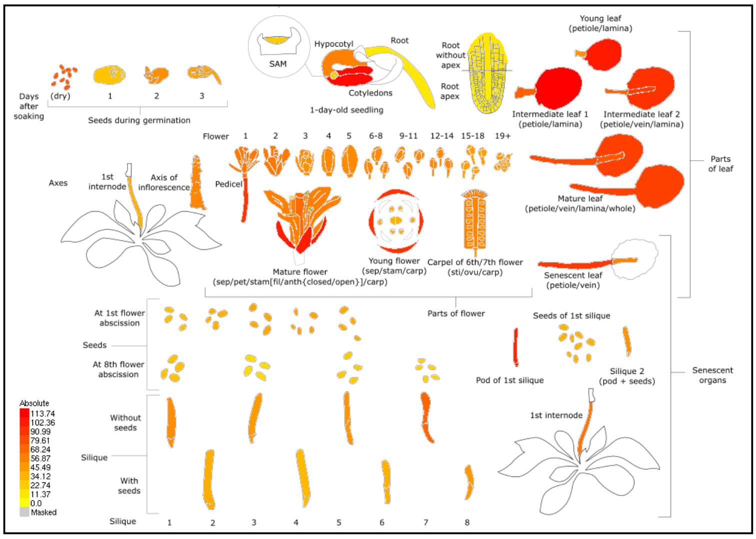

***AthDHS2* (AT4G33510)**

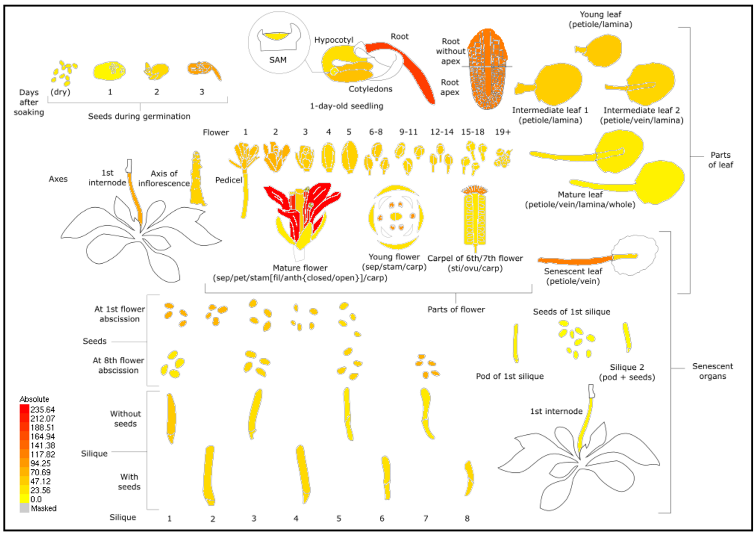

***AthDHS3* (AT1G22410)**

Supplemental Figure 9. (continued) (C) Absolute expression levels of *AthDHS1*, *AthDHS2* and *AthDHS3* genes in different tissues. Images were generated with the Plant eFP browser (Klepikova et al., 2016).

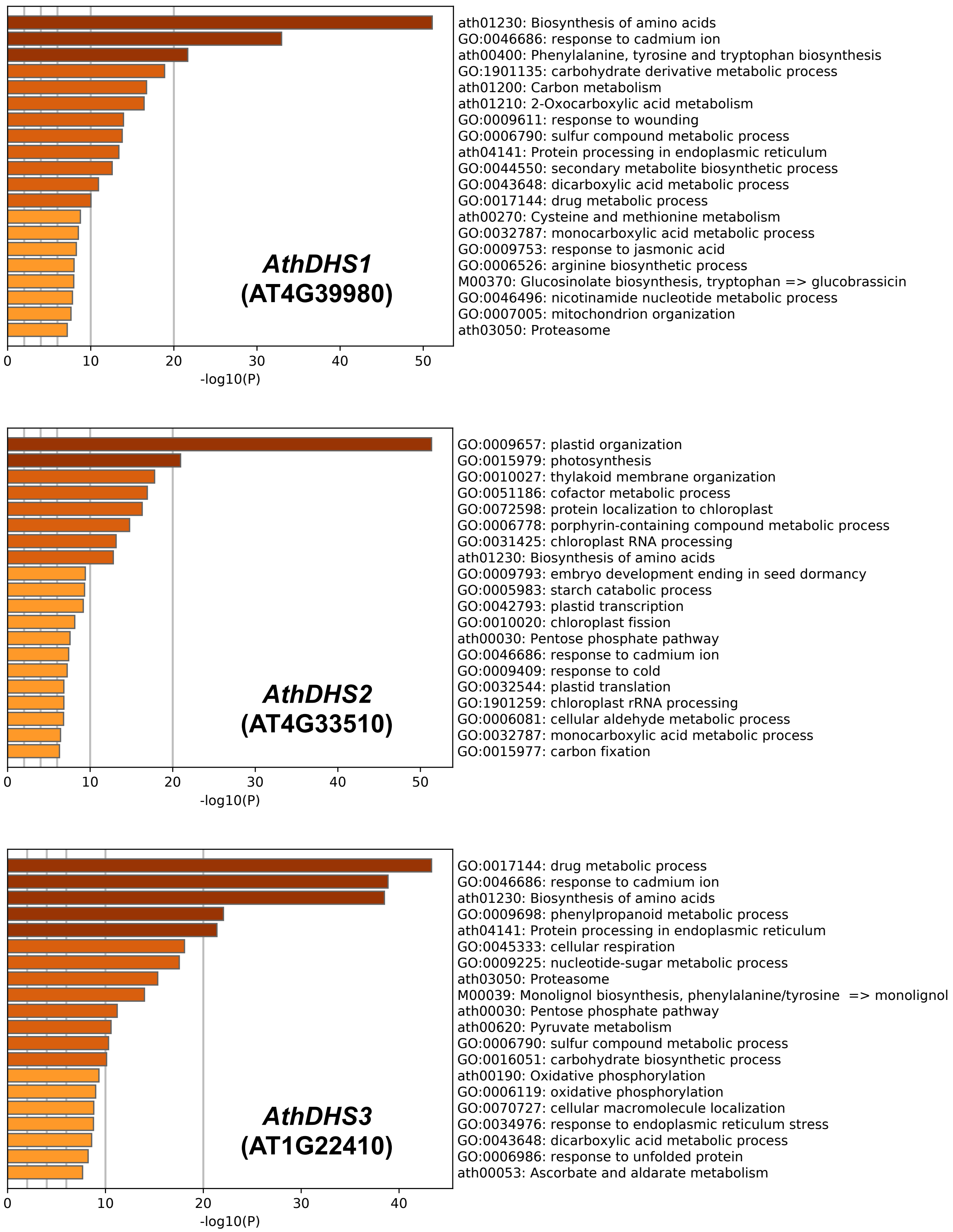

Supplemental Figure 8. (supports Figure 5) The enrichment statistical significance of GO-terms and KEGG pathways in *AthDHS* coexpression networks. GO and KEGG pathway enrichment analyses were performed in Metascape with the cutoff criteria *p* value < 0.01 (Zhou et al., 2019), using top 1000 genes co-expressed with each *AthDHS* genes, which were obtained from ATTED-II (Obayashi et al., 2018) and are available in **Supplemental Data Set 1**. Lists of the top 20 co-expression genes with *AthDHS* genes are also shown in **Supplemental Table 1**.

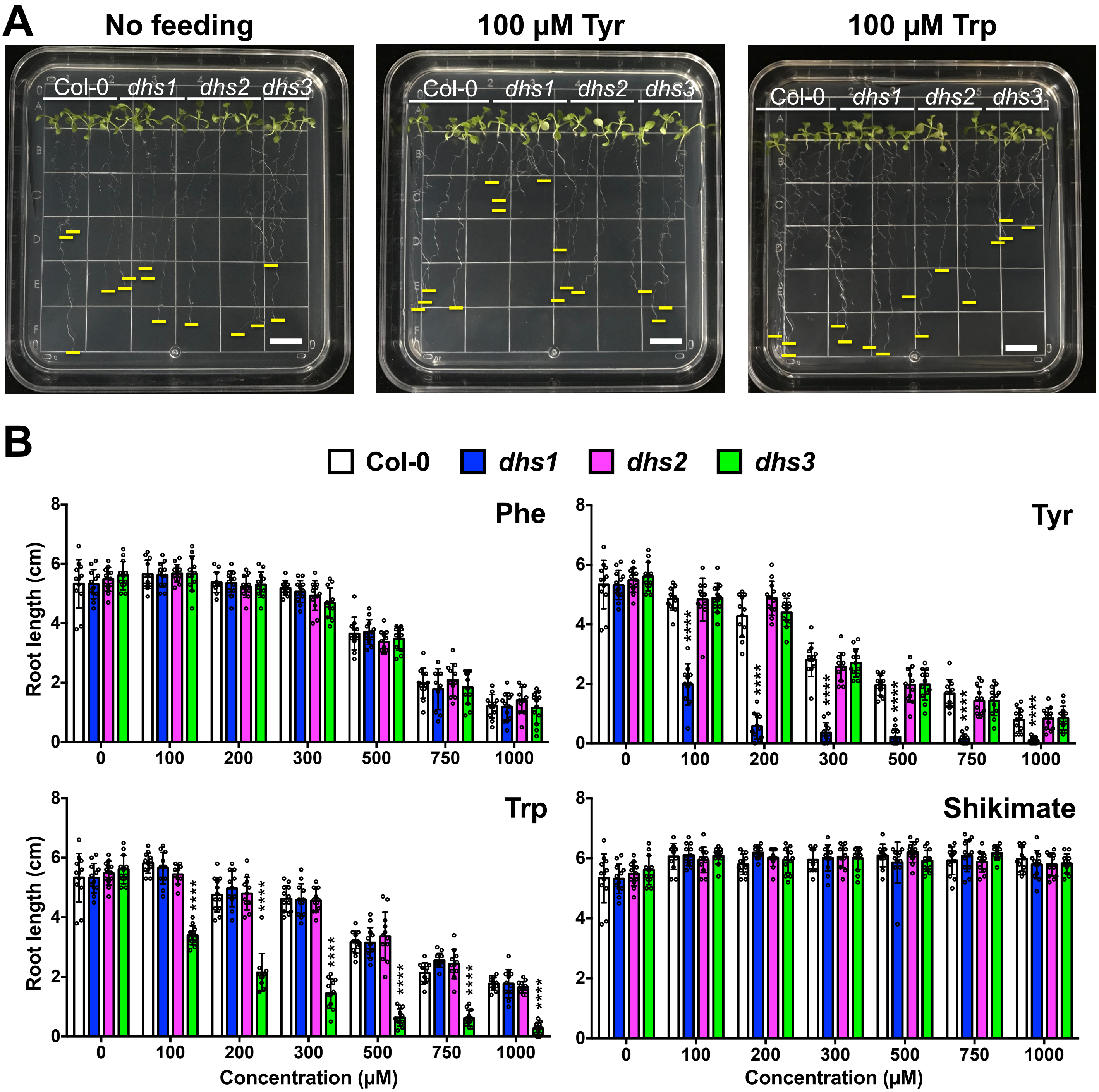

Supplemental Figure 10. (supports Figure 7) Root length measurement of the *dhs* mutants under high AAA conditions. **(A)** Root growth pictures of 10-day-old Col-0, *dhs1*, *dhs2* and *dhs3* on the media without any AAAs or with Tyr or Trp at 100 µM. Positions of their root tips are indicated by yellow lines. White scale bars = 1 cm. **(B)** Root length measurement for 10-day-old Col-0, *dhs1*, *dhs2*, and *dhs3* grown on plates containing Phe, Tyr, Trp, and shikimate at 0-1000 µM. ****$P \leq 0.0001$ denotes significant differences by one-way ANOVA against the corresponding Col-0 samples. Data are means ± SD ($n > 10$ replicated samples). All the individual data points are shown as dots.

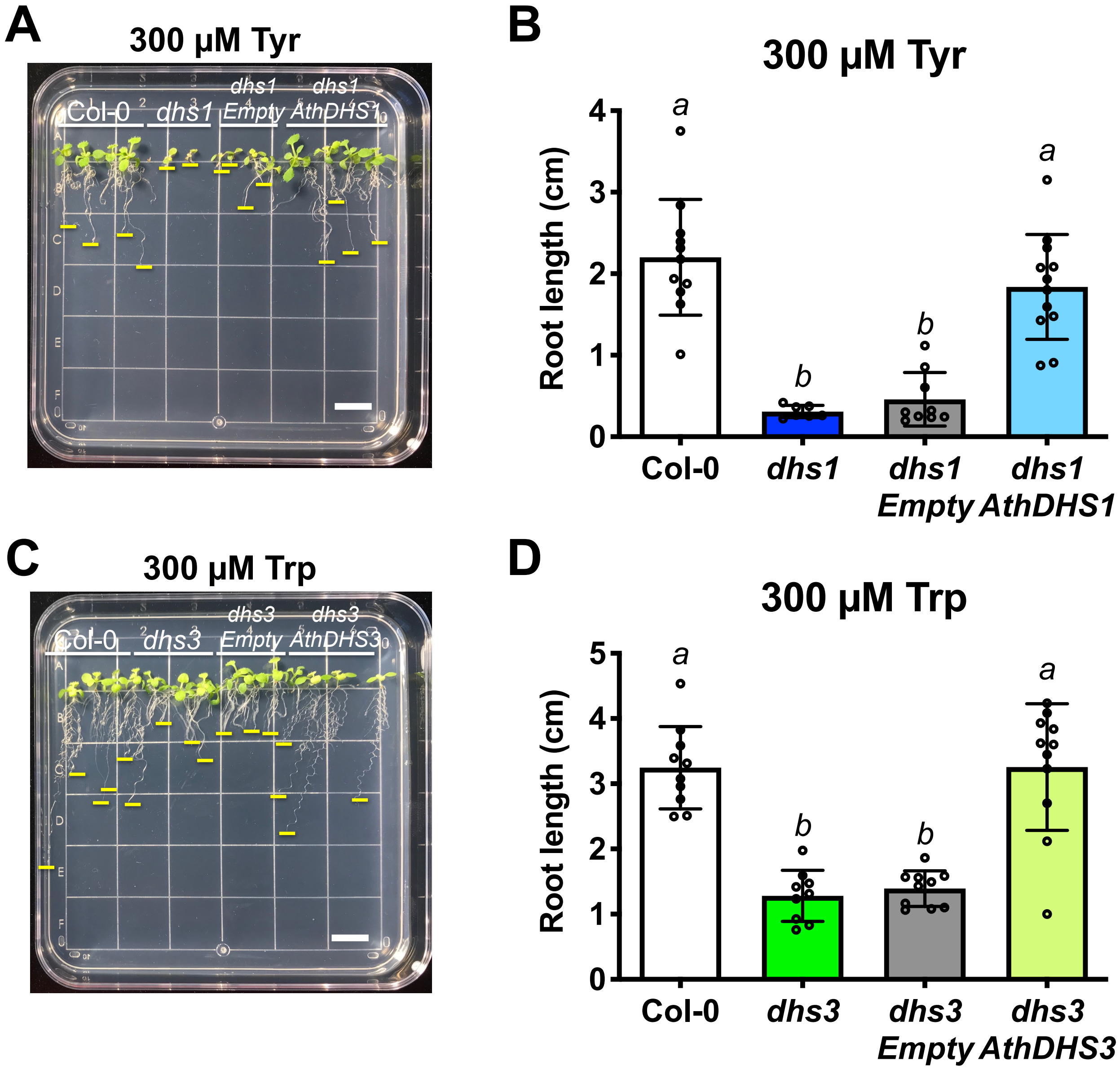

Supplemental Figure 11. (supports Figure 7) Complementation of *dhs1* and *dhs3* rescued their hypersensitivity to Tyr and Trp, respectively. **(A, B)** Root growth picture (A) and length measurement (B) of 10-day-old Col-0, *dhs1* and T1 generations of *dhs1* having empty vector or expressing *AthDHS1* on the media containing Tyr at 300 μM. **(C, D)** Root growth picture (D) and length measurement (D) of 10-day-old Col-0, *dhs3* and T1 generations of *dhs3* having empty vector or expressing *AthDHS3* on the media containing Trp at 300 μM. Positions of their root tips are indicated by yellow lines. While scale bars = 1 cm. Different letters indicate statistically significant differences between samples (two-way ANOVA, $P<0.05$). Data are means ± SD ($n > 9$ replicated sample). All the individual data points are shown as dots.

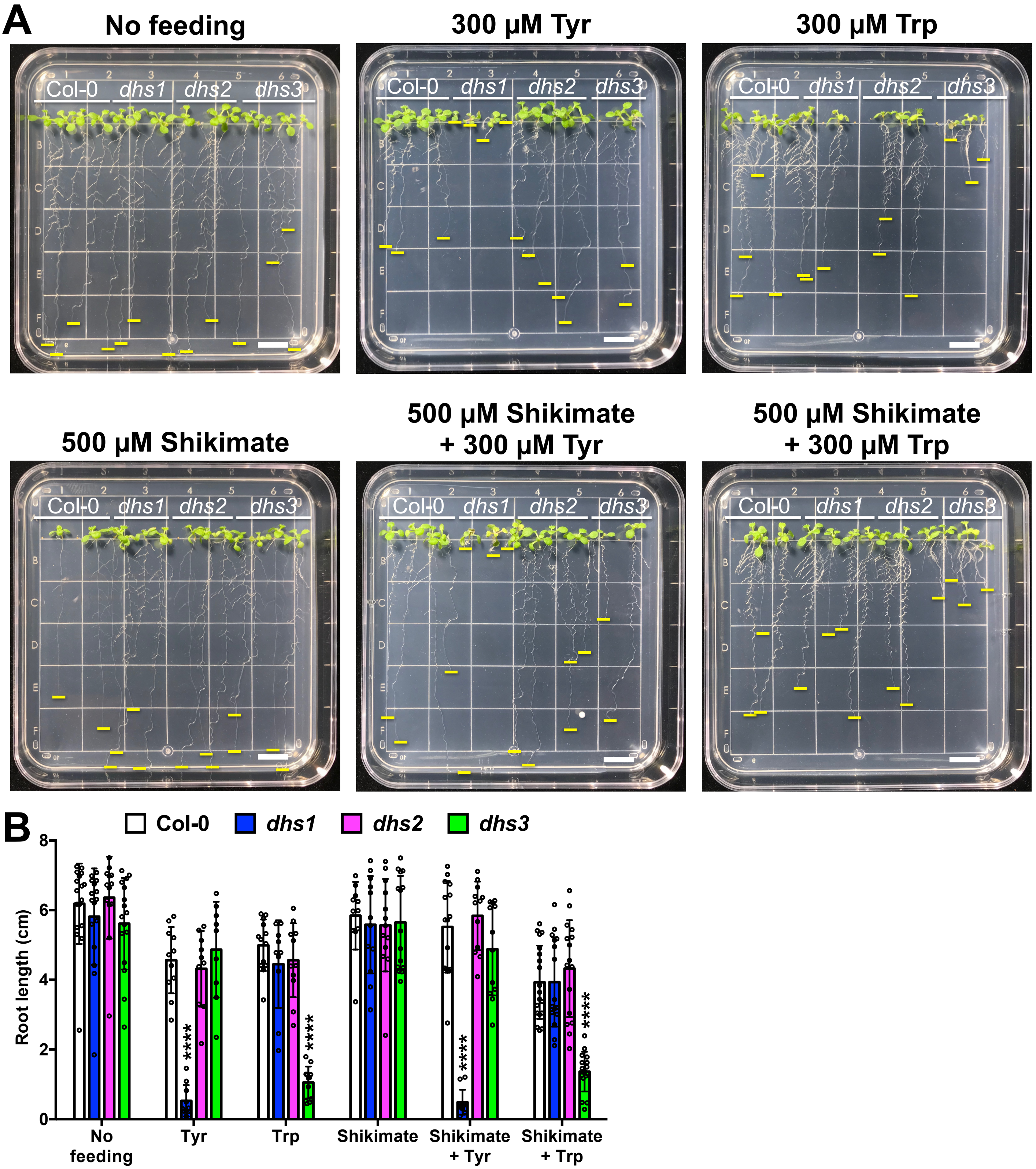

Supplemental Figure 12. (supports Figure 7) Col-0 and the *dhs* mutants grown with AAA and/or shikimate. **(A)** Root growth pictures of 10-day-old Col-0, *dhs1*, *dhs2* and *dhs3* on the media containing Tyr or Trp at 300 µM with or without shikimate at 500 µM. Positions of their root tips are indicated by yellow lines. While scale bars = 1 cm. **(B)** Root length measurement for 10-day-old Col-0, *dhs1*, *dhs2* and *dhs3* grown on plates containing Tyr or Trp with or without shikimate. ****$P \leq 0.0001$ denotes significant differences by one-way ANOVA against the corresponding "Col-0" samples. Data are means ± SD ($n > 10$ replicated samples). All the individual data points are shown as dots.

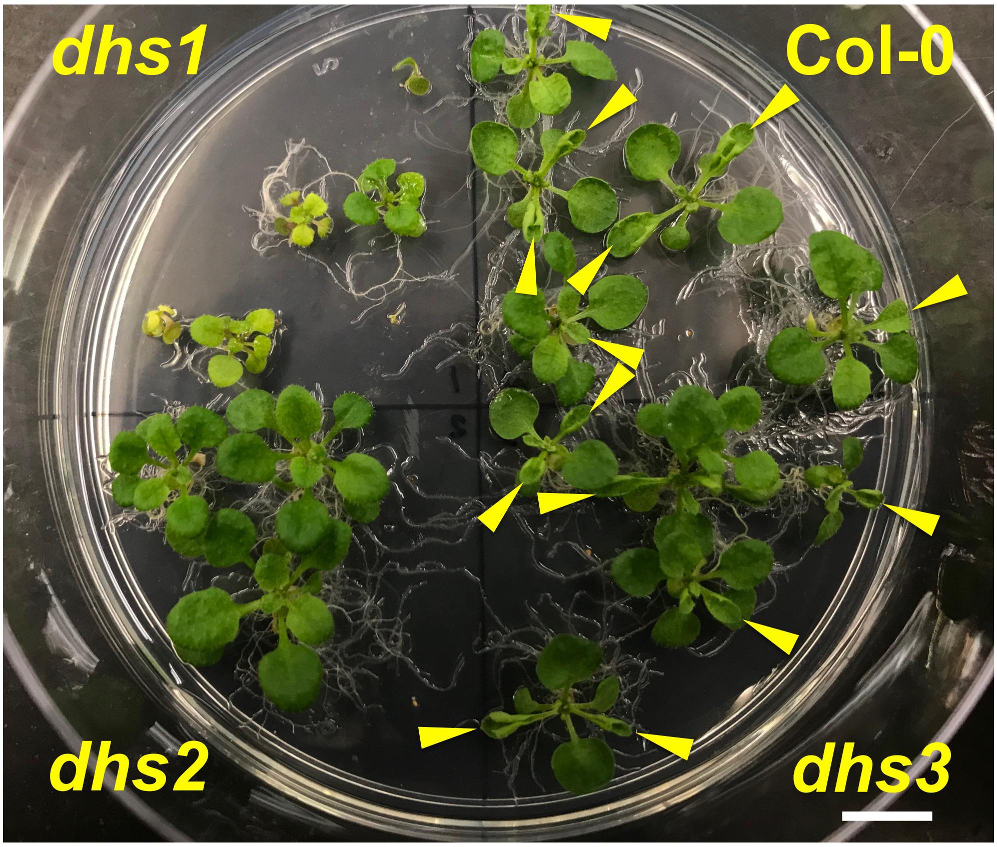

Supplemental Figure 13. (supports Figure 8) A growth picture of 14-day-old Col-0 and the *dhs* mutants in the presence of extra Tyr. Plant growth picture of 14-day-old Col-0, *dhs1*, *dhs2* and *dhs3* grown in the presence of Tyr at 300 μM. Curly cup-shaped leave are indicated by yellow arrows. While scale bars = 1cm.

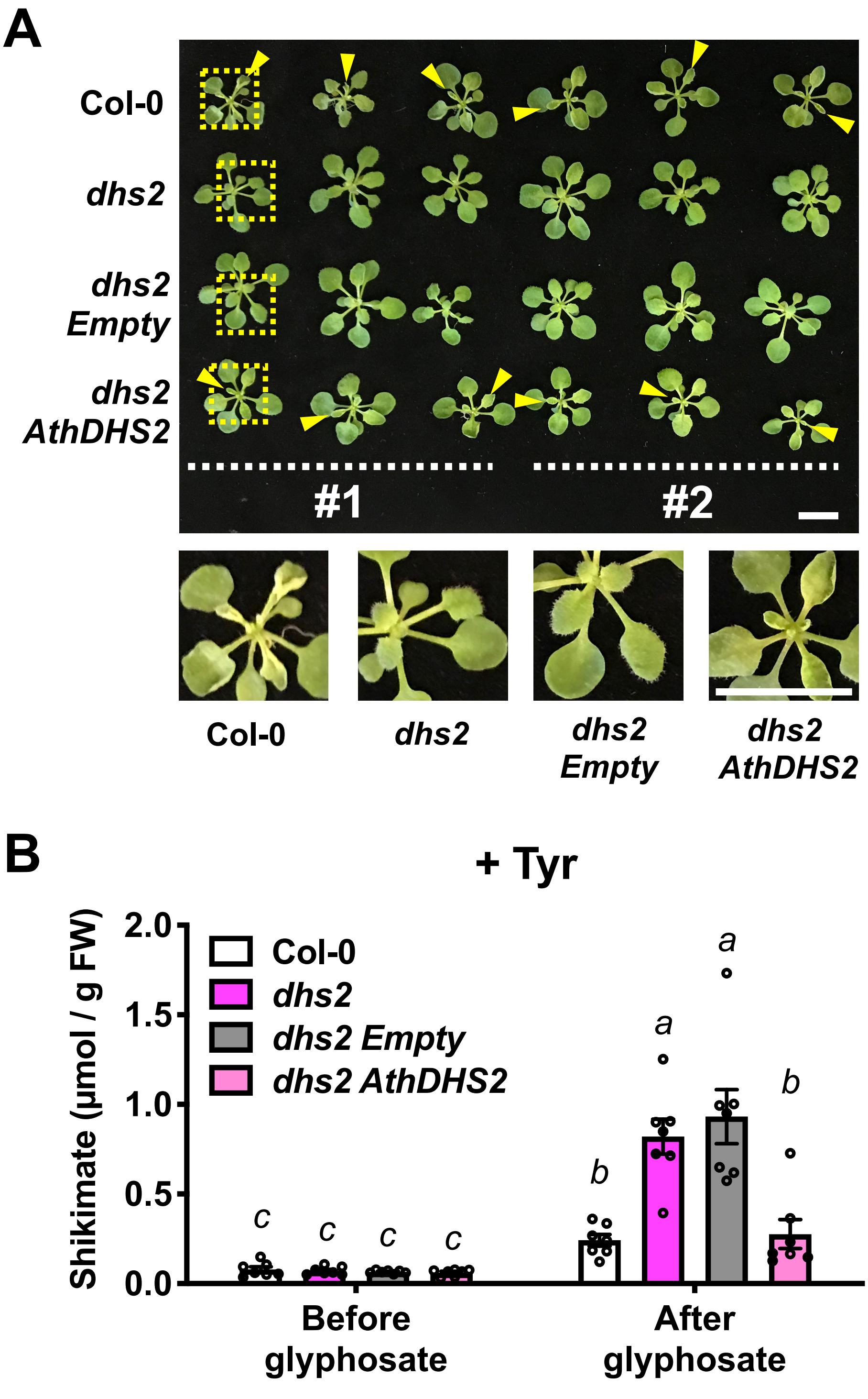

Supplemental Figure 14. (supports Figure 8) Complementation of *dhs2* rescued the phenotypes of the leaf development and the shikimate accumulation under high Tyr condition. **(A)** Plant growth picture of 14-day-old Col-0, *dhs2*, two independent T2 generation lines of *dhs2* having empty vector or expressing *AthDHS2* grown in the presence of Tyr at 300 µM. Curly cup-shaped leave are indicated by yellow arrows. While scale bars = 1cm. **(B)** Shikimate contents of Col-0, *dhs2*, and Col-0, *dhs2* and T2 generations of *dhs2* having empty vector or expressing *AthDHS2* grown on the media with Tyr at 300 µM, before and 2 days after treatment of 250 µM glyphosate. Different letters indicate statistically significant differences between samples (two-way ANOVA, $P<0.05$). Data are means ± SEM (n = 7 replicated samples). All the individual data points are shown as dots.

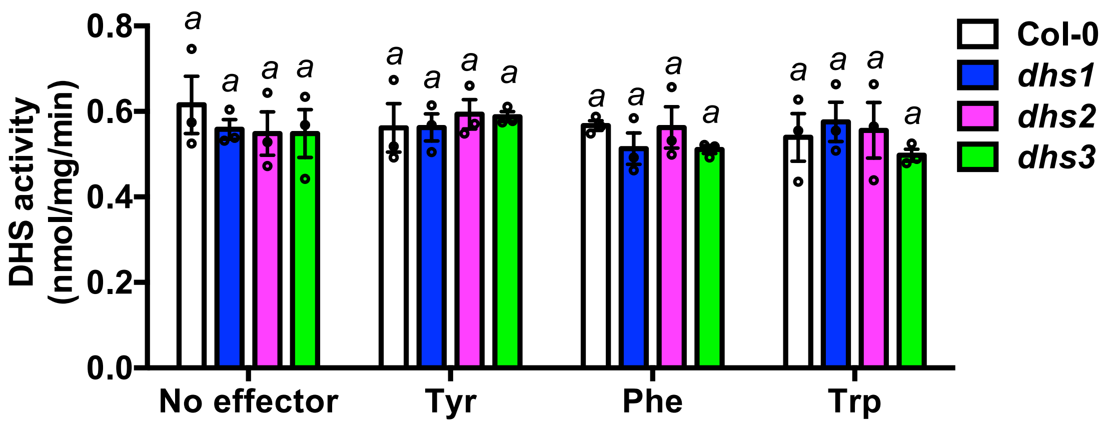

Supplemental Figure 15. (supports Figure 5) Enzymatic assay of crude extracts from Col-0 and the *dhs* mutants in the presence of individual AAA. DHS activity assay of crude extracts isolated from 4-week-old mature leaves of Col-0, *dhs1*, *dhs2* and *dhs3* was conducted in the presence of individual AAAs at 1mM. Different letters indicate statistically significant differences between samples (two-way ANOVA, $P<0.05$). Data are means ± SEM (n = 3 replicated samples). All the individual data points are shown as dots.

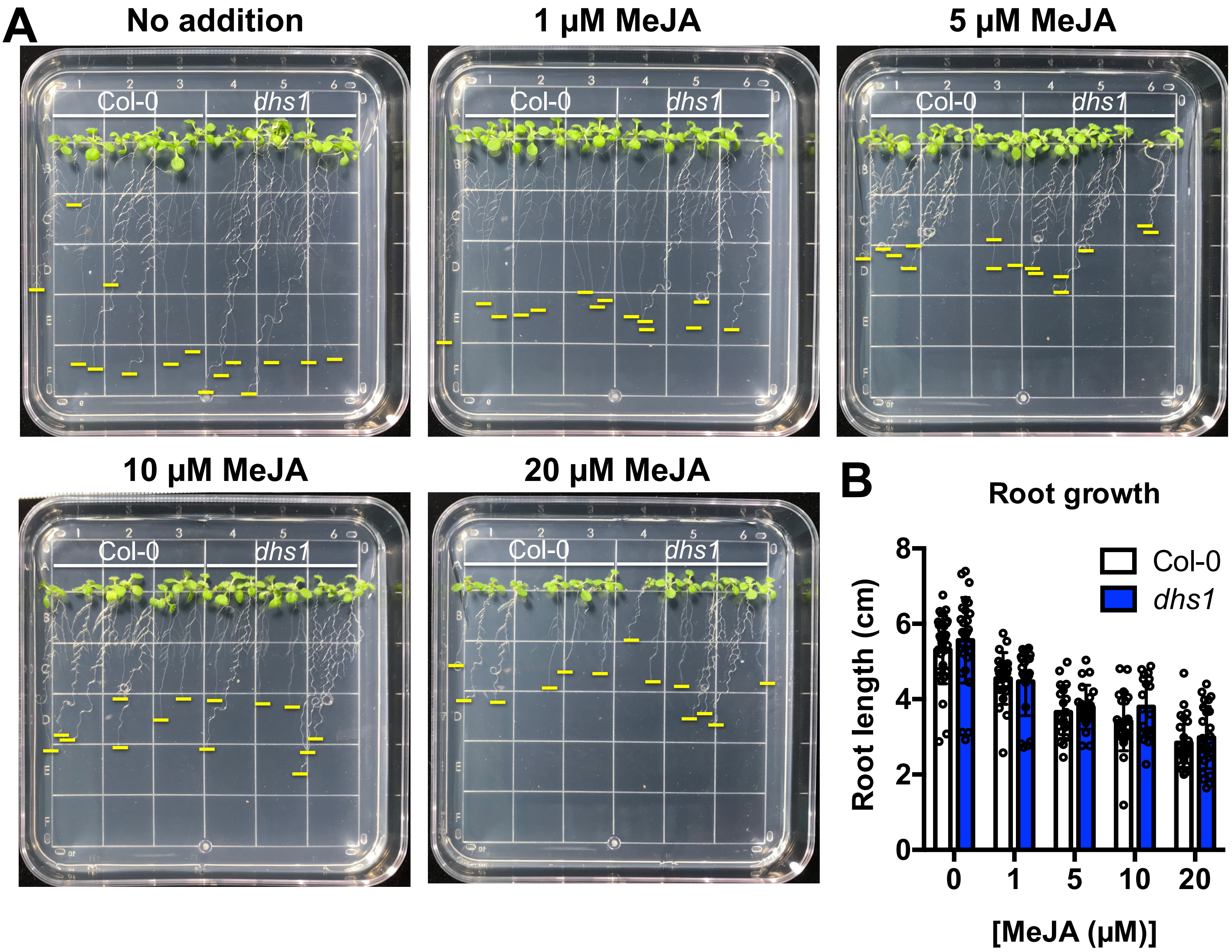

Supplemental Figure 16. (supports Figure 9) Phenotypes of Col-0 and *dhs1* grown with MeJA **(A, B)** Root growth picture (A) and length measurement (B) of 10-day-old Col-0 and *dhs1* on the media containing MeJA at 0, 1, 5 10 and 20 µM. Positions of their root tips are indicated by yellow lines. While scale bars = 1 cm. **(C)** Accumulation of Trp, I3M, 4MOI3M, 1MOI3M, 4MSOB, 5MSOP, 4MTB, 8MSOO, 7MTH, sinapoyl-*O*-glucoside, sinapoyl-malate, Q3GR7R, K3GR7R, Q3G7R, K3G7R, Q3R7R and K3R7R in 10-day-old Col-0 and *dhs1* on the media containing MeJA at 0, 1, 5 10 and 20 µM. $^{*}P \leq 0.05$ and $^{**}P \leq 0.01$ denote significant differences by Student`s *t*-test against the corresponding "Col-0" samples. Data are means ± SEM (n = 3 replicated sample). All the individual data points are shown as dots. I3M, indolyl-3-methyl glucosinolate; 4MOI3M, 4-methoxy-indol-3-ylmethyl glucosinolate; 1MOI3M, 1-methoxy-3-indolylmethyl glucosinolate; 4MSOB, 4-methylsulfinylbutyl glucosinolate; 5MSOP, 5-methylsulfinylpentyl glucosinolate; 4MTB, 4-methylthiobutyl glucosinolate; 8MSOO, 8-methylsulfinyloctyl glucosinolate; 7MTH, 7-methylthioheptyl glucosinolate; Q3GR7R, quercetin-3-*O*-(2"-*O*-rhamnosyl)glucoside-7-*O*-rhamnoside; K3GR7R, kaempferol-3-*O*-(2"-*O*-rhamnosyl)glucoside-7-*O*-rhamnoside; Q3G7R, quercetin-3-*O*-glucoside-7-*O*-rhamnoside; K3G7R, kaempferol-3-*O*-glucoside-7-*O*-rhamnoside; Q3R7R, quercetin-3-*O*-rhamoside-7-*O*-rhamnoside; K3R7R, keampferol-3-*O*-rhamnoside-7-*O*-rhamnoside.

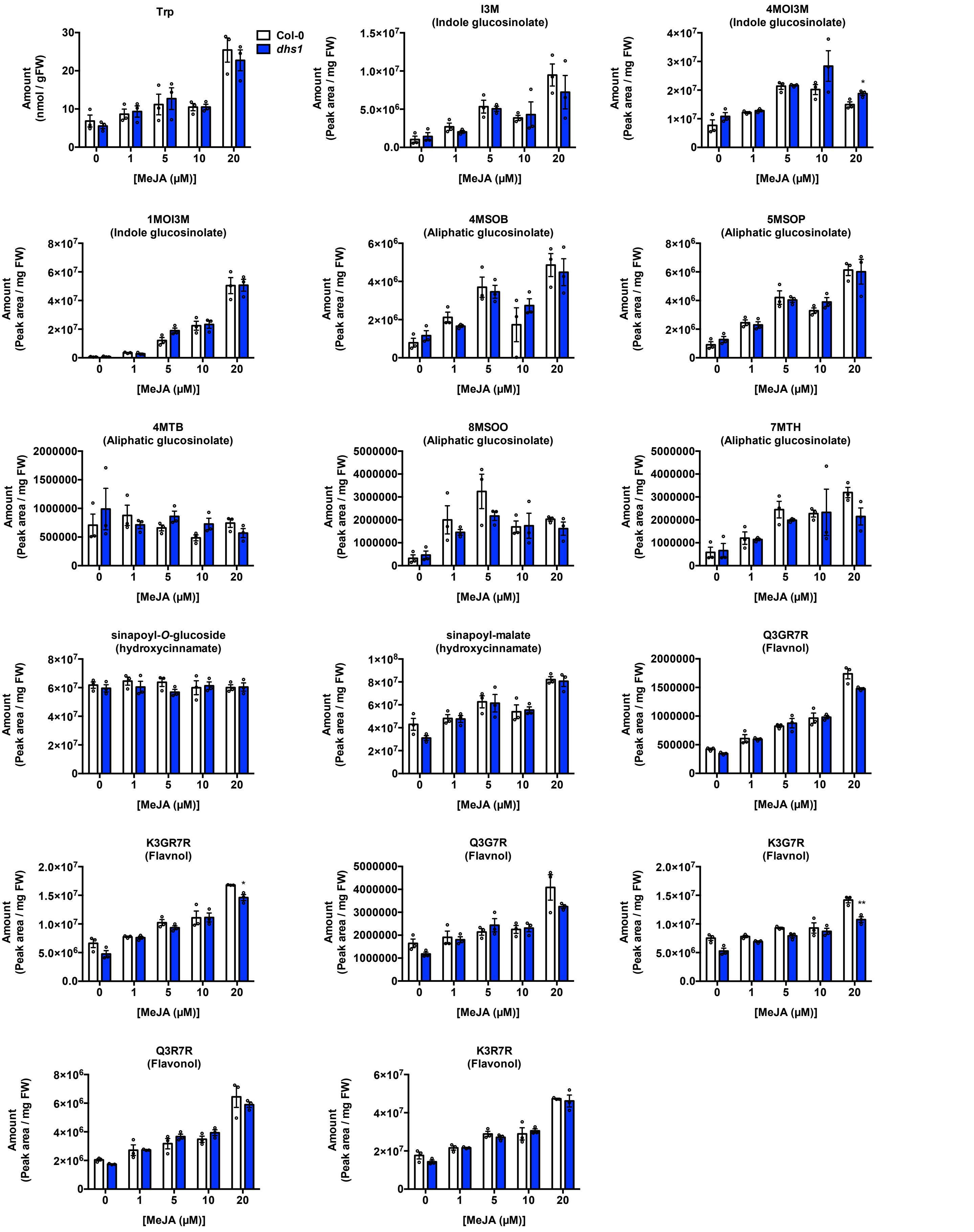

Supplemental Figure 16. (continued)

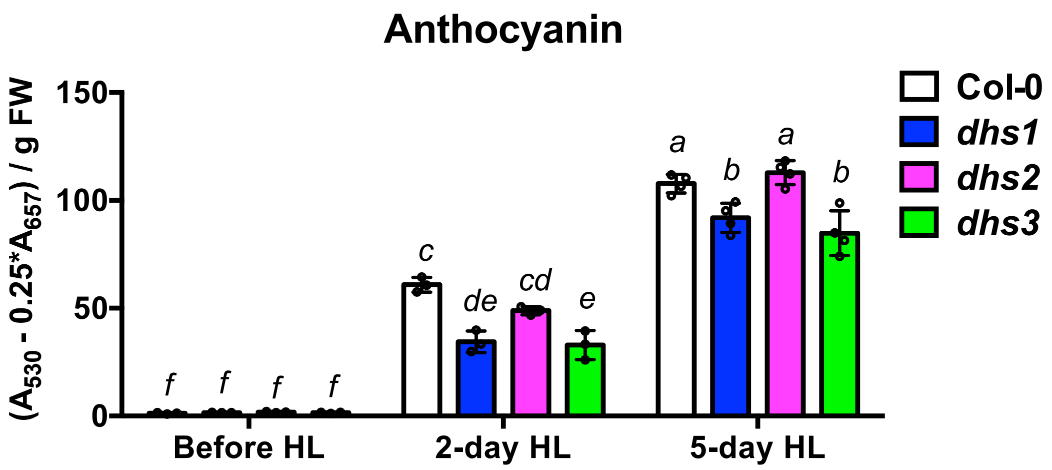

Supplemental Figure 17. (supports Figure 9) Accumulation of anthocyanins before and 2 and 5 days after HL treatment. Accumulation of anthocyanins in Col-0, *dhs1*, *dhs2* and *dhs3* before and 2 and 5 days after treatment of continuous HL. Different letters indicate statistically significant differences between samples (two-way ANOVA, $P<0.05$). Data are means ± SEM (n = 3-4 replicated samples). All the individual data points are shown as dots.

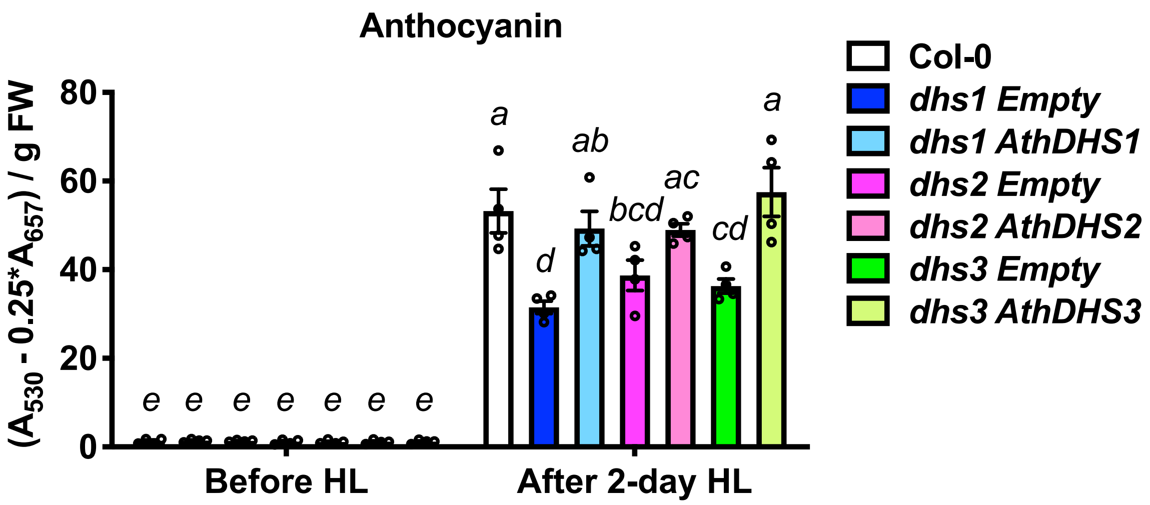

Supplemental Figure 18. (supports Figure 9) Anthocyanin accumulation of the *dhs* complementation lines before and 2 and 5 days after HL treatment. Accumulation of anthocyanins in Col-0 and T2 generations of each *dhs* mutant having empty vector or expressing corresponding AthDHS isoforms before and 2 and 5 days after treatment of continuous HL. Different letters indicate statistically significant differences between samples (two-way ANOVA, $P<0.05$). Data are means ± SEM (n = 4 replicated samples). All the individual data points are shown as dots.

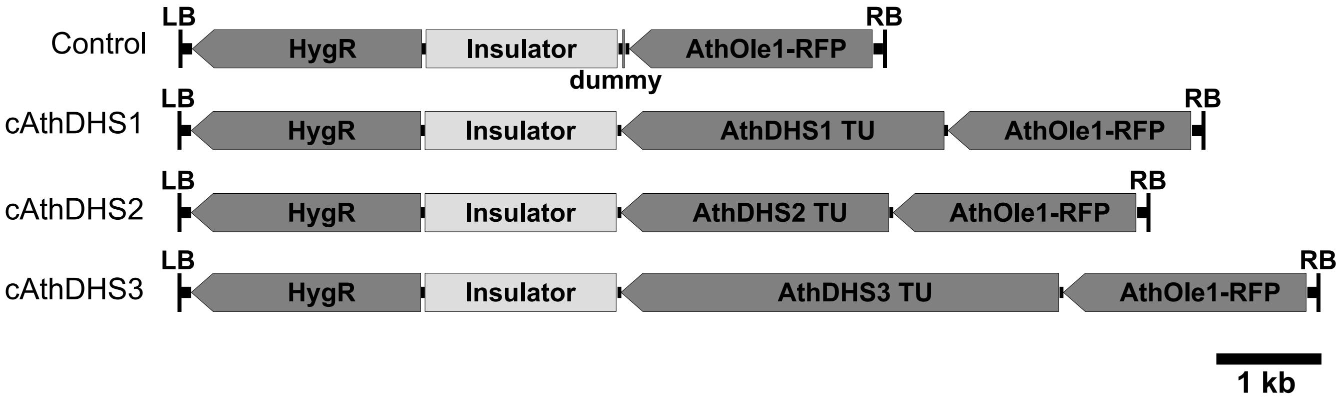

Supplemental Figure 19. (supports Figures 7 to 9) Schematic diagrams of *AthDHS* gene constructs for complementation tests. Each transcriptional unit (TU) contains promotor, 5`- and 3`-UTRs, CDS or genomic sequences of each *AthDHS* gene. HygR and Insulator regions represent Hygromycin resistance sequence and transformation booster sequence, respectively. For the control vector, the dummy TU was swapped with the AthDHS TU. Bar: 1 kb.

***AthDHS1* coexpression list**			
	Locus	Alias	Function
0	At4g39980	DHS1	3-deoxy-D-arabino-heptulosonate 7-phosphate synthase 1
1	At3g54640	TSA1	tryptophan synthase alpha chain
2	At4g35630	PSAT	phosphoserine aminotransferase
3	At2g04400	Aldolase-type TIM barrel	Aldolase-type TIM barrel family protein
4	At5g05730	WEI2	anthranilate synthase alpha subunit 1
5	At5g01500	TAAC	thylakoid ATP/ADP carrier
6	At4g34200	EDA9	D-3-phosphoglycerate dehydrogenase
7	At3g08640	DUF3411	Protein of unknown function (DUF3411)
8	At4g31500	SUR2	cytochrome P450, family 83, subfamily B, polypeptide 1
9	At4g39950	CYP79B2	cytochrome P450, family 79, subfamily B, polypeptide 2
10	At1g22410	DHS3	Class-II DAHP synthetase family protein
11	At1g74100	SOT16	sulfotransferase 16
12	At5g17990	TRP1	tryptophan biosynthesis 1
13	At5g63980	SUPO1	Inositol monophosphatase family protein
14	At1g48850	CS	chorismate synthase, putative / 5-enolpyruvylshikimate-3-phosphate phospholyase, putative
15	At2g22250	PAT	prephenate aminotransferase
16	At2g30860	GSTF9	glutathione S-transferase PHI 9
17	At5g66120	DHQS	3-dehydroquinate synthase, putative
18	At3g51450	phosphotriesterase	Calcium-dependent phosphotriesterase superfamily protein
19	At1g76790	IGMT5	O-methyltransferase family protein
20	At2g20610	SUR1	Tyrosine transaminase family protein

***AthDHS2* coexpression list**			
	Locus	Alias	Function
0	At4g33510	DHS2	3-deoxy-d-arabino-heptulosonate 7-phosphate synthase
1	At3g26580	Tetratricopeptide repeat (TPR)-like	Tetratricopeptide repeat (TPR)-like superfamily protein
2	At5g05200	kinase	Protein kinase superfamily protein
3	At1g63770	Peptidase M1	Peptidase M1 family protein
4	At4g27800	TAP38	thylakoid-associated phosphatase 38
5	At1g80480	PTAC17	plastid transcriptionally active 17
6	At5g19540	DY1	unknown
7	At1g21680	DPP6 N-terminal domain-like	DPP6 N-terminal domain-like protein
8	At4g13250	NYC1	NAD(P)-binding Rossmann-fold superfamily protein
9	At3g19490	NHD1	sodium:hydrogen antiporter 1
10	At1g06460	alpha-crystallin domain 32.1	alpha-crystallin domain 32.1
11	At3g60190	EDR3	DYNAMIN-like 1E
12	At4g25650	TIC55-IV	ACD1-like
13	At5g26570	PWD	catalytics;carbohydrate kinases;phosphoglucan, water dikinases
14	At5g23240	N-terminal	DNAJ heat shock N-terminal domain-containing protein
15	At4g18240	SSIV	starch synthase 4
16	At1g20630	CAT1	catalase 1
17	At3g15840	PIFI	post-illumination chlorophyll fluorescence increase
18	At4g03520	ATHM2	Thioredoxin superfamily protein
19	At2g29630	THIC	thiaminC
20	At2g47390	oligopeptidase	Prolyl oligopeptidase family protein

***AthDHS3* coexpression list**			
	Locus	Alias	Function
0	At1g22410	DHS3	Class-II DAHP synthetase family protein
1	At1g48850	CS	chorismate synthase, putative / 5-enolpyruvylshikimate-3-phosphate phospholyase, putative
2	At2g37040	PAL1	PHE ammonia lyase 1
3	At4g34050	CCoAOMT1	S-adenosyl-L-methionine-dependent methyltransferases superfamily protein
4	At2g36880	MAT3	methionine adenosyltransferase 3
5	At1g28680	COSY	HXXXD-type acyl-transferase family protein
6	At2g30490	REF3	cinnamate-4-hydroxylase
7	At3g02360	dehydrogenase	6-phosphogluconate dehydrogenase family protein
8	At2g22250	PAT	prephenate aminotransferase
9	At5g66120	DHQS	3-dehydroquinate synthase, putative
10	At1g51680	4CL1	4-coumarate:CoA ligase 1
11	At5g54160	COMT1	O-methyltransferase 1
12	At2g45290	TKL2	Transketolase
13	At3g06350	DHQ/SDH	dehydroquinate dehydratase, putative / shikimate dehydrogenase, putative
14	At1g55210	dirigent-like	Disease resistance-responsive (dirigent-like protein) family protein
15	At3g21240	4CL2	4-coumarate:CoA ligase 2
16	At3g53260	PAL2	phenylalanine ammonia-lyase 2
17	At5g22630	ADT5	arogenate dehydratase 5
18	At3g21230	4CL5	4-coumarate:CoA ligase 5
19	At4g39980	DHS1	3-deoxy-D-arabino-heptulosonate 7-phosphate synthase 1
20	At5g24760	dehydrogenase	GroES-like zinc-binding dehydrogenase family protein

Supplemental Table 1. (supports Figure 5) A list of top 20 genes co-expressed with *AthDHS1*, *AthDHS2* and *AthDHS3*. Yellow color indicates genes in common steps of the shikimate and Tyr/Phe biosynthetic pathways, whereas purple, magenta and green colors represent genes specifically involved in metabolisms of Phe, Trp and serine, respectively. The list was obtained from Atted-II (Ohbayashi et al., 2018).

A

Standard growth condition

Metabolites	Genotype	**Col-0**		***dhs1***		***dhs2***		***dhs3***	
		nmol/g FW ± SEM		nmol/g FW ± SEM		nmol/g FW ± SEM		nmol/g FW ± SEM	
Amino acids	**Tyr**	6.38 ± 0.25	*c*	7.31 ± 0.32	*c*	6.54 ± 0.62	*c*	5.78 ± 0.69	*c*
	**Phe**	56.07 ± 4.42	*a*	32.08 ± 1.22	*b*	59.60 ± 3.92	*a*	25.95 ± 2.79	*bc*
	**Trp**	3.94 ± 0.05	*b*	5.00 ± 1.08	*b*	3.45 ± 0.14	*b*	3.22 ± 0.20	*b*
	**Ala**	815.06 ± 51.27	*ab*	704.65 ± 25.08	*ac*	764.59 ± 53.81	*ac*	514.05 ± 60.71	*c*
	**Gly**	23.71 ± 3.62	*ab*	18.88 ± 1.87	*ab*	16.94 ± 2.21	*ab*	15.31 ± 2.57	*b*
	**Val**	73.83 ± 5.68	*b*	83.80 ± 3.23	*b*	85.80 ± 4.49	*b*	82.06 ± 8.68	*b*
	**Leu**	22.59 ± 1.38	*b*	23.63 ± 0.59	*b*	25.42 ± 1.03	*b*	21.24 ± 1.99	*b*
	**Ile**	16.16 ± 2.53	*bc*	16.73 ± 1.18	*bc*	14.58 ± 0.49	*c*	18.77 ± 1.99	*bc*
	**Met**	12.35 ± 0.99	*ab*	13.71 ± 1.38	*a*	11.27 ± 1.01	*ab*	10.52 ± 1.46	*ab*
	**Ser**	431.26 ± 20.79	*a*	427.30 ± 18.65	*a*	374.92 ± 17.38	*ab*	360.10 ± 40.53	*ac*
	**Thr**	1365.48 ± 51.61	*a*	1426.36 ± 85.07	*a*	1136.94 ± 90.83	*a*	1175.73 ± 128.79	*a*
	**Asp**	67.73 ± 2.19	*b*	115.68 ± 11.66	*a*	73.03 ± 8.70	*b*	72.10 ± 10.76	*b*
	**Glu**	927.57 ± 40.41	*b*	1497.28 ± 107.53	*a*	1155.71 ± 134.14	*ab*	908.94 ± 114.72	*bc*
		Peak area/g FW ± SEM		Peak area/g FW ± SEM		Peak area/g FW ± SEM		Peak area/g FW ± SEM	
Indole glucosinolates	**I3M**	1618186 ± 267761	*b*	2162689 ± 38864	*b*	1312914 ± 86598	*b*	1012398 ± 124437	*b*
	**4MOI3M**	1447161 ± 124714	*a*	1223702 ± 32236	*ab*	1365724 ± 64352	*a*	1139746 ± 97400	*ab*
	**1MOI3M**	44583 ± 19323	*bcd*	299524 ± 112183	*ad*	65584 ± 14060	*cd*	28130 ± 2880	*d*
Aliphatic glucosinolates	**4MSOB**	1374471 ± 167594	*b*	1399419 ± 126484	*b*	1080628 ± 110614	*b*	747007 ± 89356	*b*
	**5MSOP**	52128 ± 6315	*bc*	63047 ± 4702	*bc*	48750 ± 4055	*c*	33766 ± 3127	*c*
	**4MTB**	392473 ± 41968	*bc*	255356 ± 12932	*c*	268131 ± 32958	*c*	181865 ± 32482	*c*
	**8MSOO**	1221835 ± 200488	*bc*	1020855 ± 96866	*bc*	945455 ± 178803	*c*	642410 ± 67457	*c*
	**7MTH**	116259 ± 14668	*d*	120092 ± 9605	*d*	118274 ± 5322	*d*	77456 ± 10709	*d*
Hydroxy-cinnamates	**sinapoyl-*O*-glucoside**	354403 ± 47894	*b*	386394 ± 101111	*b*	319219 ± 46524	*b*	255698 ± 23177	*b*
	**sinapoyl-malate**	13943492 ± 183399	*a*	14293537 ± 856925	*a*	13193179 ± 404423	*ab*	13119429 ± 328192	*a*
Flavonols	**Q3GR7R**	38808 ± 3397	*c*	44556 ± 10549	*c*	33591 ± 2656	*c*	37100 ± 4014	*c*
	**K3GR7R**	1378912 ± 171514	*b*	1482260 ± 273312	*b*	1122329 ± 65265	*b*	1445473 ± 160272	*b*
	**Q3G7R**	49451 ± 4308	*c*	48579 ± 7491	*c*	42368 ± 3253	*c*	47249 ± 4844	*c*
	**K3G7R**	1182305 ± 206183	*b*	997365 ± 164241	*b*	995291 ± 92415	*b*	1257797 ± 148460	*b*
	**Q3R7R**	77643 ± 9931	*c*	74257 ± 17871	*c*	63473 ± 5756	*c*	70594 ± 8402	*c*
	**K3R7R**	4036865 ± 567354	*b*	4398276 ± 735220	*b*	3314500 ± 209432	*b*	4382536 ± 443836	*b*
		nmol/g FW ± SEM		nmol/g FW ± SEM		nmol/g FW ± SEM		nmol/g FW ± SEM	
Tocopherols	**alpha-tocopherol**	15.70 ± 1.05	*c*	16.20 ± 1.13	*c*	18.08 ± 1.26	*c*	15.75 ± 1.13	*c*
	**gamma-tocopherol**	0.85 ± 0.11	*b*	0.89 ± 0.14	*b*	1.56 ± 0.22	*b*	0.87 ± 0.16	*b*

Supplemental Table 2. (supports Figure 9) (A-B) Levels of amino acids and AAA-derived metabolites under standard growth condition (before HL treatment) (A) and after 2-day HL treatment (B) in Col-0 and the *dhs* mutants. Different letters indicate statistically significant differences between the samples before and after HL treatment(two-way ANOVA, $P<0.05$). Data are means ± SEM (n = 4-6 replicated sample). I3M, indolyl-3-methyl glucosinolate; 4MOI3M, 4-methoxy-indol-3-ylmethyl glucosinolate; 1MOI3M, 1-methoxy-3-indolylmethyl glucosinolate; 4MSOB, 4-methylsulfinylbutyl glucosinolate; 5MSOP, 5-methylsulfinylpentyl glucosinolate; 4MTB, 4-methylthiobutyl glucosinolate; 8MSOO, 8-methylsulfinyloctyl glucosinolate; 7MTH, 7-methylthioheptyl glucosinolate; Q3GR7R, quercetin-3-*O*-(2"-*O*-rhamnosyl)glucoside-7-*O*-rhamnoside; K3GR7R, kaempferol-3-*O*-(2"-*O*-rhamnosyl)glucoside-7-*O*-rhamnoside; Q3G7R, quercetin-3-*O*-glucoside-7-*O*-rhamnoside; K3G7R, kaempferol-3-*O*-glucoside-7-*O*-rhamnoside; Q3R7R, quercetin-3-*O*-rhamoside-7-*O*-rhamnoside; K3R7R, keampferol-3-*O*-rhamnoside-7-*O*-rhamnoside.

B

2 days after HL

Metabolites	Genotype	Col-0	*dhs1*	*dhs2*	*dhs3*
		nmol/g FW ± SEM	nmol/g FW ± SEM	nmol/g FW ± SEM	nmol/g FW ± SEM
Amino acids	**Tyr**	37.14 ± 1.55 ^{b}	44.28 ± 1.15 ^{a}	40.67 ± 2.50 ^{ab}	34.55 ± 2.13 ^{b}
	**Phe**	20.12 ± 1.37 ^{c}	16.99 ± 0.99 ^{c}	22.75 ± 1.18 ^{bc}	19.73 ± 2.39 ^{c}
	**Trp**	827.47 ± 106.76 ^{ab}	562.46 ± 30.42 ^{bc}	724.28 ± 61.41 ^{ac}	863.04 ± 96.02 ^{a}
	**Ala**	33.23 ± 5.71 ^{ab}	23.74 ± 2.89 ^{ab}	18.82 ± 2.20 ^{ab}	29.36 ± 5.67 ^{ab}
	**Gly**	103.43 ± 9.13 ^{ab}	97.48 ± 2.67 ^{ab}	103.67 ± 5.42 ^{ab}	120.23 ± 10.00 ^{a}
	**Val**	104.18 ± 7.67 ^{a}	100.80 ± 4.96 ^{a}	100.21 ± 3.60 ^{a}	99.39 ± 7.45 ^{a}
	**Leu**	35.01 ± 3.21 ^{a}	26.37 ± 1.00 ^{ab}	31.41 ± 2.83 ^{a}	33.71 ± 3.99 ^{a}
	**Ile**	9.31 ± 1.09 ^{ab}	8.64 ± 0.44 ^{b}	10.10 ± 0.79 ^{ab}	11.58 ± 1.08 ^{ab}
	**Met**	266.46 ± 23.31 ^{bcd}	222.10 ± 9.93 ^{d}	254.56 ± 19.62 ^{cd}	304.80 ± 32.99 ^{bcd}
	**Ser**	295.67 ± 31.58 ^{b}	309.59 ± 18.92 ^{b}	378.28 ± 32.02 ^{b}	600.65 ± 74.96 ^{b}
	**Thr**	26.22 ± 3.15 ^{c}	26.34 ± 3.08 ^{c}	33.49 ± 4.45 ^{c}	51.32 ± 9.36 ^{bc}
	**Asp**	441.57 ± 43.61 ^{d}	341.53 ± 44.72 ^{d}	415.85 ± 50.51 ^{d}	545.71 ± 70.01 ^{cd}
	**Glu**	15.35 ± 0.66 ^{a}	19.00 ± 1.26 ^{a}	18.21 ± 1.34 ^{a}	16.41 ± 1.20 ^{a}
		Peak area/g FW ± SEM	Peak area/g FW ± SEM	Peak area/g FW ± SEM	Peak area/g FW ± SEM
Indole glucosinolates	**I3M**	11181179 ± 457469 ^{a}	11929274 ± 837534 ^{a}	11675304 ± 298370 ^{a}	12917040 ± 502593 ^{a}
	**4MOI3M**	1045075 ± 53667 ^{b}	1086290 ± 67077 ^{ab}	1094815 ± 66491 ^{ab}	1147134 ± 47656 ^{ab}
	**1MOI3M**	251694 ± 53105 ^{ad}	325086 ± 50559 ^{ab}	432187 ± 101053 ^{a}	276442 ± 44803 ^{ac}
Aliphatic glucosinolates	**4MSOB**	24688341 ± 5610219 ^{a}	14535906 ± 2816313 ^{ab}	23745653 ± 8750793 ^{a}	13516306 ± 2175628 ^{ab}
	**5MSOP**	846118 ± 137024 ^{a}	628449 ± 141773 ^{ab}	771807 ± 204436 ^{a}	505007 ± 68075 ^{ac}
	**4MTB**	1049700 ± 153121 ^{ab}	1188444 ± 178090 ^{a}	1148064 ± 100920 ^{ab}	1624143 ± 215260 ^{a}
	**8MSOO**	2420124 ± 392629 ^{ab}	2193294 ± 388230 ^{ab}	1553168 ± 165547 ^{bc}	2804771 ± 219815 ^{a}
	**7MTH**	189344 ± 14704 ^{cd}	343067 ± 49266 ^{ab}	314200 ± 24145 ^{bc}	450663 ± 40851 ^{a}
Hydroxy-cinnamates	**sinapoyl-*O*-glucoside**	2964397 ± 463179 ^{a}	2505292 ± 347097 ^{a}	2359976 ± 130441 ^{a}	2015229 ± 312938 ^{a}
	**sinapoyl-malate**	10906858 ± 666408 ^{bc}	8972563 ± 317208 ^{c}	9698821 ± 422104 ^{c}	10197973 ± 651865 ^{c}
Flavonols	**Q3GR7R**	5618178 ± 408841 ^{a}	3934590 ± 340935 ^{b}	4068333 ± 132197 ^{b}	5049371 ± 370247 ^{b}
	**K3GR7R**	11152738 ± 731655 ^{a}	10864219 ± 564913 ^{a}	11772065 ± 1194302 ^{a}	10703426 ± 790953 ^{a}
	**Q3G7R**	10829136 ± 821005 ^{a}	6338270 ± 767078 ^{b}	5935996 ± 339276 ^{b}	7680406 ± 721513 ^{b}
	**K3G7R**	15431925 ± 1918828 ^{a}	11701847 ± 852344 ^{a}	11794314 ± 1580064 ^{a}	12108454 ± 1053952 ^{a}
	**Q3R7R**	14464128 ± 1010395 ^{a}	8534176 ± 1307941 ^{b}	8912169 ± 834605 ^{b}	11897659 ± 1248331 ^{b}
	**K3R7R**	29292571 ± 3970657 ^{a}	23957423 ± 1888758 ^{a}	28490591 ± 5823856 ^{a}	23660037 ± 2485650 ^{a}
		nmol/g FW ± SEM	nmol/g FW ± SEM	nmol/g FW ± SEM	nmol/g FW ± SEM
Tocopherols	**alpha-tocopherol**	152.78 ± 5.81 ^{ab}	151.66 ± 6.57 ^{sb}	169.99 ± 6.87 ^{a}	138.95 ± 3.19 ^{b}
	**gamma-tocopherol**	42.85 ± 2.65 ^{a}	42.95 ± 2.39 ^{a}	52.39 ± 2.97 ^{a}	35.39 ± 2.06 ^{a}

Supplemental Table 2. (continued)

Sequence	Aim	Target
CGCGCGGCAGCCATATGGTACACGCGGCTGAGCC	Cloning into pET28a	*AthDHS1*
GACGGAGCTCGAATTCTCAAGACACACGCTGGCTA	Cloning into pET28a	*AthDHS1*
CGCGCGGCAGCCATATGGTTCATTCGACTGAC	Cloning into pET28a	*AthDHS2*
TCGAATTCGGATCCTTAGACTCCAATAGATGAC	Cloning into pET28a	*AthDHS2*
CGCGCGGCAGCCATATGGCGTCGGCGGCGACGA	Cloning into pET28a	*AthDHS3*
GACGGAGCTCGAATTCTTAGAGGGCAAAAGCCTTTT	Cloning into pET28a	*AthDHS3*
GCGGAGAGCGTACCAGAC	qPCR	*AthDHS1*
GATCCATTTTGTTGCTCACCTT	qPCR	*AthDHS1*
CAATGCACGGAAACACAATC	qPCR	*AthDHS2*
ACGTCGAAGAACGCTCTCA	qPCR	*AthDHS2*
CAAGACTCGTCCCTTTGACG	qPCR	*AthDHS3*
GGGTGGCTACCTTCTTGCT	qPCR	*AthDHS3*
TCCTACTTCATGTAGCGCAGGAC	qPCR	*UBC9*
TCCTCCAGAATAAGGGCTATCCG	qPCR	*UBC9*
TTAAGGTTGGGAGGATGGC	RT-PCR	*AthDHS1*
TGTTTGGAATCAAAAAGTG	RT-PCR	*AthDHS1*
AATCTACCCAATCAGCCTC	RT-PCR	*AthDHS2*
AACTTAATAAGTAGAGATC	RT-PCR	*AthDHS2*
ATGATCAACCACCGTCAAC	RT-PCR	*AthDHS3*
TGGATCCATTTTATCACTC	RT-PCR	*AthDHS3*
ATACAAAGAGGTACAGCGAG	RT-PCR	*UBC21*
TTCTTAGGCATAGCGGCG	RT-PCR	*UBC21*
ATTTTGCCGATTTCGGAAC	Genotyping	SALK T-DNA
GATCAGGTGGGGAAGCTTAAC	Genotyping	*AthDHS1* left border
TTGTGTGTTTAGGTTGGGAGG	Genotyping	*AthDHS1* right border
GAAATCGAGGATTCCTGATCC	Genotyping	*AthDHS2* left border
TTACCCTGATTGCATCGAAAG	Genotyping	*AthDHS2* right border
ATACGACGGATCGTAATTTGTCG	Genotyping	SK T-DNA
CGACCTAATATCTTCAATTTCGAC	Genotyping	*AthDHS3* left border
GCCATTCTCCCAACCTAACA	Genotyping	*AthDHS3* right border

Supplemental Table 3. (supports Figures 5 and 6) Primer list used in this study.

Compound name	Abbreviation	Retention time (min)	*m/z*	associated *m/z* fragment	Peak annotation and reference
Tryptophan	Trp	6.63	203.08		chemical standard
Indolyl-3-methyl glucosinolate	I3M	7.93	447.05		chemical standard
4-methoxy-indol-3-ylmethyl glucosinolate	4MOI3M	11.50	477.06		Tohge et al., 2016
1-methoxy-3-indolylmethyl glucosinolate	1MOI3M	12.75	477.06		Tohge et al., 2016
4-methylsulfinylbutyl glucosinolate	4MSOB	1.32	436.04		Tohge et al., 2016
5-methylsulfinylpentyl glucosinolate	5MSOP	2.90	450.06		Tohge et al., 2016
4-methylthiobutyl glucosinolate	4MTB	6.98	420.05		Tohge et al., 2016
8-methylsulfinyloctyl glucosinolate	8MSOO	10.64	492.10		Tohge et al., 2016
7-methylthioheptyl glucosinolate	7MTH	13.71	462.09		Tohge et al., 2016
Sinapoyl-*O*-glucoside		11.87	385.11	223.06	Tohge et al., 2016
Sinapoyl-malate		13.35	339.07	223.06	Tohge et al., 2016
Quercetin-3-*O*-(2"-*O*-rhamnosyl)glucoside-7-*O*-rhamnoside	Q3GR7R	12.11	755.20	446, 608	Tohge et al., 2016
Kaempferol-3-*O*-(2"-*O*-rhamnosyl)glucoside-7-*O*-rhamnoside	K3GR7R	12.33	739.20	430.10, 593.15	Tohge et al., 2016
Quercetin-3-*O*-glucoside-7-*O*-rhamnoside	Q3G7R	12.60	609.14	446.09, 462.09	Tohge et al., 2016
Kaempferol-3-*O*-glucoside-7-*O*-rhamnoside	K3G7R	12.90	593.14	430.06, 447.09	Tohge et al., 2016
Quercetin-3-*O*-rhamoside-7-*O*-rhamnoside	Q3R7R	12.96	593.14	447.09	Tohge et al., 2016
Keampferol-3-*O*-rhamnoside-7-*O*-rhamnoside	K3R7R	13.27	577.15	430.10, 431.10	Tohge et al., 2016

Supplemental Table 4. (supports Figure 9) Summary of peak information obtained by LC-MS and LC-MS/MS analyses.
