# Supplementary material for: The entry reaction of the plant shikimate pathway is subjected to highly complex metabolite-mediated regulation
Source: Plant Cell. 2021 Jan 7;33(3):671–96. doi: 10.1093/plcell/koaa042 (PMC8136874; doi:10.1093/plcell/koaa042)
Supplement: koaa042_Supplementary_Data [file koaa042_supplementary_data.zip › tpc.00840.2020-s07.pdf]

The entry reaction of the plant shikimate pathway is subjected to highly-complex metabolite-mediated regulation

Ryo Yokoyama[1], Marcos V. V. de Oliveira[1], Bailey Kleven[1] and Hiroshi A. Maeda

Corresponding author: Hiroshi A. Maeda, maeda2@wisc.edu.

Review timeline:

**TPC2020-RA-00460**	Submission received:	June 12, 2020
	1st Decision:	July 27, 2020 *manuscript declined*
**TPC2020-RA-00840**	Submission received:	Oct. 7, 2020
	1st Decision:	Nov. 6, 2020 *accept with minor revision*
**TPC2020-RA-00840R1**	1st Revision received:	Nov. 18, 2020
	2nd Decision:	Nov. 19, 2020 *acceptance pending, sent to science editor*
	Final acceptance:	Dec. 16, 2020

REPORT: (The report shows the major requests for revision and author responses. Minor comments for revision and miscellaneous correspondence are not included. The original format may not be reflected in this compilation, but the reviewer comments and author responses are not edited, except to correct minor typographical or spelling errors that could be a source of ambiguity.)

TPC2020-RA-00460 1st Editorial decision – *declined* **July 27, 2020**

Thank you for choosing to send your manuscript entitled "The Entry Reaction of the Plant Shikimate Pathway Is Subjected to Highly-Complex Metabolite-Mediated Regulation" for consideration at The Plant Cell. Your submission has been evaluated by members of the editorial board as well as expert reviewers in your field, and we regret to inform you that we are not able to recommend publication of this manuscript in its current form. We have not made this decision lightly. We have had input from multiple scientists, and we have solicited post-review comments as well. Our present policy is to offer streamlined decisions and to not advise on the direction of the work by requesting extensive modifications or substantial additional experiments.

During the post-review consultation session, we also agreed that if you could address the major points raised by the reviewers by new experiments, we would welcome a resubmission. This may be treated as a new submission, but we would attempt to use at least some of the same reviewers. Nevertheless, reviewers will be asked to assess as a new manuscript (i.e. are the claims fully supported by the data and do the results presented move the field forward?), and not only whether previous reviewer comments have been addressed.

As you'll see when you read their comments, all three reviewers have provided careful and thorough reviews. Although we would like you to address each of their comments in your response to reviews, we felt that several items are of particular importance to improve your manuscript.

First, Reviewer 1 asks about the use of the term "young plants" and we agree that this is problematic because it conflates age, tissue type, and developmental state in a way that is confusing (e.g. some may be tempted to think about phase change in this context). Please consider how you can rephrase all of these results and discussion for clarity.

Second, two of the reviewers ask for more information on DHS isoform expression, suggesting that this information can be gleaned from publicly available databases. Adding this information would be straightforward and helpful.

Third, Reviewer 2 also suggests that DHS activity assays in the *dhs1* mutant background would provide a valuable "in vivo" test of your in vitro results. Completing these experiments should not delay a resubmission substantially. In contrast, although the analysis of double mutants might shed light on the questions you are asking and could be included in a revision if they are available, we do not want to delay your resubmission to the degree that their generation would require.

-- Reviewer comments:

Reviewer #1:

This study brings new findings in eukaryotes shikimate pathway regulation. It expands our understanding of the shikimate biosynthesis and the allosteric regulation by its intermediates, Tyr, Trp, and downstream products from the phenylpropanoids. It includes an extensive amount of biochemical and analytical chemistry analyses (in Arabidopsis and spinach) and used Arabidopsis mutants to understand the different regulation of each one of the DHS isozymes.

I have only minor comments:

Abstract:

Row 30: "are rarely inhibited by AAAs" - if this is the case, why it was studied in here? In row 32 you mention that "found that tyrosine and tryptophan inhibit AthDHS2". Please revise the statement in row 30.

Row 36: "was observed in young seedlings". What about mature plants? It feels like it was missed from the abstract.

Introduction:

Row 158: I recommend including the individual molecule-effects mention in the introduction, such as caffeate in Figure 1 and not only in the summery Figure 11. It will be easier for the readers to link the function of Phe downstream compounds.

Results:

Row 288-291: What is the difference between Figure 4B and Figure 4C? Some intermediate compounds are overlapped.

Consider moving Figure 7 into the supplementary. The main results (with 300 uM) are presented in Figure 8. There is some redundancy between the two figures, and 8 is more informative.

Figure 9: can you explain why *dhs-1* had shown a stronger phenotype then *dhs-3*? Is this due to the different abundance between the two shown by rt-PCR? (DHS1 is predominant)?

Row 428-434: I suggest to mention EPSP synthase in Figure 1, which help the readers to understand the shikimate is an upstream product and accumulate upon glyphosate application.

Row 438-442: the *dhs1*+Tyr are confusing. The expected results that it will be lower than the *dhs2*+Tyr. The statement "elevated shikimate levels but due to its small shoot size" is problematic because it is expected that the data was normalized to g FW. If the leaves are too small and showed a phenotype that makes then incomparable to *dhs2* and *dhs3*, another approach is needed to compare between the three enzymes. Else, from this figure, we can conclude that *dhs1* is also inhibited by Tyr.

Row 479, Table S2, and 3: the tables refer to each other, and ANOVA, $p<0.05$ was used. Why a one-way ANOVA was used and not two ways? (comparing treatment HL versus under standard growth condition and four genotypes)

Row 474 - Why did you measured Phe-derived and not Tyr-derived compounds (such as tocopherols)? The induction toward phenylpropanoid compounds might reduce the Tyr levels and might affect the DHS2 activity. Table S3 suggested the opposite (no depletion of Tyr).

Discussion:

Row 589: what does it mean "young plants"? Some experiments were conducted on etiolated plants while other on seedlings and four-week-old plants.

Reviewer #2:

This study provides an in-depth characterization of the shikimate pathway regulation in Arabidopsis. The authored focus on the relatively less characterized DHS enzymes. This characterization revealed that the DHS enzymes

regulation in plants is different and far more complex than the one described for microbes. I share the authors' view that these findings could lead to new strategies of controlling the carbon flux through the shikimate pathway and its subsequent downstream natural products that are important for both plants and humans. In general, the authors provided a very thorough characterization of three DHS enzymes and their potential effectors in vitro and in planta. The findings also revealed very intriguing interactions between the enzymes that may be relevant in other metabolic pathways. The study also shed light on the sometimes elusive interactions between the primary pathway and its secondary downstream products. The latter is a key to understanding the complexity of plant metabolism. Overall, the authors conducted a broad biochemical and genetic analyses and touched on the potential biological relevance and function of their discoveries. These important findings would help advance our general understanding of metabolic pathway regulations in plants. Below are some specific comments:

Major:

1. The authors discussed the expression of the three enzymes in the plant, but did not provide a more comprehensive gene expression analysis. I believe this could help the readers follow the author's arguments and potentially help improve their understanding of the unique function of these enzymes, (which was eluded in the stress experiments). This could be easily taken from the public databases and be presented as a table or a heatmap.

2. I wonder why the authors did not perform a DHS enzyme activity assay in the dhs1 mutants. According to their hypothesis, the DHS activity should be attenuated in the absence of DHS1 by means of applying proper effectors even in mature leaves. performing enzyme activity could provide a more direct evidence of the potential interaction between DHS1 and 2 and can strengthen the authors' arguments and genetics findings. Nevertheless if there was a reason they should explain it in their manuscript.

Minor:

1. The authors did a very thorough analysis of effectors from the pathway, although not essential, I wonder why they did not test other amino acids as effectors. It is well documented that there are many cross talks between many amino acid pathways.

2. The authors did mention that the nature of the DHS2 lack of inhibition by AAA in the presence of DHS1 is unclear. However, I wonder if there are any similar cases in the literature that could be cited to provide some potential explanation.

3. The authors discussed the high concentration in which AAAs affect the DHS activities and suggested that this is due to a second layer of regulation of this pathway. It would be useful to describe when such AAA concentration would be found in a plant.

4. The authors claimed that they measured more shikimate in both *dhs1* and *2* mutants but claimed that in the case of *dhs1*, it was due to a small size. I think it would be worthwhile to explain this claim.

<u>Reviewer #3:</u>

I have reviewed the manuscript entitled « The Entry Reaction of the Plant Shikimate Pathway Is Subjected to Highly-Complex Metabolite-Mediated Regulation » by Yokoyama et al. submitted for publication in The Plant Cell.

The study tackles the relatively understudied, yet very important question of how plant central and specialized metabolisms are interconnected and instruct each other to coordinate carbon flux under various developmental and environmental contexts. Here, authors focus on the 3-deoxy-D-arabino-heptulosonate 7-phosphate synthase (DHS) enzyme that catalyzes the first committed step of aromatic amino acids (AAAs) biosynthesis (aka the shikimate pathway). First part of the study is dedicated to the biochemical characterization of the three *Arabidopsis thaliana* DHS enzymes in vitro. After setting up proper reaction conditions and determining the catalytic parameters of recombinant enzymes, authors start to explore the feedback regulation of DHS activity by downstream metabolites. Data point to a complex, sometimes isoform-specific regulation of DHS activity. In short, all three DHS were inhibited by chorismate (a hub molecule within the shikimate pathway), caffeate and caffeoyl-shikimate (two phenylpropanoids), while only DHS2 was feedback inhibited by some AAAs (Tyr and Trp). Noteworthy, DHS2 was also sensitive to redox. Authors then

continue with in planta experiments: qRT-PCR expression analysis, DHS activity in plant protein extract, *dhs* mutant isolation coupled to phenotypic characterization, pharmacological and stress treatments, and finally metabolic profiling. Data show that *dhs1* is sensitive to exogenous application of Tyr (WT and *dhs3* also, to a much lesser extent), which, by authors words, can be prevented by lack of DHS2 enzyme. Finally, authors show that some phenylpropanoids, which derive from Phe, are slightly less abundant in all three single *dhs* mutant under high-light, a condition known to boost this metabolic pathway.

Overall, this study provides a massive amount of high-quality data that address an important question: how central and specialized metabolic pathways are coordinated? This well-written manuscript combines in vitro and in planta investigations to grasp the complexity of DHS regulation. I really appreciate author efforts to conduct both types of experiments. Biochemical data are beautiful and compelling. I hereafter have some comments that can serve as a platform for discussion/improvement of the manuscript:

1- *AthDHS* expression patterns

I feel that expression profiles of *AthDHS* genes could have been more thoroughly characterized by sampling a broader set of tissues for qPCR and/or generating GUS reporter lines. Catalytic specialization is often accompanied by expression pattern specialization. This is supported by coexpression data (supp. Table S1) that clearly suggest different function for the three *AthDHS* genes. Expression patterns could in turn be used to guide in planta analysis to tissues/organs relevant to each isoform. For instance, DHS3 is coexpressed with a number of phenylpropanoid genes, which calls for investigation in "phenolic" active tissues such as stems. I would not be surprised if DHS3 is indeed highly expressed there.

2- *A. thaliana AthDHS2*-dependent hypersensitivity to Tyr

In my view, there is not sufficient support to the fact plant Tyr hypersensitivity is due to DHS2. Side-by-side growth experiment (fig. 9A) only cannot lead to the conclusion DHS2 is involved in this phenotype. What about generating *dhs1/dhs2* and *dhs3/dhs2* double mutants and see whether they are hypersensitive to Tyr? If this phenotype is due to DHS2, then double mutants should be insensitive to Tyr.

3- Manuscript leans toward biochemistry

As written above, in vitro biochemistry reported in the manuscript is very robust and compelling. In planta studies, which were carefully performed, are comparatively not as thorough as the in vitro ones. This results in a marked "biochemistry spin" of the manuscript. This is not an issue per se, but it translates into difficulty to draw conclusions from in planta data and to feed accordingly the working model. I wonder whether in planta labeling and tracing experiments in WT and mutant plants would not bring better information on how metabolic flux is sorted in the shikimate pathway than pharmacological approaches.

Other comments:

L. 192-202: These are control data that break the flow of the results section; I would condense this paragraph in one or two sentences and put it earlier in the section.

L. 218: please specify in the text that it is 1 mM of each amino acid

L. 233 and thereafter: I do not have any problem with the term "effector molecule", but I do not see its benefit over the "inhibitor" term that also avoids the confusion with effector molecules in the context of plant-microbe interactions.

L. 303: please specify which tissue/organ was sampled for total protein extraction.

L. 433-435: using glyphosate-induced shikimate accumulation as a reporter of metabolic flux implies that there is no feedback regulation upon glyphosate treatment. Is it really the case?

L. 442-444: could we imagine that DHS2-containing protein heterocomplexes are destabilized or inhibited?

L. 458-473: the whole paragraph is dispensable since data only concern DHS1 isoform and are not informative. Data can be provided as supplemental data.

L. 505: I might be wrong, but I did not find any data supporting the fact DHS enzymes are "sensitive" to E4P concentration. Authors probably refer to the fact DHS Km for E4P is higher in plants than in microbes, but it remains to demonstrate that the enzyme is particularly "sensitive" to this molecule as compared to PEP.

L. 533-534: It is very interesting the DHS2 sis found redox-dependent. Did the authors check in their multiple-sequence alignment for the presence of discriminant cysteine residues in DHS2 proteins?

In line of this comment, it would have been great to have an alignment of representative DHS1, 2,3 proteins as supplemental data.

L. 861-863: please describe the LC-MS setup. Did the authors confirm peak identity with authentic standards?

Fig. 10C, D: please replace "flavnol" with "flavonol"

Fig. S1: please display tree as a cladogram since the use of a quite distant outgroup sequence "squeezes" plant DHS tree. Bootstraps support is usually relatively low (<75) on main branches; did the authors removed ambiguous sites prior to phylogeny reconstruction? which alignment algorithm was use? Please update corresponding Mat & Meth section.

Table S1: It would be great to exploit a bit more the full coexpression data (not only top 20 hits), e.g. GO terms enrichment analysis, to extract the meaningful information.

**TPC2020-RA-00840 Submission received**	**Oct. 7, 2020**

Reviewer comments on previously declined manuscript and **author responses:**

Editors:

As you'll see when you read their comments, all three reviewers have provided careful and thorough reviews. Although we would like you to address each of their comments in your response to reviews, we felt that several items are of particular importance to improve your manuscript.

First, Reviewer 1 asks about the use of the term "young plants" and we agree that this is problematic because it conflates age, tissue type, and developmental state in a way that is confusing (e.g. some may be tempted to think about phase change in this context). Please consider how you can rephrase all of these results and discussion for clarity.

> **Response 01: We agree that the term "young plant" is ambiguous. In the revised manuscript, we used more specific terms, such as "young seedling" or "etiolated/de-etiolated seedlings", in the revised version of the manuscript.**

Second, two of the reviewers ask for more information on DHS isoform expression, suggesting that this information can be gleaned from publicly available databases. Adding this information would be straightforward and helpful.

> **Response 02: Thank you for the suggestion. We have compiled information of DHS isoform expression from publicly available databases (e.g. Genevestigator and eFP browser), which are now presented in Supplemental Figure 9. Several relevant sections in the manuscript text were also revised accordingly.**

Third, Reviewer 2 also suggests that DHS activity assays in the *dhs1* mutant background would provide a valuable "in vivo" test of your in vitro results. Completing these experiments should not delay a resubmission substantially.

> **Response 03: We agreed that this is a critical experiment. DHS activity was further analyzed from the *dhs1* mutant along with Col-0 wild-type and other two *dhs* mutants. The new data are now shown in Supplementary Figure 15. Please see our detailed responses to the reviewer 2's comment below (Response 17).**

In contrast, although the analysis of double mutants might shed light on the questions you are asking and could be included in a revision if they are available, we do not want to delay your resubmission to the degree that their generation would require.

> **Response 04: We appreciate the editor for your balanced judgement and supportive comment. As we mention in Response 23 below, we are currently generating the double mutants but it is taking an extra time due to strong genetic linkage and potential lethality, as well as our limited working hours and capacity due to the pandemic. Thus, we decided to present the results in a future manuscript.**

Reviewer #1:

This study brings new findings in eukaryotes shikimate pathway regulation. It expands our understanding of the shikimate biosynthesis and the allosteric regulation by its intermediates, Tyr, Trp, and downstream products from the phenylpropanoids. It includes an extensive amount of biochemical and analytical chemistry analyses (in Arabidopsis and spinach) and used Arabidopsis mutants to understand the different regulation of each one of the DHS isozymes.

I have only minor comments:

Abstract:

Row 30: "are rarely inhibited by AAAs" - if this is the case, why it was studied in here? In row 32 you mention that "found that tyrosine and tryptophan inhibit AthDHS2". Please revise the statement in row 30.

Response 05: Thank you for this comment. We revised the abstract to emphasize that "Here we characterized *Arabidopsis thaliana* DHS (AthDHS) recombinant enzymes". Unlike prior results on DHS activity assays from "plant extracts", which were rarely inhibited by AAAs, this study found that the AthDHS2 "recombinant enzyme" is inhibited by AAAs. We hope this will clarify the confusion.

Row 36: "was observed in young seedlings". What about mature plants? It feels like it was missed from the abstract.

Response 06: We included an additional phrase, "but not in mature leaves where *AthDHS1* is dominantly expressed.". The other sentences of the abstract were revised to keep it within the 200-word limit.

Introduction:

Row 158: I recommend including the individual molecule-effects mention in the introduction, such as caffeate in Figure 1 and not only in the summery Figure 11. It will be easier for the readers to link the function of Phe downstream compounds.

Response 07: Figure 1 was revised to include key molecules studied in this work (e.g. caffeate).

Results:

Row 288-291: What is the difference between Figure 4B and Figure 4C? Some intermediate compounds are overlapped.

Response 08: In Figure 4B, we initially tested commercially available phenylpropanoids as the effector candidates and discovered the strong inhibitory effect of caffeate (but not the other phenylpropanoids) on all AthDHS isoforms. Given the importance of the 4-hydroxyl group in the benzene ring, we then tested other phenylpropanoid chemicals with hydroxyl groups at the 3'- and 4'-positions of the ring in Figure 4C. *p*-Coumarate and caffeate are also shown in Figure 4C, besides Figure 4B, but as they were tested again as negative and positive controls. This also demonstrates robustness among different experiments. If necessary, however, we are happy to remove some of the redundancy.

Consider moving Figure 7 into the supplementary. The main results (with 300 μM) are presented in Figure 8. There is some redundancy between the two figures, and 8 is more informative.

Response 09: Thanks for your suggestion. We now moved Figure 7 to Supplemental Figure 10.

Figure 9: can you explain why *dhs-1* had shown a stronger phenotype than *dhs-3*? Is this due to the different abundance between the two shown by rt-PCR? (DHS1 is predominant)?

Response 10: Yes, we also think that the stronger phenotype of *dhs1* is due to its predominant expression of *AthDHS1* than *AthDHS3*, and its deficiency has much more drastic effects.

Row 428-434: I suggest to mention EPSP synthase in Figure 1, which help the readers to understand the shikimate is an upstream product and accumulate upon glyphosate application.

Response 11: Thanks for the suggestion. We added EPSPS and glyphosate to the pathway map in Figure 1.

Row 438-442: the *dhs1*+Tyr are confusing. The expected results that it will be lower than the *dhs2*+Tyr. The statement "elevated shikimate levels but due to its small shoot size" is problematic because it is expected that the data was normalized to g FW. If the leaves are too small and showed a phenotype that makes then incomparable to *dhs2* and

dhs3, another approach is needed to compare between the three enzymes. Else, from this figure, we can conclude that *dhs1* is also inhibited by Tyr.

Response 12: Thanks for bringing up this point. The alternative approach of normalization is to present a relative increase in the shikimate levels before and after the glyphosate treatment. However, such a presentation will eliminate some important aspects of our observations (e.g. actual levels observed). Therefore, we kept the figure as it is, but discussed the relative increase in the shikimate levels before and after the glyphosate treatment in the presence of tyrosine. In short, much more shikimate accumulated in *dhs2* (~ 5-fold) than *dhs1* and other genotypes (~3-fold) after the glyphosate treatment specifically in the presence, but not in the absence, of tyrosine. The relevant manuscript text was also revised accordingly.

Row 479, Table S2, and 3: the tables refer to each other, and ANOVA, $p<0.05$ was used. Why a one-way ANOVA was used and not two ways? (comparing treatment HL versus under standard growth condition and four genotypes)

Response 13: We combined S2 and S3 into one table, followed by two-way ANOVA, to compare the four genotypes under two different treatment conditions.

Row 474 - Why did you measured Phe-derived and not Tyr-derived compounds (such as tocopherols)? The induction toward phenylpropanoid compounds might reduce the Tyr levels and might affect the DHS2 activity. Table S3 suggested the opposite (no depletion of Tyr).

Response 14: We now conducted a new experiment to analyze levels of tocopherols in Col-0 and the *dhs* mutants before and after 2-day high light treatment. Under the standard growth condition, the levels of alpha- and gamma-tocopherols are comparable between Col-0 and the *dhs* mutants (Supplemental Table 2A). HL treatment increased the amount of the tocopherols, but at the same levels among all *dhs* mutants and Col-0 (Supplemental Table 2B). This result suggests that the *dhs* mutations had limited impacts on the levels of these Tyr-derived compounds.

Discussion:

Row 589: what does it mean "young plants"? Some experiments were conducted on etiolated plants while other on seedling and four-week-old plants.

Response 15: Thank you for the comment. We now use more specific terms, such as “young seedling” or “etiolated/de-etiolated seedlings” instead of “young plants” throughout the manuscript.

<u>Reviewer #2:</u>

This study provides an in-depth characterization of the shikimate pathway regulation in Arabidopsis. The authored focus on the relatively less characterized DHS enzymes. This characterization revealed that the DHS enzymes regulation in plants is different and far more complex than the one described for microbes. I share the authors' view that these findings could lead to new strategies of controlling the carbon flux through the shikimate pathway and its subsequent downstream natural products that are important for both plants and humans. In general, the authors provided a very thorough characterization of three DHS enzymes and their potential effectors in vitro and in planta. The findings also revealed very intriguing interactions between the enzymes that may be relevant in other metabolic pathways. The study also shed light on the sometimes elusive interactions between the primary pathway and its secondary downstream products. The latter is a key to understanding the complexity of plant metabolism. Overall, the authors conducted a broad biochemical and genetic analyses and touched on the potential biological relevance and function of their discoveries. These important findings would help advance our general understanding of metabolic pathway regulations in plants. Below are some specific comments:

Major:

1. The authors discussed the expression of the three enzymes in the plant, but did not provide a more comprehensive gene expression analysis. I believe this could help the readers follow the authors’ arguments and potentially help improve their understanding of the unique function of these enzymes, (which was eluded in the stress experiments). This could be easily taken from the public databases and be presented as a table or a heatmap.

Response 16: Thank you for the suggestion. We have compiled expression profiles of three *AthDHS* isoforms from different databases and provided in Supplemental Figure 9. These data suggest that *AthDHS1* and *AthDHS3* are

strongly induced upon various stresses (e.g. pathogen attacks) while *AthDHS2* is more constitutively expressed throughout different tissues and developmental stages, with its strong expression in young seedlings and during senescence (e.g. abscission zone). However, there are many overlapped expressions among different *AthDHS* isoforms, suggesting that different isoforms have both distinct and overlapping roles.

These data are described and discussed in multiple sections of the manuscript.

2. I wonder why the authors did not perform a DHS enzyme activity assay in the *dhs1* mutants. According to their hypothesis, the DHS activity should be attenuated in the absence of DHS1 by means of applying proper effectors even in mature leaves. performing enzyme activity could provide more direct evidence of the potential interaction between DHS1 and 2 and can strengthen the authors' arguments and genetics findings. Nevertheless if there was a reason they should explain it in their manuscript.

Response 17: We would like to thank the reviewer for this suggestion. We now isolated crude extracts from Col-0 and the *dhs1* (as well as *dhs2* and *dhs3*) mutants and conducted their DHS enzyme assay with individual AAA. The data showed that the DHS activities detected from mature leaf extracts of all *dhs* mutants were not significantly different from that of Col-0 even in the presence of Tyr or Trp (which is now presented in Supplemental Figure 15). Based on this observation, we then hypothesized that the remaining AAA-insensitive AthDHS (i.e. AthDHS3 in *dhs1*) may be masking the effect of AthDHS2-mediated inhibition in the leaf crude extract.

To further test this hypothesis, we mixed AthDHS2 and AthDHS3 recombinant enzymes and conducted an enzymatic assay. Without any effectors or with Phe, the observed DHS activity in the various AthDHS2 and AthDHS3 mixtures matched the theoretical plot (black and purple lines, respectively, in Figure 5D). Notably, in the presence of 1 mM Tyr and Trp, however, observed DHS activity was higher than theoretically calculated activities in the AthDHS2 and AthDHS3 mixtures (orange and magenta lines, respectively, in Figure 5D), which is similar to the mixture experiment conducted earlier for AthDHS1 and AthDHS3 (Figure 5C). This result suggests that, like AthDHS1, AthDHS3 is able to mask the effect of inhibition of AthDHS2 by AAAs, which likely still kept the DHS activity of the *dhs1* crude extracts insensitive to AAAs. These two new results and associated discussions are now described in the manuscript.

Minor:

1. The authors did a very thorough analysis of effectors from the pathway, although not essential, I wonder why they did not test other amino acids as effectors. It is well documented that there are many cross talks between many amino acid pathways.

Response 18: We conducted enzymatic assays of all recombinant AthDHS enzymes (AthDHS1, 2 and 3) in the presence of all 20 proteinogenic amino acids individually. While Tyr and Trp significantly inhibited AtDHS2 again, the other amino acids at 1 mM do not significantly increase or reduce activities of any AthDHS isoforms, suggesting that Tyr and Trp specifically inhibit the AthDHS2 enzyme. This new data is now presented in Supplemental Figure 5.

2. The authors did mention that the nature of the DHS2 lack of inhibition by AAA in the presence of DHS1 is unclear. However, I wonder if there are any similar cases in the literature that could be cited to provide some potential explanation.

Response 19: Chen et al., reported that poplar 4-coumaric acid:CoA ligase 5 (4CL5) has different substrate specificity from 4CL3 and forms a heterocomplex with 4CL3 to change substrate preference of 4CL3. This interaction affects the direction and rate of metabolic flux for monolignol biosynthesis. We introduce this example (lines 565-568) as a case that a certain isoform affects the functions of another isoform.

3. The authors discussed the high concentration in which AAAs affect the DHS activities and suggested that this is due to a second layer of regulation of this pathway. It would be useful to describe when such AAA concentration would be found in a plant.

Response 20: Thanks for this question. Drastic elevations of free amino acid levels were observed under senescence, where protein synthesis is slowed and degradation is elevated, including in the degradation of plastid proteins via various proteases. Consistently, public transcriptome information suggests that *AthDHS2* expression is upregulated during senescence (see our new Supplemental Figure 9). Also, there may be a "transient" spike in AAA levels under certain conditions (e.g. upon developmental transition). Under these conditions multiple negative feedback inhibition systems including the first reaction catalyzed by DHS enzymes may need to be regulated efficiently. The manuscript was revised to discuss these points.

4. The authors claimed that they measured more shikimate in both *dhs1* and *2* mutants but claimed that in the case of *dhs1*, it was due to a small size. I think it would be worthwhile to explain this claim.

Response 21: Thank you for this comment. We provided an additional explanation in the manuscript. Also, please see our detailed response to Reviewer 1 above (Response 10).

Reviewer #3:

I have reviewed the manuscript entitled « The Entry Reaction of the Plant Shikimate Pathway Is Subjected to Highly-Complex Metabolite-Mediated Regulation » by Yokoyama et al. submitted for publication in The Plant Cell.

The study tackles the relatively understudied, yet very important question of how plant central and specialized metabolisms are interconnected and instruct each other to coordinate carbon flux under various developmental and environmental contexts. Here, authors focus on the 3- deoxy-D-arabino-heptulosonate 7-phosphate synthase (DHS) enzyme that catalyzes the first committed step of aromatic amino acids (AAAs) biosynthesis (aka the shikimate pathway). First part of the study is dedicated to the biochemical characterization of the three Arabidopsis thaliana DHS enzymes in vitro. After setting up proper reaction conditions and determining the catalytic parameters of recombinant enzymes, authors start to explore the feedback regulation of DHS activity by downstream metabolites. Data point to a complex, sometimes isoform- specific regulation of DHS activity. In short, all three DHS were inhibited by chorismate (a hub molecule within the shikimate pathway), caffeate and caffeoyl-shikimate (two phenylpropanoids), while only DHS2 was feedback inhibited by some AAAs (Tyr and Trp).

Noteworthy, DHS2 was also sensitive to redox. Authors then continue with in planta experiments: qRT-PCR expression analysis, DHS activity in plant protein extract, *dhs* mutant isolation coupled to phenotypic characterization, pharmacological and stress treatments, and finally metabolic profiling. Data show that *dhs1* is sensitive to exogenous application of Tyr (WT and *dhs3* also, to a much lesser extent), which, by authors words, can be prevented by lack of DHS2 enzyme. Finally, authors show that some phenylpropanoids, which derive from Phe, are slightly less abundant in all three single *dhs* mutant under high-light, a condition known to boost this metabolic pathway.

Overall, this study provides a massive amount of high-quality data that address an important question: how central and specialized metabolic pathways are coordinated? This well-written manuscript combines in vitro and in planta investigations to grasp the complexity of DHS regulation. I really appreciate author efforts to conduct both types of experiments. Biochemical data are beautiful and compelling. I hereafter have some comments that can serve as a platform for discussion/improvement of the manuscript:

1- *AthDHS* expression patterns

I feel that expression profiles of *AthDHS* genes could have been more thoroughly characterized by sampling a broader set of tissues for qPCR and/or generating GUS reporter lines. Catalytic specialization is often accompanied by expression pattern specialization. This is supported by coexpression data (supp. Table S1) that clearly suggest different function for the three *AthDHS* genes. Expression patterns could in turn be used to guide in planta analysis to tissues/organs relevant to each isoform. For instance, *DHS3* is coexpressed with a number of phenylpropanoid genes, which calls for investigation in "phenolic" active tissues such as stems. I would not be surprised if DHS3 is indeed highly expressed there.

Response 22: Thank you for your positive feedback on our study as well as this specific comment. Following the suggestions from the editor and Reviewer 1, we compiled expression profiles of *AthDHS* isoforms from different databases (see new Supplemental Figure 9). These data suggest that *AthDHS1* and *AthDHS3* are strongly induced upon various stresses (e.g. pathogen attacks). Consistent with the Reviewer 3's expectation, *AthDHS3*, an isoform coexpressed with phenylpropanoid biosynthetic genes, is strongly expressed in stem tissues where phenylpropanoid production actively takes place. On the other hand, *AthDHS2* is more constitutively expressed throughout different tissues and developmental stages, with its strong expression in young seedlings and during senescence. However, there are many overlapped expressions among different *AthDHS* isoforms. These data are described in multiple sections of the manuscript to discuss some specific but often overlapping functions of *AthDHS* isoforms.

2- *A. thaliana AthDHS2*-dependent hypersensitivity to Tyr

In my view, there is not sufficient support to the fact plant Tyr hypersensitivity is due to DHS2. Side-by-side growth experiment (fig. 9A) only cannot lead to the conclusion DHS2 is involved in this phenotype. What about generating *dhs1/dhs2* and *dhs3/dhs2* double mutants and see whether they are hypersensitive to Tyr? If this phenotype is due to DHS2, then double mutants should be insensitive to Tyr.

Response 23: We thank the reviewer for suggesting the isolation and analysis of the *dhs* double mutants. Indeed, we are currently making the double mutants of different *dhs* mutants. However, some genes (i.e. *AthDHS1* and *2*) are tightly linked on the same chromosome and some mutant combinations appear to be lethal (pending a larger and more thorough screening). Additionally, given the slowdown of our lab operation due to the pandemic, it will likely take many more months, if not a year, to isolate higher order mutants and properly characterize them. Therefore, following the editor's recommendation above, we decided to report the isolation and characterization of double mutants in a future manuscript (see Response 04).

3- Manuscript leans toward biochemistry

As written above, in vitro biochemistry reported in the manuscript is very robust and compelling. In planta studies, which were carefully performed, are comparatively not as thorough as the in vitro ones. This results in a marked "biochemistry spin" of the manuscript. This is not an issue per se, but it translates into difficulty to draw conclusions from in planta data and to feed accordingly the working model. I wonder whether in planta labeling and tracing experiments in WT and mutant plants would not bring better information on how metabolic flux is sorted in the shikimate pathway than pharmacological approaches.

Response 24: Thank you for the comment. We are planning to set up an in-house system for $^{13}CO_2$-labeling experiments to directly monitor carbon flux through the shikimate pathway in plants. Unfortunately, we have been experiencing multiple hurdles on obtaining all necessary parts to assemble the set up due to the pandemic. Thus, we hope to carry out these quantitative analyses along with the higher order mutants and report these results in our future manuscript.

Other comments:

L. 192-202: These are control data that break the flow of the results section; I would condense this paragraph in one or two sentences and put it earlier in the section.

Response 25: We shortened and combined this paragraph into an earlier section.

L. 218: please specify in the text that it is 1 mM of each amino acid

Response 26: Done.

L. 233 and thereafter: I do not have any problem with the term "effector molecule", but I do not see its benefit over the "inhibitor" term that also avoids the confusion with effector molecules in the context of plant-microbe interactions.

Response 27: We have converted "effector molecule", into "inhibitor" wherever we can; however, in some cases, such replacement caused an issue. For example, arogenate was not an inhibitor but rather an effector molecule that offsets chorismate-dependent inhibition (Figure 3C and D). In these instances, we still kept the term "effector molecule" in the manuscript in order to avoid inaccurate descriptions of our findings. We avoided the use of the term "effector" in the title, subtitles, and abstract, where general readers likely read without going into details, in order to avoid any potential confusion. We hope these changes will address your concern.

L. 303: please specify which tissue/organ was sampled for total protein extraction.

Response 28: Fully expanded mature leaves of Arabidopsis and spinach were used to isolate crude extracts. We modified the corresponding sentences as well as materials and methods.

L. 433-435: using glyphosate-induced shikimate accumulation as a reporter of metabolic flux implies that there is no feedback regulation upon glyphosate treatment. Is it really the case?

Response 29: To our knowledge and based on our current results, no intermediates upstream of EPSP synthase (e.g. shikimate) have been shown to inhibit upstream enzymes including DHS enzymes in plants. Therefore, it is unlikely that the glyphosate treatment itself induced a feedback regulation. In our revised manuscript, we further confirmed that introducing the wild type *AthDHS2* gene in the *dhs2* mutant can revert the glyphosate-induced shikimate accumulation in the presence of Tyr back to the Col-0 level, which is now presented in Supplemental Figure 14B.

L. 442-444: could we imagine that DHS2-containing protein heterocomplexes are destabilized or inhibited?

Response 30: Thank you for raising this interesting point. Our biochemical analysis of AthDHS2 enzyme activity in the presence of AthDHS1 or AthDHS3 (Figure 5C and D) suggest that AtDHS1 and AthDHS3 somehow attenuate Tyr and Trp-mediated inhibition of AthDHS2 likely through heterocomplex formation. We saw no evidence of destabilization or stabilization at least in vitro, as the mixing of AthDHS2 with AthDHS1 or 3 did not alter overall activity in the absence of inhibitors (i.e. Try or Trp).

L. 458-473: the whole paragraph is dispensable since data only concern DHS1 isoform and are not informative. Data can be provided as supplemental data.

Response 31: Thank you for the suggestion. We significantly shortened the description and all data are provided in supplemental data.

L. 505: I might be wrong, but I did not find any data supporting the fact DHS enzymes are "sensitive" to E4P concentration. Authors probably refer to the fact DHS Km for E4P is higher in plants than in microbes, but it remains to demonstrate that the enzyme is particularly "sensitive" to this molecule as compared to PEP.

Response 32: Thank you for pointing this out. We agree that the term "sensitive" in the previous lines 505 and 521 is misleading. To this end, we revised these phrases and instead described that plant DHS enzymes have high apparent *K*m towards E4P.

L. 533-534: It is very interesting the DHS2 sis found redox-dependent. Did the authors check in their multiple-sequence alignment for the presence of discriminant cysteine residues in DHS2 proteins? In line of this comment, it would have been great to have an alignment of representative DHS1, 2,3 proteins as supplemental data.

Response 33: We are indeed interested in the different redox sensitivity among the isoforms and how this isoform specific property evolved in the plant kingdom. We are currently conducting enzyme characterization of AthDHS2 orthologs from other plant species, from which we will design mutagenesis experiments to determine Cys residue(s) that may be responsible for the redox dependency. We hope to report these results in a future manuscript.

L. 861-863: please describe the LC-MS setup. Did the authors confirm peak identity with authentic standards?

Response 34: We conducted MS/MS fragmentation experiments to confirm the identity of almost all compounds, which were consistent with previously reported methods that we followed and cited. These data are now described in Supplemental Table 4. The identity of Trp and indolyl-3-methyl glucosinolate (I3M) peaks were confirmed by comparing their accurate masses and retention times with those of the corresponding authentic standards.

Fig. 10C, D: please replace "flavnol" with "flavonol"

Response 35: The typo has been corrected. Thank you.

Fig. S1: please display tree as a cladogram since the use of a quite distant outgroup sequence "squeezes" plant DHS tree. Bootstraps support is usually relatively low (<75) on main branches; did the authors removed ambiguous sites prior to phylogeny reconstruction? which alignment algorithm was use? Please update corresponding Mat & Meth section.

Response 36: Thanks for your suggestion. In the previous manuscript, we used the ClustalW algorithm and did not remove any sites from the sequences. The same set of sequences, which is now provided in Supplemental Data Set 3, are now also aligned by the Muscle algorithm to construct a new cladogram tree using the maximum-likelihood method with all positions containing gaps and missing data were eliminated. The new result gave overall similar result with slightly better bootstraps values. The new cladogram tree is now presented in Supplemental Figure 1. We revised and provided more information in the corresponding section of Material and Method.

Table S1: It would be great to exploit a bit more the full coexpression data (not only top 20 hits), e.g. GO terms enrichment analysis, to extract the meaningful information.

Response 37: Using more extended coexpression datasets (top 1000 hits) of *AthDHS1*, *AthDHS2* and *AthDHS3* from Atted-II database, we performed their GO enrichment analysis. Although coexpression lists of *AthDHS1* and *AthDHS3* exhibited enrichment of GO terms related to biosynthesis of AAA and AAA-derived metabolites, that of *AthDHS2* was enriched with biological processes involved in plastid development and photosynthesis (Supplemental Figure 8). These results suggest that, unlike *AthDHS1* and *AthDHS3*, *AthDHS2* likely plays critical roles during chloroplast development. The full dataset of the GO enrichment analysis is also provided in Supplemental Data Set 2.

TPC2020-RA-00840 1st Editorial decision – *accept with minor revision* **Nov. 6, 2020**

We have received reviews of your manuscript entitled "The Entry Reaction of the Plant Shikimate Pathway Is Subjected to Highly-Complex Metabolite-Mediated Regulation." On the basis of the advice received, the board of reviewing editors would like to accept your manuscript for publication in The Plant Cell. This acceptance is contingent on revision based on the comments of our reviewers. In particular, please consider the comments of Reviewer 1 and see if you can make minor text changes to clarify their issues of concern.

-- Reviewer comments:

Reviewer #1:

Overall the study is novel, interesting, and highlighting a new perspective on DHS regulation in plant systems. It expands our understanding of the shikimate biosynthesis and the allosteric regulation by its intermediates, Tyr, Trp, and downstream products from the phenylpropanoids. It includes an extensive amount of biochemical analyses and used Arabidopsis mutants and recovering phenotypes by overexpression to understand the different regulation of each one of the DHS isozymes. My previous comments were fully answered by the authors, and I have only minor comments.

Line 211 - Why is it important to study the effect of the AAA mix instead of the individual ones?
Line 324 - It is unclear why AAA sensitivity is related to the tetrameric or dimeric forms of the DHS protein?
Both caffeate and chorismate have dramatically inhibited the enzymes using 1 mM. Is it relevant to the physiological levels?
Figure 8 and L426-427- the cap shape can be related to the high levels of Tyr, while according to this manuscript, mature plants have a low level of DHS2. Therefore, the phenotype was not affected by exogenous supply Tyr. A similar change in leaf shape was previously reported when feedback insensitive bi-functional chorismate mutase/prephenate dehydratase was overexpressed (more Phe and downstream Tyr derivatives) in Arabidopsis plants. See Figure 4A PheA17 "which in some cases showed minor alterations in leaf structure when grown in soil for long periods". The Plant Journal, (2009) 60: 156-167.

Figure 9 - It is unclear how increasing the anthocyanin (Phe-derived) by HL, is relevant since the DHSs are affected by Tyr and Trp. Therefore, the three mutants possessed similar metabolite levels (as shown in the Figure). Why not to induce Trp-derived glucosinolates by damage or insect feeding then looking at the differences? Consider taking this figure out, or better explain the rationale.

Reviewer #3:

The authors have addressed all my comments in their responses, I have no further questions or concerns.

Reviewer #4:

Thanks to the authors for thoroughly addressing comments/questions raised in the previous round of reviewing. Manuscript now provide all the critical information the reader may need.

Last comment: It is a pity that eFP browser data are buried in Supplemental files. I'd rather have them, at least in part, incorporated to Figure 5 as they convey essential information. You may download absolute expression values from the "Developmental Map" series by clicking the appropriate tab beneath the image and draw a chart showing the expression level of the three *DHS* genes in e.g. 5-6 main Arabidopsis tissues.

TPC2020-RA-00840R1 1st Revision received **Nov. 18, 2020**

Reviewer comments on previous submission and **author responses**:

Reviewer #1:

Overall the study is novel, interesting, and highlighting a new perspective on DHS regulation in plant systems. It expands our understanding of the shikimate biosynthesis and the allosteric regulation by its intermediates, Tyr, Trp, and downstream products from the phenylpropanoids. It includes an extensive amount of biochemical analyses and used Arabidopsis mutants and recovering phenotypes by overexpression to understand the different regulation of each one of the DHS isozymes. My previous comments were fully answered by the authors, and I have only minor comments.

Line 211 - Why is it important to study the effect of the AAA mix instead of the individual ones?

Response 01: Since *Mycobacterium tuberculosis* DHS, the closest homolog of plant DHS, requires both Trp and Phe, but not either, for its inhibition (Webby et al., JBC, 2010), we initially speculated that the individual AAA was not sufficient to observe the negative inhibition of AthDHS enzymes. For this reason, we first tested AAA mixture to see if the negative feedback inhibition of AthDHSs by AAAs was detected or not, and then investigated the effect of the individual AAA on the AthDHS activities. This description was added in line 213.

Line 324 - It is unclear why AAA sensitivity is related to the tetrameric or dimeric forms of the DHS protein?

Response 02: We hypothesized that, if AthDHS enzymes with different AAA sensitivity were contained in the same heterocomplex, either isoform might affect the AAA sensitivity of the other isoform, changing overall sensitivity to AAAs. We described this in line 327.

Both caffeate and chorismate have dramatically inhibited the enzymes using 1 mM. Is it relevant to the physiological levels?

Response 03: It is unclear that concentrations of caffeate and chorismate are around their IC_{50} values of AthDHS enzymes (approximately 50 to 100 μM) in plant cells. We need to further analyze if these inhibitions occur in plants in the future.

Figure 8 and L426-427- the cap shape can be related to the high levels of Tyr, while according to this manuscript, mature plants have a low level of DHS2. Therefore, the phenotype was not affected by exogenous supply Tyr. A similar change in leaf shape was previously reported when feedback insensitive bi- functional chorismate mutase/prephenate dehydratase was overexpressed (more Phe and downstream Tyr derivatives) in Arabidopsis plants. See Figure 4A PheA17 "which in some cases showed minor alterations in leaf structure when grown in soil for long periods". The Plant Journal, (2009) 60: 156-167.

Response 04: Since the cap shape phenotype was observed in newly developing leaves rather than fully expanded mature leaves that were used for our qPCR analysis, the Tyr feeding did not induce the phenotype in such mature leaves where AthDHS2 is not highly expressed. We also appreciate the reviewer for introducing the paper showing the similar growth phenotype. We cited this manuscript (line 613).

Figure 9 - It is unclear how increasing the anthocyanin (Phe-derived) by HL, is relevant since the DHSs are affected by Tyr and Trp. Therefore, the three mutants possessed similar metabolite levels (as shown in the Figure). Why not to induce Trp-derived glucosinolates by damage or insect feeding then looking at the differences? Consider taking this figure out, or better explain the rationale.

Response 05: Initially, we conducted methyl jasmonate (MeJA) treatment, which was previously shown to strongly induce *AthDHS1* gene expression and the production of Trp- derived compounds. Although the levels of Trp-derived indole glucosinolates were clearly elevated after the MeJA treatment, the *dhs1* mutant and wild type plants showed very similar induction (as presented in Supplemental Figure 16). We then subjected the *dhs* mutants to HL treatment, which induces elevated production of both Tyr and Phe-derived metabolites, to access the role of different DHS isoforms in their production. We revised the manuscript text to make clear the rationale of the HL treatment experiment (line 502).

Reviewer #4:

Thanks to the authors for thoroughly addressing comments/questions raised in the previous round of reviewing. Manuscript now provide all the critical information the reader may need.

Last comment: It is a pity that eFP browser data are buried in Supplemental files. I'd rather have them, at least in part, incorporated to Figure 5 as they convey essential information. You may download absolute expression values from the "Developmental Map" series by clicking the appropriate tab beneath the image and draw a chart showing

the expression level of the three *DHS* genes in e.g. 5-6 main Arabidopsis tissues.

> **Response 06: We are grateful for this suggestion. We created a new graph from eFP browser that showed *AthDHS* gene expression in six different Arabidopsis tissues (Figure 5F).**

TPC2020-RA-00840R1 2nd Editorial decision – *acceptance pending* **Nov. 19, 2020**

We are pleased to inform you that your paper entitled "Title" has been accepted for publication in The Plant Cell, pending a final minor editorial review by journal staff. At this stage, your manuscript will be evaluated by a Science Editor with respect to scientific content presentation, compliance with journal policies, and presentation for a broad readership.

Final acceptance from Science Editor **Dec. 16, 2020**
